# Presynaptic Nrxn3 is essential for ribbon-synapse maturation in hair cells

**DOI:** 10.1242/dev.202723

**Published:** 2024-10-10

**Authors:** Alma Jukic, Zhengchang Lei, Elizabeth R. Cebul, Katherine Pinter, Yommi Tadesse, Amandine Jarysta, Sandeep David, Natalie Mosqueda, Basile Tarchini, Katie Kindt

**Affiliations:** ^1^ Section on Sensory Cell Development and Function, National Institute on Deafness and Other Communication Disorders, Bethesda, MD 20892, USA; ^2^ The Jackson Laboratory, Bar Harbor, ME 04609, USA; ^3^ Department of Developmental, Molecular and Chemical Biology, Tufts University School of Medicine, Boston, MA 02111, USA

**Keywords:** Neurexin3, Synapse maturation, Zebrafish, Hair cell, Sensory systems

## Abstract

Hair cells of the inner ear and lateral-line system rely on specialized ribbon synapses to transmit sensory information to the central nervous system. The molecules required to assemble these synapses are not fully understood. We show that Nrxn3, a presynaptic adhesion molecule, is crucial for ribbon-synapse maturation in hair cells. In both mouse and zebrafish models, the loss of Nrxn3 results in significantly fewer intact ribbon synapses. We show in zebrafish that, initially, Nrxn3 loss does not alter pre- and postsynapse numbers but, later, synapses fail to pair, leading to postsynapse loss. We also demonstrate that Nrxn3 subtly influences synapse selectivity in zebrafish lateral-line hair cells that detect anterior flow. Loss of Nrxn3 leads to a 60% loss of synapses in zebrafish, which dramatically reduces pre- and postsynaptic responses. Despite fewer synapses, auditory responses in zebrafish and mice are unaffected. This work demonstrates that Nrxn3 is a crucial and conserved molecule required for the maturation of ribbon synapses. Understanding how ribbon synapses mature is essential to generating new therapies to treat synaptopathies linked to auditory or vestibular dysfunction.

## Teaser

**Summary:** Hearing and balance depend on specialized ribbon synapses that transmit sensory stimuli between hair cells and afferent neurons. We identify Nrxn3 as a key molecular player in ribbon-synapse maturation in hair cells.

## INTRODUCTION

In the nervous system, synapses transmit signals between neurons, and proper synapse assembly is essential for the function of neural circuits. Although many players have been identified in synapse assembly in the central nervous system, relatively less is known about this process in hair cells, the sensory receptors of the inner ear. Synaptopathy, or synapse loss, can cause hearing loss or vestibular dysfunction (Liberman, 2017; Wan et al., 2019; Wu et al., 2020, 2021). Expanding our knowledge of the molecules involved in synapse assembly in hair cells is important for reforming these synapses and treating auditory and vestibular synaptopathy.

Hair cells convert sensory stimuli into signals that are sent to the brain. In response to sensory stimuli, apical structures called mechanosensory hair bundles are deflected in a direction-specific manner, opening mechanoelectrical transduction (MET) channels and depolarizing the cell. Depolarization opens voltage-gated calcium channels (Ca_V_1.3) at the presynapse, causing a calcium influx that triggers the release of glutamate onto afferent neurons (reviewed by Fettiplace, 2017). To properly encode sensory stimuli, hair cells use a specialized ribbon synapse for speed and precision (Khimich et al., 2005; Moser et al., 2006). A ribbon synapse is defined by a presynaptic density called a ribbon, which is composed primarily of the protein Ribeye (CTBP2 splice variant) (Schmitz et al., 2000). Ribbons function to recruit and tether synaptic vesicles at the presynaptic active zone.

The molecules involved in synapse maturation, maintenance and remodeling in hair cells remain largely undefined. Cell adhesion molecules (CAMs) are key modulators of synapse assembly, bridging pre- and postsynaptic domains (reviewed by Südhof, 2021). Recent work in mice found that a well-studied family of postsynaptic CAMs, neuroligins (Nlgns), are important for ribbon-synapse maturation (Ramirez et al., 2022). Mice lacking NLGN1/3 have fewer ribbon synapses in inner hair cells (IHCs), impaired hearing and heightened sensitivity to noise exposure. Postsynaptic neuroligins classically bind to a family of presynaptic CAMs called neurexins (Nrxns) (Südhof, 2021), but whether these neuroligins pair with a presynaptic neurexin in the auditory system is not known (Ramirez et al., 2022).

**Fig. 1.**
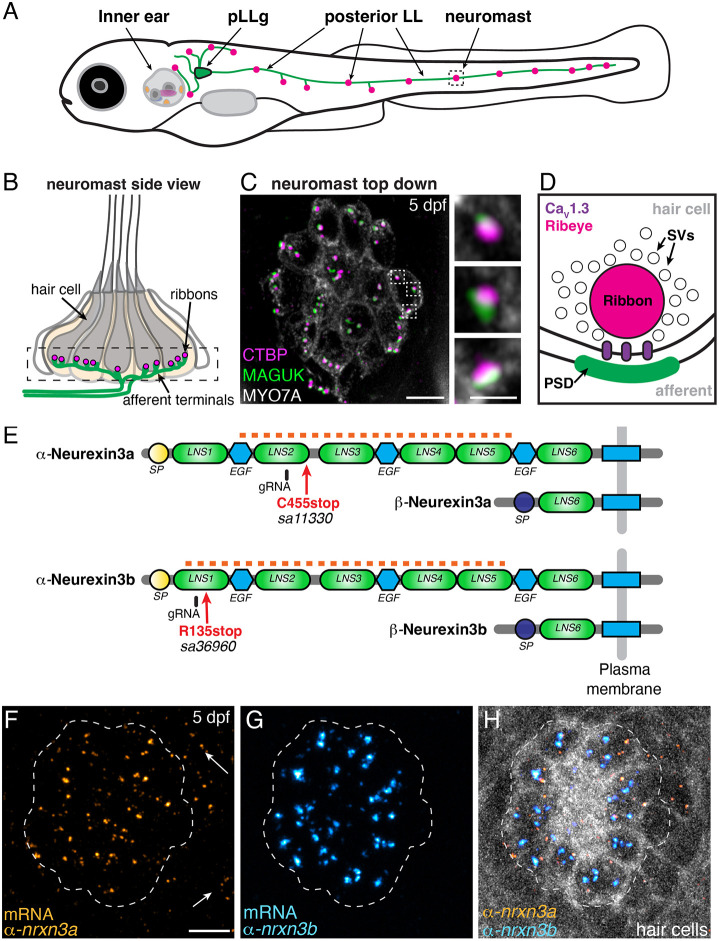
**α-*nrxn3a* and α-*nrxn3b* are expressed in lateral-line hair cells.** (A) Diagram of a 5 day post fertilization (dpf) larval zebrafish showing sensory hair-cell clusters in the inner ear and posterior lateral line (neuromasts). Neurons from the posterior lateral line ganglia (pLLg) innervate (pLLg, green) lateral-line neuromasts. (B) Side view of a neuromast organ showing hair cells, presynapses (ribbons) and afferent processes. The dashed box outlines the synaptic layer. (C) Immunostaining of the synaptic layer, viewed from above. CTBP and MAGUK stain presynapses/ribbons (magenta) and postsynapses (green), respectively. MYO7A stains hair cells (gray). Higher magnification of three synapses is shown on the right. (D) Diagram of a hair-cell ribbon synapse. The presynapse/ribbon consists mainly of Ribeye, a splice variant of CTBP2. The ribbon is surrounded by glutamate-filled synaptic vesicles (SVs). Ca_V_1.3 channels cluster beneath the ribbon, opposite the postsynaptic density (PSD). (E) Zebrafish have two orthologues of Nrxn3, Nrxn3a and Nrxn3b. Each neurexin has a long α form and a shorter β form. Red arrows show location of the mutations in germline zebrafish mutants; these lesions disrupt an obligatory exon in the α form of each orthologue (C455stop and R134stop). Location of gRNAs used in a crispant analysis are indicated. The α and β forms each have a unique start and signal peptide (SP). Each α form has six Laminin G-like domains (LNS) and three epidermal growth factor-like domains (EGF). The red dashed line indicates the location of the RNA FISH probes used in F-H. (F-H) RNA FISH analysis reveals that both α-*nrxn3a* (F, orange) and α*-nrxn3b* (G, cyan) mRNAs are present in lateral-line hair cells. Arrows in F indicate α*-nrxn3a* puncta that are present outside of hair cells, likely in supporting cells. In H, hair cells (*myo6b:memGCaMP6 s*) are shown in grayscale. The dashed lines in F-H outline the location of hair cells. All images are from larvae at 5 dpf. Scale bars: 5 µm (C,F): 1 µm (C, inset).

We used zebrafish to study the role of neurexins in hair-cell synapse maturation. Zebrafish is a relevant model for studying the genetics of hair cells, as many of the core molecules required at hair-cell synapses (e.g. Ca_V_1.3 and Ribeye) are conserved with mammals (Brandt et al., 2003; Jean et al., 2018; Lv et al., 2016; Sidi et al., 2004). In zebrafish, hair cells are present in the inner ear and in the lateral-line system (Fig. 1A-D) – these sensory systems are required for hearing and balance, or the detection of local fluid flow, respectively (Gompel et al., 2001; Haddon and Lewis, 1996). Hair cells in the zebrafish inner ear are innervated by neurons in the statoacoustic ganglion (SAG), whereas hair cells in the lateral-line system are innervated by neurons in the anterior or posterior lateral-line ganglia (aLLg and pLLg) (Zecca et al., 2015).

Our study identifies Nrxn3 as a presynaptic CAM required for hair-cell synapse maturation in both zebrafish and mice. Zebrafish *nrxn3a; nrxn3b* mutants form 60% fewer ribbon synapses in the lateral line and 30-45% fewer ribbon synapses in the inner ear. Loss of Nrxn3 also subtly influences synapse selectivity in lateral-line hair cells that detect anterior flow. Nrxn3 function is also conserved in mammals; in mice, NRXN3 deletion in auditory IHCs results in 20-25% fewer ribbon synapses. Functionally, in zebrafish we find that both pre- and postsynaptic calcium responses in lateral-line hair cells are dramatically reduced in *nrxn3a; nrxn3b* mutants. Despite fewer synapses in mouse and zebrafish hair cells, we observe no detectable deficit in the auditory responses in either species. Overall, Nrxn3 plays a crucial and conserved role in ribbon-synapse maturation in mice and zebrafish. This knowledge will inform future research aimed at rebuilding synapses and restoring hearing or balance after synaptopathy.

## RESULTS

### α-*nrxn3a* and α-*nrxn3b* mRNA are present in hair cells in zebrafish

Neurexins are classic presynaptic CAMs required for synapse assembly in many contexts (Südhof, 2017). In zebrafish, there are two orthologues of mammalian *Nrxn3*, *nrxn3a* and *nrxn3b*, and single-cell RNA sequencing (scRNA-seq) studies have shown that, within the neurexin family (*nrxn1-3*), *nrxn3a* and *nrxn3b* mRNAs are specifically enriched in zebrafish hair cells, making them viable candidates to drive the maturation of ribbon synapses in hair cells (Fig. S1C,D; Tables S1,S2; Lush et al., 2019; Sur et al., 2023).

Like mammalian *Nrxn3*, zebrafish *nrxn3a* and *nrxn3b* loci produce two main isoforms: a long α form and a shorter β form (Fig. 1E) (Gomez et al., 2021). We focused our study on the α form of *nrxn3a* and *nrxn3b*. Using RNA-fluorescence *in situ* hybridization (RNA FISH) we verified that the α-*nrxn3a* and α-*nrxn3b* mRNAs are present in hair cells of the zebrafish lateral line and inner ear (Choi et al., 2018). As neurexins undergo extensive alternative splicing, we designed RNA FISH probes to recognize obligatory exons present in all α form splice variants (Fig. S2). We observed robust and specific α-*nrxn3a* and α-*nrxn3b* RNA FISH signals in hair cells of the lateral line and inner ear (Fig. 1F-H; Figs S3A,B and S4A-I). In addition to hair-cell label, lower levels of α-*nrxn3a* RNA FISH label were detected outside hair cells in glia-like supporting cells (Fig. 1F, see arrows; Fig. S4A,G). Overall, our RNA FISH analysis demonstrates that both α-*nrxn3a* and α-*nrxn3b* mRNAs are present in zebrafish hair cells.

### α-Nrxn3 is required for ribbon-synapse maturation in hair cells in zebrafish

We used zebrafish genetics to test whether α-Nrxn3a or α-Nrxn3b is required for ribbon-synapse maturation at 5 days post fertilization (dpf). At this age, the majority of zebrafish hair cells are mature, and sensory systems are functional (Kimmel et al., 1974; Suli et al., 2012). We obtained α-*nrxn3a* and α-*nrxn3b* zebrafish mutants from the Zebrafish International Resource Center (Kettleborough et al., 2013). Because neurexins undergo extensive alterative splicing, we selected alleles that result in early stop codons in obligatory exons, predicted to disrupt all α-form splice variants (Fig. 1E; Fig. S2).

We first assessed the organization of ribbon synapses in lateral-line hair cells in our α-*nrxn3a* and α-*nrxn3b* mutants using immunohistochemistry to visualize presynapses (pan-CTBP) and postsynapses (pan-MAGUK) (Fig. 1C). We then quantified the number of complete synapses (paired CTBP-MAGUK puncta), unpaired presynapses (lone CTBP puncta) and unpaired postsynapses (lone MAGUK puncta). We found that there was a slight yet significant reduction in the number of complete ribbon synapses per hair cell in α-*nrxn3a* and α-*nrxn3b* single mutants compared with controls (Fig. S5A,B; 18.5% reduction in α-*nrxn3a* mutants and 24% reduction in α-*nrxn3b* mutants). In α-*nrxn3b* mutants*,* but not in α-*nrxn3a* mutants, we observed significantly more unpaired presynapses compared with controls (Fig. S5C). In contrast, we did not observe a difference in the number of unpaired postsynapses in either α-*nrxn3a* or α-*nrxn3b* mutants (Fig. S5D). This assessment suggests that zebrafish hair cells may rely on both α-Nrxn3a and α-Nrxn3b for proper synapse organization.

**Fig. 2.**
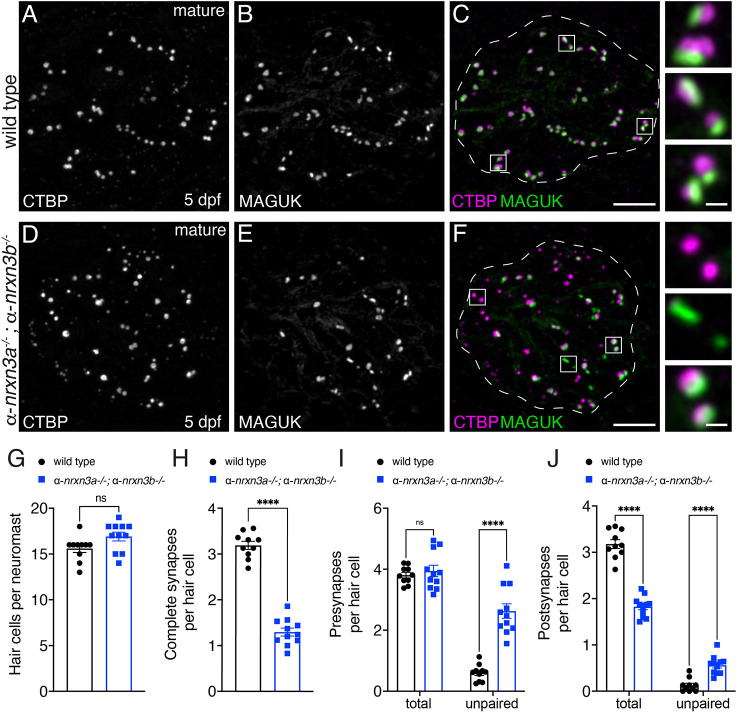
**Loss of α-Nrxn3 impairs synapse organization in mature lateral-line hair cells.** (A-F) Confocal images of mature neuromasts (5 dpf) from wild-type controls (A-C) and α-*nrxn3a;* α-*nrxn3b* mutants (D-F). CTBP labels presynapses (A,D) and MAGUK labels postsynapses (B,E). Merged images are shown in C and F. Dashed lines in C and F outline the hair-cell region [via MYO7A co-label (not shown)]. The boxed areas (magnified on right) in C and F show three examples of individual synapses. (G-J) Quantification shows a similar number of hair cells per neuromast (G), but significantly fewer complete synapses per hair cell in α-*nrxn3a;* α-*nrxn3b* mutants (H). There are more unpaired presynapses (I) and postsynapses (J) per hair cell in α-*nrxn3a;* α-*nrxn3b*. The total number of presynapses remains unchanged (I), whereas the total number of postsynapses (J) per hair cell is decreased in *nrxn3a; nrxn3b* mutants. *n*=10 wild-type and 11 α-*nrxn3a;* α-*nrxn3b* mutant neuromasts at 5 dpf. An unpaired two-tailed *t*-test was used in G and H, and a two-way ANOVA was used in I and J. ns, *P*>0.05; ****P<0.0001. Data are mean±s.e.m. Scale bars: 5 µm (C,F); 0.5 µm (C,F, insets).

Given the modest synaptic defects in each single mutant, we tested whether α-Nrxn3a and α-Nrxn3b have overlapping contributions by examining synapses in α-*nrxn3a;* α-*nrxn3b* double mutants (Fig. 2A-F, 5 dpf). Overall, α-*nrxn3a;* α-*nrxn3b* mutant neuromasts appeared to be morphologically normal (Fig. S6). The number of hair cells per neuromast was unchanged, but the number of supporting cells per neuromast was slightly higher in α-*nrxn3a;* α-*nrxn3b* mutants compared with controls (Fig. S6, Fig. 2G). Importantly, we observed a 60% reduction in the number of complete ribbon synapses per hair cell in α-*nrxn3a;* α-*nrxn3b* mutants compared with controls (Fig. 2A-F,H). We also observed a dramatic increase in the number of unpaired pre- and postsynapses per cell in α-*nrxn3a;* α-*nrxn3b* mutants compared to controls (Fig. 2I,J). This result was especially striking for postsynapses, because we observed an increase in unpaired postsynapses despite a significant decrease in the total number of postsynapses (paired and unpaired) per cell (Fig. 2J). In contrast, despite more unpaired presynapses in α-*nrxn3a;* α-*nrxn3b* mutants, we found that the total number of presynapses per cell was unchanged (Fig. 2I). Overall, this indicates that in mature hair cells of the lateral line, loss of Nrxn3 results in a dramatic decrease in ribbon-synapse numbers and a disruption in pre- and postsynaptic pairing.

We extended our analysis of α-*nrxn3a;* α-*nrxn3b* mutants to hair cells in the zebrafish inner ear. We examined hair cells in the utricle (balance organ) and the medial crista (one of three organs detecting angular acceleration) (example images, Fig. S7A-D). Within both inner-ear epithelia, we found that there were significantly fewer complete synapses per hair cell and significantly more unpaired presynapses and postsynapses in α-*nrxn3a;* α-*nrxn3b* mutants compared with controls (Fig. S8). Together, our analyses indicate that α-Nrxn3 is required for normal ribbon-synapse maturation in hair cells of the zebrafish lateral line and inner ear.

### Zebrafish crispants verify that α-Nrxn3, but not β-Nrxn3, is important for synapse maturation

Despite a significant reduction in ribbon-synapse numbers, complete synapses were still present in α-*nrxn3a*; α-*nrxn3b* mutants. Although our alleles are predicted to eliminate all α-Nrxn3 splice variants (Fig. S2), we could not verify α-Nrxn3 protein loss due to ineffective antibodies. RNA FISH quantification showed a partial reduction in α-*nrxn3a* and α-*nrxn3b* mRNA puncta per hair cell in α-*nrxn3a*; α-*nrxn3b* mutants (α-*nrxn3a*: 31%; α-*nrxn3b*: 18%, Fig. S9A-E). This indicates effective germline lesioning and that some mRNAs are undergoing nonsense-mediated decay (El-Brolosy et al., 2019).

Owing to the persistence of α-*nrxn3a* and α-*nrxn3b* mRNAs, we used Crispr-Cas9 for further verification. We designed gRNAs targeting similar exons of α-*nrxn3a* and α-*nrxn3b* (Fig. 1E; Figs S2 and S10A), and injected gRNAs along with Cas9 protein into single-cell embryos to create F0 crispants. Synaptic phenotypes in mature hair cells were assessed in F0 crispants at 5 dpf. This analysis revealed that that α-*nrxn3a*; α-*nrxn3b* crispants had 40% fewer complete synapses and more unpaired pre- and postsynapses compared with controls (Fig. S10B-K). These results indicate that α-*nrxn3a*; α-*nrxn3b* crispants phenocopy our germline mutants.

After verifying the efficacy of using F0 crispants, we also created F0 β-*nrxn3a* and β-*nrxn3b* crispants (Fig. S10A). We created F0 β-*nrxn3a*; β-*nrxn3b* double crispants to test whether the β isoform could also play a role in synapse maturation. We found that β-*nrxn3a*; β-*nrxn3b* crispants had a similar number of synapses as controls, indicating that β-Nrxn3a and β-Nrxn3b are not essential for ribbon-synapse maturation (Fig. S10L-O).

Overall, both our germline and crispant mutants indicate that α-Nrxn3a and α-Nrxn3b are essential for pre- and postsynaptic pairing and ribbon-synapse maturation in mature hair cells of the lateral line. In contrast, β-Nrxn3a and β-Nrxn3b do not play an essential role in ribbon-synapse maturation. Moving forward, we focused our zebrafish work on α-*nrxn3a;* α-*nrxn3b* germline mutants. For simplicity, we use Nrxn3a and Nrxn3b from here on to refer to the long α form of each Nrxn3 orthologue, unless otherwise specified.

### Nrxn3 is required early in zebrafish hair-cell synapse maturation

Our results show that loss of Nrxn3 can dramatically impact the pairing of synaptic components in hair cells (Fig. 2; Figs S7 and S8). Initially, we examined zebrafish hair cells at 5 dpf, when most hair cells are mature. Because hair cells are already mature at this stage, it is difficult to determine whether Nrxn3 is required early for synapse assembly, or later for maintenance or refinement. To address this, we examined hair cells in zebrafish at 3 dpf, when hair cells are still developing and sensory systems are not yet functional.

**Fig. 3.**
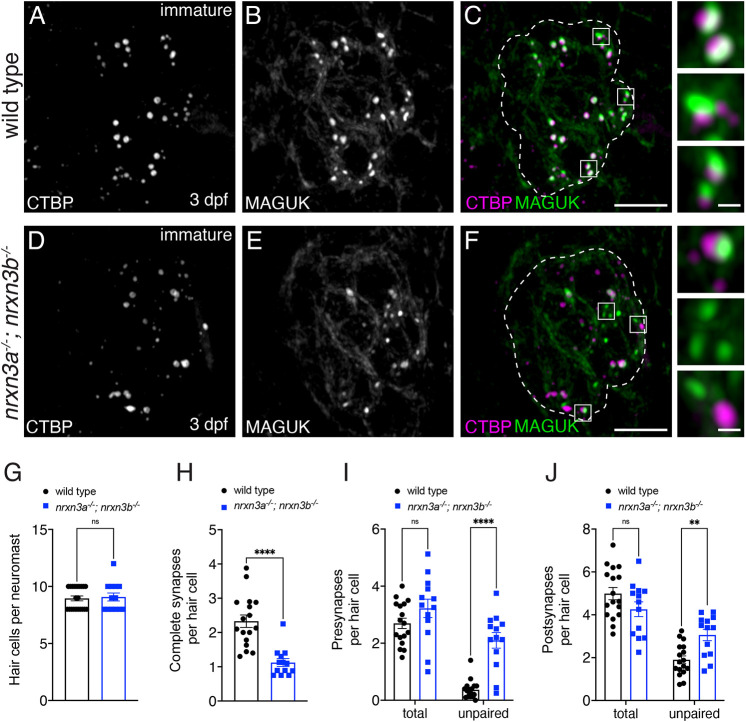
**Nrxn3 is required for early synapse maturation in lateral-line hair cells.** (A-F) Confocal images of developing hair cells (3 dpf) from wild-type controls (A-C) and *nrxn3a; nrxn3b* mutants (D-F). CTBP labels presynapses (A,D) and MAGUK labels postsynapses (B,E). Merged images are shown in C and F. The boxed areas (magnified on right) in C and F show three examples of individual synapses. Dashed lines in C and F outline the hair-cell region [via a MYO7A co-label (not shown)]. (G-J) Quantification shows a similar number of hair cells per neuromast (G), but significantly fewer complete synapses per hair cell in *nrxn3a; nrxn3b* mutants (H). There are significantly more unpaired presynapses (I) and postsynapses (J) per hair cell in *nrxn3a; nrxn3b* mutants. In developing hair cells, there is no change in the total number of presynapses (I) or postsynapses (J) per hair cell in *nrxn3a; nrxn3b* mutants. *n*=17 wild-type and 13 *nrxn3a; nrxn3b* mutant neuromasts. An unpaired two-tailed *t*-test was used in G and H, and a two-way ANOVA was used in I and J. ns, *P*>0.05; **P<0.01; ****P<0.0001. Data are mean±s.e.m. Scale bars: 5 µm (C,F); 0.5 µm (C,F, insets).

Using immunostaining, we quantified the number of complete synapses and unpaired pre- and postsynapses in developing hair cells at 3 dpf in *nrxn3a; nrxn3b* mutants (example images, Fig. 3A-F). We found that developing hair cells in *nrxn3a; nrxn3b* mutants showed a significant, 35% reduction in the number of complete ribbon synapses per hair cell compared with controls (Fig. 3G,H). This reduction is less dramatic than the 60% reduction observed in mature hair cells (Fig. 2). In developing hair cells, we also observed a dramatic increase in the number of unpaired presynapses (Fig. 3I) and a more modest increase in unpaired postsynapses (Fig. 3J). In contrast to mature hair cells at 5 dpf, in developing hair cells we did not observe a reduction in the total number of postsynapses per hair cell (Fig. 3J). The presence of more complete synapses and a normal number of postsynapses at 3 dpf versus 5 dpf in *nrxn3a; nrxn3b* mutants suggests that, although synapses initially form correctly, they may ultimately fall apart, leading to a loss of postsynapses. Overall, Nrxn3 appears to play a role early in synapse maturation.

### Nrxn3 plays a subtle role in afferent selectivity within the zebrafish lateral line

**Fig. 4.**
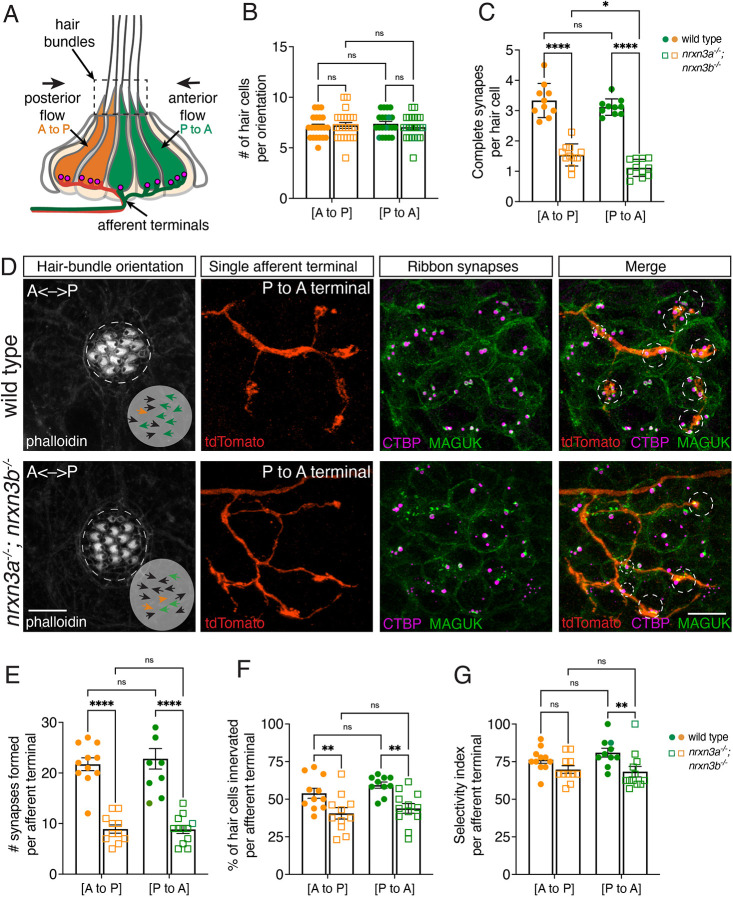
**Nrxn3 impacts synapse loss and selectivity to a greater extent in hair cells sensing anterior flow.** (A) Primary pLL neuromasts have two populations of hair cells: one responds to anterior flow (green, P to A) and the other responds to posterior flow (orange, A to P). Each population is selectively innervated by distinct afferent neurons (green and orange terminals). (B) Phalloidin label reveals similar numbers of hair cells per neuromast oriented P-to-A and A-to-P flow in wild type and *nrxn3a; nrxn3b* mutants. *n*=21 wild-type and 23 *nrxn3a; nrxn3b* mutant neuromasts at 5 dpf. (C) There are fewer complete synapses in hair cells responding to P-to-A and A-to-P flow in *nrxn3a; nrxn3b* mutants. There is also a significant reduction in complete synapses in hair cells responding to P to A flow compared with those responding to A to P flow in *nrxn3a; nrxn3b* mutants. *n*=10 wild-type and 11 *nrxn3a; nrxn3b* mutant neuromasts at 5 dpf. (D) Single afferent terminal labeling (P to A fibers) in wild type (top) and an *nrxn3a; nrxn3b* mutant (bottom). Phalloidin labels hair bundles (orientation indicated by arrows); tdTomato labels individual afferent terminals; CTBP labels presynapses; MAGUK labels postsynapses, and faintly labels hair-cell outlines to provide context. The green and orange arrows in the Phalloidin images indicate the orientation of hair cells that are correctly or incorrectly innervated by each terminal, respectively. The black arrows indicate hair cells that are not innervated by the terminal. In the merged images, large and small circles indicate the synapses and cells that are correctly or incorrectly innervated by each terminal, respectively. (E-G) Quantification shows reduced hair-cell innervation (F) and synapses (E) in both P to A and A to P terminals in *nrxn3a; nrxn3b* mutants compared with controls. The selectivity of P to A terminals but not A to P fibers is reduced in *nrxn3a; nrxn3b* mutants compared with controls (G). A two-way ANOVA was used in B,C,E-G. ns, *P*>0.05; *P<0.05; **P<0.01; ****P<0.0001. Data are mean±s.e.m. Scale bars: 5 µm.

In lateral-line neuromasts, hair cells are oriented to detect fluid flow in two opposing directions – in the primary neuromasts of the pLL, flow towards the anterior (P-to-A) and towards the posterior (A-to-P) of the animal (see Fig. 4A). Approximately four to six afferent neurons selectively innervate neuromasts based on hair-cell orientation (Haehnel et al., 2012; Nagiel et al., 2008). Because neurexins are implicated in synapse specification (Gomez et al., 2021; Südhof, 2017), we examined the afferent innervation patterns in *nrxn3a; nrxn3b* mutants.

We first examined whether the synaptic defects in *nrxn3a; nrxn3b* mutants were linked to a particular hair-cell orientation. For our analysis, we used immunohistochemistry to label presynapses, postsynapses and hair-cell orientation (Phalloidin label). This labeling revealed that, similar to controls, *nrxn3a; nrxn3b* mutants had a similar number of A-to-P and P-to-A hair cells per neuromast (Fig. 4B). However, there was a dramatic reduction in the number of complete synapses in both hair-cell orientations in *nrxn3a; nrxn3b* mutants (Fig. 4C). Notably, *nrxn3a; nrxn3b* mutants had significantly fewer complete synapses in hair cells detecting anterior (P-to-A) flow compared with posterior (A-to-P) flow (Fig. 4C; complete synapses: A-to-P: 1.54; P-to-A: 1.14; *P*=0.014). These results suggest that, although Nrxn3 is crucial for ribbon-synapse maturation in both hair-cell orientations, it may be even more important for synapse maturation in hair cells that detect anterior (P-to-A) flow.

Although loss of Nrxn3 reduced synapse pairing in hair cells of both orientations, it was still unclear whether individual afferent neurons still selectively innervated hair cells based on their orientation or how many afferent neurons innervated each neuromast. Therefore, we examined the afferent neurons more closely in *nrxn3a; nrxn3b* mutants. Using a transgenic line that labels the pLL neurons (*en.sill,hsp70l:GCaMP6s*) we found no difference in the number of cell bodies per ganglia between *nrxn3a; nrxn3b* mutants and controls (Fig. S11A). In addition, we used immunohistochemistry to assess whether there were gross differences in the area occupied by the four to six afferent terminals beneath lateral-line hair cells (Calretinin label; Fig. S11B-D). We found that the average area occupied by the afferent terminals was not significantly different between *nrxn3a; nrxn3b* mutants and controls (Fig. S11B-D). Unfortunately, we were unable to resolve the total number of afferent terminals contacting each neuromast using light microscopy. But overall, this assessment indicates that there is no gross loss of afferent neurons or terminal area after synapse loss in *nrxn3a; nrxn3b* mutants.

We next examined the selectivity of individual afferent terminals in *nrxn3a; nrxn3b* mutants more closely. To label individual neurons, we microinjected a *neurod:tdTomato* construct at the single-cell stage. To assess innervation selectivity, we immunostained zebrafish expressing tdTomato to visualize individual afferent terminals, synapses and hair-cell orientation (example images, Fig. 4D). Using this approach, we evaluated several features of A-to-P and P-to-A afferent terminal types, including: percent of hair cells innervated neuromast, number of complete synapses formed and innervation selectively based on orientation. In *nrxn3a; nrxn3b* mutants, we found that both the number of cells innervated and the number of complete synapses formed were similarly reduced in each terminal type (Fig. 4E,F). We then examined how selective the afferent terminals were for their preferred orientation (Selectivity index=number preferred hair cells innervated/total number of hair cells innervated×100). We found that the afferent terminals that contact P-to-A hair cells were slightly less selective in *nrxn3a; nrxn3b* mutants compared with controls (Fig. 4G; Selectivity index, control: 80.9; *nrxn3a; nrxn3b*: 68.4; *P*=0.004). In contrast, we observed no change in the selectivity of afferent terminals that contact A-to-P hair cells (Fig. 4G; Selectivity index, control: 76.3; *nrxn3a; nrxn3b*: 69.8; *P*=0.120).

Overall, our analysis revealed that loss of Nrxn3 led to a significant reduction in both the number of innervated hair cells and the number of synapses formed by individual afferents. These reductions were observed in afferents that selectively innervate hair cells of both orientations (A-to-P and P-to-A). However, the loss of Nrxn3 led to an even greater reduction in synapses in P-to-A hair cells compared with A-to-P hair cells, and the afferents forming these synapses exhibited decreased selectivity.

### Nrxn3 alters pre- and postsynapse size and Ca_V_1.3 channel distribution in zebrafish

Studies in the central nervous system have shown that loss of neurexins can alter synapse morphology and clustering of synaptic components (Brockhaus et al., 2018; Luo et al., 2020; Uemura et al., 2022). We examined the morphology of synaptic components, including presynapses, postsynapses and Ca_V_1.3 channels, in *nrxn3a; nrxn3b* mutants at 5 dpf. We examined maximum intensity projections and quantified the 2D area and average intensity of these synaptic components. For our analysis, we examined the size of paired and unpaired synaptic components separately.

**Fig. 5.**
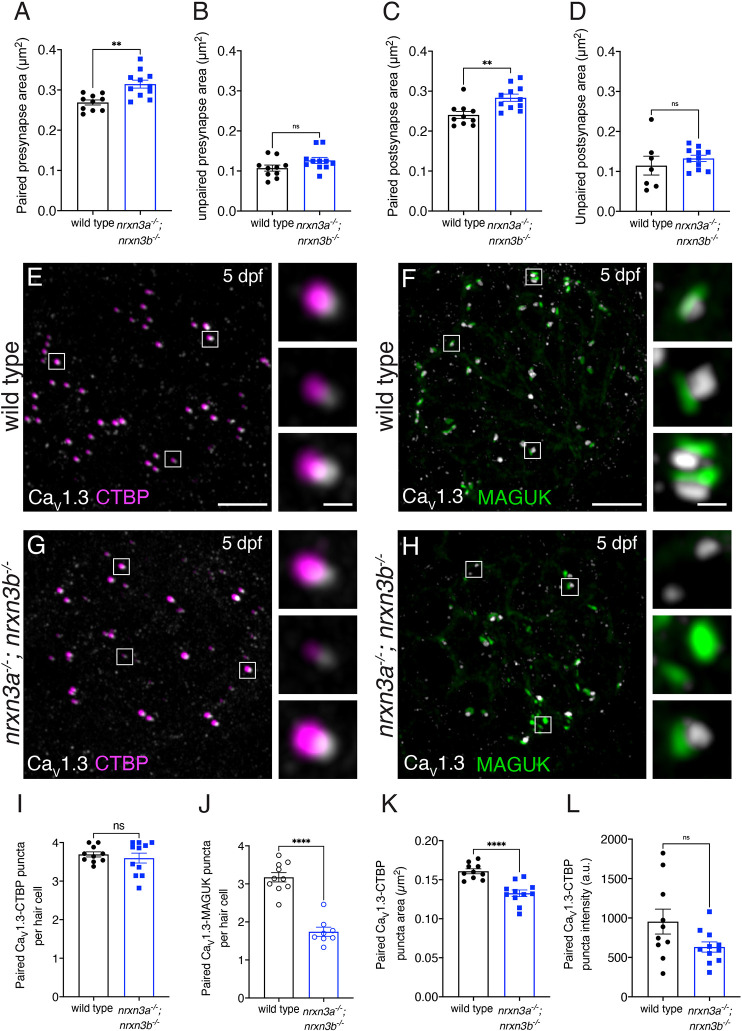
**Loss of Nrxn3 impacts pre- and postsynapse size and Ca_V_1.3 channel localization in lateral-line hair cells.** (A-D) There is a significant increase in the area of paired (A,C) but not unpaired (B,D) pre- and postsynapses in *nrxn3a; nrxn3b* mutants compared with wild-type controls. (E-H) Confocal images of mature neuromasts from wild-type controls (E,F) and *nrxn3a; nrxn3b* mutants (G,H). CTBP labels presynapses with Ca_V_1.3 (E,G) and MAGUK labels postsynapses with Ca_V_1.3 (F,H). The boxed areas (magnified on right) show three examples of individual synapses. Hair cells in A-D were visualized with an Otoferlin or Parvalbumin co-label (not shown). (I-L) Quantification shows no change in the number of Ca_V_1.3-CTBP paired puncta per hair cell in *nrxn3a; nrxn3b* mutants (I). However, there are significantly fewer Ca_V_1.3-MAGUK paired puncta per hair cell in *nrxn3a; nrxn3b* mutants (J). The area (K) but not the average intensity (L) of Ca_V_1.3 puncta associated with CTBP puncta is significantly lower in *nrxn3a; nrxn3b* mutants. *n*=10 wild-type and 11 *nrxn3a; nrxn3b* mutant neuromasts in A-D,I,K,L and *n*=10 wild-type and 8 *nrxn3a; nrxn3b* mutant neuromasts in J. Images and quantification are from larvae at 5 dpf. An unpaired two-tailed *t*-test was used in A-D and I-L. ns, *P*>0.05; **P<0.01; ****P<0.0001. Data are mean±s.e.m. Scale bars: 5 µm (E-H); 1 µm (E-H, insets).

We first examined how loss of Nrxn3 impacts the average area (size) of presynaptic (CTBP) and postsynaptic (MAGUK) puncta. We found that the average size of paired presynapses (paired CTBP-MAGUK puncta) was significantly increased in *nrxn3a; nrxn3b* mutants, whereas unpaired presynapses were similar to controls (Fig. 5A,B). Similarly, we found that the average size of paired postsynapses (paired CTBP-MAGUK puncta) was significantly increased in *nrxn3a; nrxn3b* mutants, whereas unpaired postsynapses were a similar size compared with controls (Fig. 5C,D). This suggests that synapses in *nrxn3a*; *nrxn3b* mutants do not properly coalesce, even when paired.

Previous work has shown that in hair cells, presynaptic Ca_V_1.3 channel distribution is shaped by presynapse size (Sheets et al., 2017). Because presynapse area and number were altered in *nrxn3a; nrxn3b* mutants, we examined Ca_V_1.3 channel distribution via immunohistochemistry (example images, Fig. 5E-H). We found that the number of CTBP-Ca_V_1.3 paired puncta per hair cell was similar in *nrxn3a; nrxn3b* mutants compared with controls (Fig. 5I). In contrast, we found a reduced number of MAGUK-Ca_V_1.3 paired puncta per hair cell in *nrxn3a; nrxn3b* double mutants compared with controls (Fig. 5J). This latter reduction mirrors the reduction in complete synapses in *nrxn3a; nrxn3b* mutants (Fig. 2H). Our examination of Ca_V_1.3 pairing indicates that Nrxn3 is not required for presynapses to couple with Ca_V_1.3 channels. Instead, Nrxn3 may be required to pair a presynapse and its associated Ca_V_1.3 channels with an adjacent postsynapse.

Lastly, we examined the size and distribution of Ca_V_1.3 channels within each CTBP-Ca_V_1.3 paired puncta. Though the area of Ca_V_1.3 puncta was significantly reduced in *nrxn3a; nrxn3b* double mutants, the average intensity of each Ca_V_1.3 punctum was unchanged compared with controls (Fig. 5K,L). This indicates that, on average, fewer Ca_V_1.3 channels may reside within each Ca_V_1.3 puncta in *nrxn3a; nrxn3b* mutants compared with controls. Together, this analysis of synapse morphology demonstrates that Nrxn3 is important to ensure synapses form with the proper amount of Ca_V_1.3 channels and to form pre- and postsynapses of the correct size.

### Nrxn3 plays a conserved role in inner hair cell ribbon-synapse maturation in mice

Many of the core genes required at hair-cell synapses are conserved between zebrafish and mammals (Sheets et al., 2021). scRNA-seq studies in mouse auditory (cochlea) and vestibular (utricle) organs have shown that both *Nrxn3* and *Nrxn2* are expressed in hair cells (Fig. S1A,B; Tables S3,S4; Cai et al., 2015; Elkon et al., 2015; Kolla et al., 2020). However, whether either NRXN2 or NRXN3 play a role in mammalian hair-cell synapse maturation has not yet been demonstrated.

**Fig. 6.**
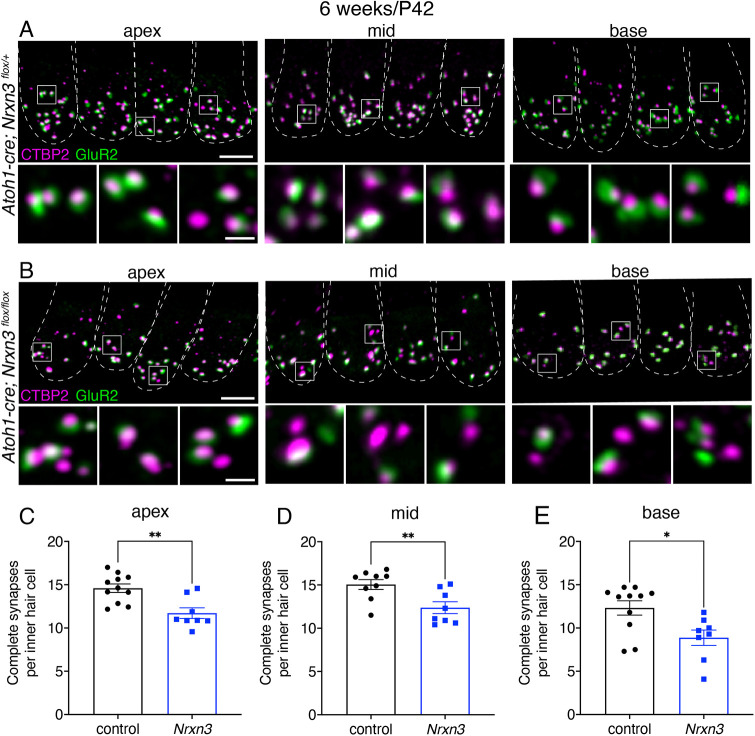
**NRXN3 is required at 6 weeks for proper synapse number in mouse auditory inner hair cells.** (A,B) Confocal images of mouse inner hair cells (IHCs) at 6 weeks (P42) from control (A) and *Nrxn3* mutant animals (*Atoh1-Cre; Nrxn3^flox/flox^*) (B). CTBP2 labels the presynapses and GluR2 labels the postsynapses. Merged images show four IHCs from three different regions of the cochlea (apex, middle and basal thirds) for each genotype. Dashed lines outline hair-cell bodies [via a MYO7A co-label (not shown)]. White boxes in the top panels are magnified below to highlight synapses more clearly. (C-E) Quantification reveals that, compared with controls, *Nrxn3* mutants have significantly fewer complete synapses per IHC at the apex (C), mid (D) and base (E). These findings were compiled from four animals from each genotype and from two independent litters and immunostains. Each dot represents the average synapse number from one imaging region (6-9 IHCs). Two imaging regions were examined per animal for each tonotopic region. An unpaired two-tailed *t*-test was used in C-E. *P<0.05; **P<0.01. Data are mean±s.e.m. Scale bar: 5 µm (A,B); 1 µm (A,B, insets).

To extend our analysis of Nrxn3 to mice, we used a conditional inactivation strategy. We bred the *Nrxn3^flox^* strain (Aoto et al., 2015) with the well-established *Atoh1-Cre* driver (Matei et al., 2005) to delete exon 18 and abrogate both the α- and β-isoforms of *Nrxn3* in postmitotic hair cells (Fig. S12A). Using PCR, we were able to confirm that the *Atoh1-Cre* driver was able to excise exon 18 in the mouse cochlea (Fig. S12B). We examined ribbon synapses in auditory IHCs of *Atoh1-Cre; Nrxn3^flox/flox^* mutants (*Nrxn3* mutants) and control animals by immunolabeling both presynapses (CTBP2) and postsynapses (GluR2). We examined IHCs at 4 (postnatal day 28) and 6 (postnatal day 42) weeks of age. At both ages we found a significant reduction (20-25%) in the number of complete synapses (paired CTBP2-GluR2 puncta) per IHC across all tonotopic regions of the mouse cochlea (cochlear apex, mid and basal thirds) in *Nrxn3* mutants compared with controls (Fig. 6A-E; Fig. S13A-E). These results indicate that NRXN3 plays a conserved role in ribbon-synapse maturation in mouse IHCs.

### Nrxn3 disrupts ribbon-synapse function in zebrafish

**Fig. 7.**
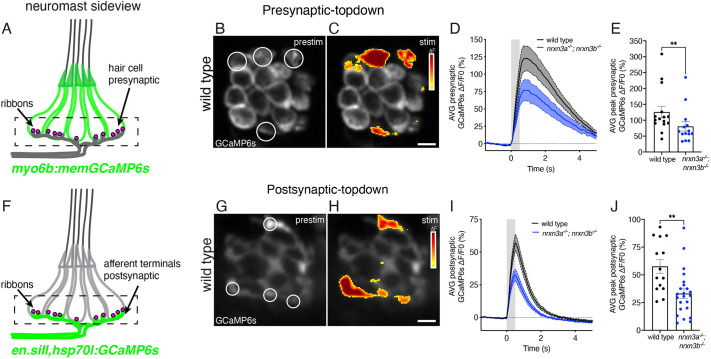
**Nrxn3 is required for proper hair-cell synapse function in the lateral line.** (A) Side view schematic of a neuromast expressing memGCaMP6s in hair cells. The dashed box indicates the region used to measure presynaptic GCaMP6s responses. (B,C) ΔF heatmaps showing presynaptic GCaMP6s increases in hair cells before (B) and during (C) a 500 ms fluid-jet stimulation in a wild-type neuromast. ROIs (circled) indicate synaptically active hair cells. The black area in the center of each cell is the nucleus. (D) ΔF/F0 GCaMP6s traces showing average presynaptic responses during stimulation for wild-type controls (black) and *nrxn3a; nrxn3b* mutants (blue). (E) Maximum ΔF/F0 presynaptic calcium responses are significantly reduced in *nrxn3a; nrxn3b* mutants. *n*=15 wild-type and 14 *nrxn3a; nrxn3b* mutant neuromasts at 5-6 dpf. (F) Side view schematic of a neuromast expressing GCaMP6s in afferent terminals beneath hair cells. The dashed box indicates the region used to measure postsynaptic GCaMP6s responses. (G,H) ΔF heatmaps showing postsynaptic GCaMP6s increases in afferent terminals before (G) and during (H) a 500 ms fluid-jet stimulation in a wild-type neuromast. ROIs (circled) indicate synaptically active terminals. (I) ΔF/F0 GCaMP6s traces showing average postsynaptic responses during stimulation for wild-type controls (black) and *nrxn3a; nrxn3b* mutants (blue). (J) Maximum ΔF/F0 postsynaptic calcium responses to stimulation for wild-type controls and *nrxn3a; nrxn3b* mutants. *n*=13 wild-type and 22 *nrxn3a; nrxn3b* mutant neuromasts at 4-5 dpf. Traces in D and I are displayed as mean, dashed lines are s.e.m.; gray bar denotes the stimulus. Each dot in E and J represents the average response from a single neuromast. A Mann–Whitney *U*-test was used in E and an unpaired two-tailed *t*-test was used in J. **P<0.01. Data are mean±s.e.m. Scale bars: 5 µm.

After verifying that Nrxn3 is essential for proper synapse maturation, we assessed the functional impact of synapse loss. Using previously established approaches, we used transgenic fish expressing GCaMP6s in hair cells (*myo6b:memGCaMP6s*) or afferent neurons (*en.sill,hsp70l:GCaMP6s*) to measure evoked and baseline calcium signals in the lateral-line system (Fig. 7; Fig. S14; Zhang et al., 2018).

Before taking our evoked calcium measurements, we first examined whether there were differences in resting GCaMP6s levels in *nrxn3a; nrxn3b* mutants at baseline. We observed no difference in the baseline or resting GCaMP6s signal in hair bundles, presynapses or postsynaptic terminals (Fig. S14A-C). After our baseline measurements, we assessed evoked GCaMP6s responses using a fluid jet to stimulate hair cells. We first tested whether *nrxn3a; nrxn3b* mutants have normal mechanotransduction (e.g. normal ability of hair cells to detect sensory stimuli). We found that the magnitude of GCaMP6s signals measured in hair bundles was not significantly different in *nrxn3a; nrxn3b* mutants compared with controls (Fig. S14D-F). In contrast, when we assessed the magnitude of evoked GCaMP6s signals measured in the presynaptic region of hair cells, we found that GCaMP6s signals were reduced by 35% in *nrxn3a; nrxn3b* mutants compared with controls (Fig. 7A-E; Fig. S15A-F). These signals were measured at the level of individual hair cells, not individual synapses. Thus, a reduction in presynaptic calcium signals could be due to fewer complete synapses, smaller presynapses or altered calcium channel density.

We next examined evoked GCaMP6s signals in the afferent terminals of *nrxn3a; nrxn3b* mutants (Fig. 7F-J). We observed a 45% reduction in the magnitude of evoked GCaMP6s signals in the terminals of *nrxn3a; nrxn3b* mutants compared with controls (Fig. 7G-J; Fig. S15G-L). Despite reduced postsynaptic responses, the total number of active postsynaptic sites per neuromast was the same in *nrxn3a; nrxn3b* mutants and controls (Fig. S16A). However, when we quantified the number of active postsynaptic sites per stimulus direction, A to P versus P to A, we observed significantly fewer active postsynaptic sites in the P-to-A direction compared with the A-to-P direction in *nrxn3a; nrxn3b* mutants compared with controls (Fig. S16B). Together, our calcium imaging experiments indicate that the synapse loss in *nrxn3a; nrxn3b* mutants leads to a dramatic reduction in pre- and postsynaptic response magnitudes. In addition, *nrxn3a; nrxn3b* mutants have fewer active postsynaptic sites, specifically in response to P-to-A directed stimuli.

### Nrxn3 loss does not disrupt auditory responses in mice or zebrafish

Our immunohistochemistry results in zebrafish and mice indicate that loss of Nrxn3 results in fewer synapses. Calcium imaging in zebrafish shows that ribbon-synapse function is significantly reduced. To determine whether loss of Nrxn3 also impacts hair cell-mediated behaviors, we assessed auditory function in *Nrxn3* mutants. In mice, we measured auditory brainstem responses (ABR), which reflect the electrical responses of spiral ganglion neurons (SGNs) and central nervous system nuclei during sound stimulation (Zhou et al., 2006). In zebrafish, we used the acoustic startle response, which relies on hair cells in the inner ear and lateral line, to assess hair-cell function.

**Fig. 8.**
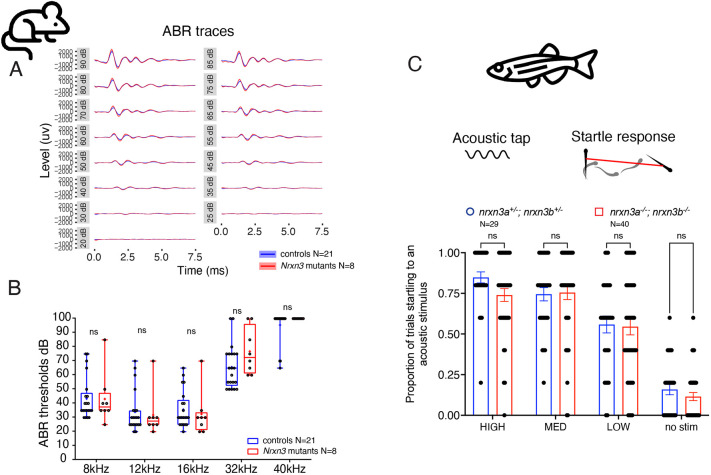
**Auditory behaviors are unaltered in mouse and zebrafish after loss of Nrxn3.** (A) Average auditory brainstem responses (ABRs) from control and *Nrxn3* mutant animals (*Atoh1-Cre; Nrxn3^flox/flox^*) at P28-P32 from 20 to 90 dB are shown. (B) No difference was observed between control and *Nrxn3* mutants with regards to the ABR threshold at any frequency tested. *n*=21 control and 8 *Nrxn3* mutants. Distributions are framed with 25-75% whisker boxes where exterior lines show the minimum and maximum, the middle line represents the median, and + represents the mean. (C) A vibrational acoustic tap stimulus was used at three stimuli of decreasing intensity to trigger an escape response in *nrxn3a^+/−^; nrxn3b^+/−^* double heterozygotes and *nrxn3a^−/−^; nrxn3b^−/−^* double mutants. The proportion of times (out of five trials) an animal responded to each stimulus is shown. No difference was observed at any stimulus intensity. *n*=29 *nrxn3a; nrxn3b* double heterozygotes and 40 *nrxn3a; nrxn3b* double mutants at 5 dpf. Data are mean±s.e.m. A two-way ANOVA was used in B and C. ns, *P*>0.05.

We tested *Nrxn3* mutant mice at postnatal days 28-32 and found that ABR waveforms and thresholds (minimum auditory stimuli needed to evoke an ABR response above the noise floor) were similar compared with controls at all frequencies tested (Fig. 8A,B). In mice, an ABR waveform is represented by five defined waves (I-V). In particular, wave I represents the response of the SGNs, and wave I amplitude has been correlated with IHC synapse numbers (Kujawa and Liberman, 2009; Sergeyenko et al., 2013). We found that the magnitude and latency of the wave I response were not different between *Nrxn3* mutants and control animals (Fig. S17A,B). This indicates that the synapse loss in *Nrxn3* mutants does not impair ABR responses.

We next measured acoustic startle responses in larval zebrafish. For an in-clutch comparison, we compared *nrxn3a; nrxn3b* double mutants with *nrxn3a^+/−^; nrxn3b^+/−^* double heterozygotes. We assayed the acoustic startle response at three different intensities. Using this approach, we observed no difference in the proportion of animals startling at any stimulus intensity between the two genotypes (Fig. 8C). Overall, our behavioral results suggest that the reduced number of synapses observed with loss of Nrxn3 is not sufficient to disrupt ABR in mice or acoustic startle responses in zebrafish.

## DISCUSSION

In zebrafish and mice, we find a dramatic reduction in the number of ribbon synapses in hair cells when α-Nrxn3 is absent [mouse *Nrxn3* conditional knockout (cKO) and *nrxn3a; nrxn3b* zebrafish mutants]. In zebrafish, early development shows normal numbers of pre- and postsynapses in *nrxn3a; nrxn3b* mutants, but later development reveals a failure to pair, leading to a loss of postsynapses. Additionally, in the zebrafish lateral line, afferents contacting hair cells that respond to anterior flow are less selective in *nrxn3a; nrxn3b* mutants. Lastly, we demonstrate that fewer synapses in *nrxn3a*; *nrxn3b* mutants result in a reduction in the magnitude of both pre- and postsynaptic calcium responses. Overall, this work highlights a conserved role for Nrxn3 in the maturation of ribbon synapses in hair cells.

### Postsynaptic partners for Nrxn3 at the ribbon synapse in hair cells

Our work demonstrates that Nrxn3 is required for the proper maturation of ribbon synapses in zebrafish and mouse hair cells. Neurexins serve as presynaptic receptors for several extracellular binding partners, including secreted cerebellins and neuroexophilins, as well as transmembrane proteins such as neuroligins, Dystroglycan, leucine-rich repeat transmembrane proteins (LRRTM) and Calsyntenin-3 (Boucard et al., 2005; Dai et al., 2022; Hauser et al., 2022; Kim et al., 2020; Ko et al., 2009; Sugita et al., 2001; Trotter et al., 2023). Recent work has shown that the α-neurexin binding partners NLGN1 and NLGN3 are present at mouse auditory IHC postsynapses (Ramirez et al., 2022). Loss of NLGN1 and NLGN3 results in a 25% reduction in ribbon synapses in IHCs (Ramirez et al., 2022). Similarly, our work shows a 20-25% reduction in ribbon synapses in *Nrxn3* mouse mutants (Fig. 6; Fig. S13), suggesting that α-Nrxn3 may couple with NLGN1 and NLGN3 at auditory IHC synapses in mice. In the future, it will be interesting to examine zebrafish lacking NLGN1 and NLGN3 to determine whether this complex plays a conserved role in vertebrates.

In mice lacking NLGN1 and NLGN3 or NRXN3, and zebrafish lacking α-Nrxn3, there is a reduction in synapse numbers, but some intact synapses remain. Our zebrafish studies indicate that β-Nrxn3 does not impact synapse maturation (Fig. S10), suggesting that another CAM is involved in synapse maturation. Additionally, scRNA-seq data show that mouse auditory and vestibular hair cells express *Nrxn3* as well as *Nrxn2* (Fig. S1). Whether another CAM or neurexin plays a role in synapse maturation in hair cells remains to be determined.

### A role for Nrxn3 in hair-cell synapse specificity?

If Nrxn3 and Nlgn1/3 are not required to mature all ribbon synapses in mice or zebrafish, they may only be required in a specific subset of synapses. One example of subset specificity in the lateral line is the selective innervation of hair cells based on the direction of flow they sense (A-to-P or P-to-A) (Nagiel et al., 2008). In our work, we observed that *nrxn3a; nrxn3b* mutants had fewer synapses in both hair-cell populations (Fig. 4C). But we also observed a notably greater loss of synapses in hair cells that sense anterior fluid flow (P to A, Fig. 4C). Further, we found that afferent neurons innervating P-to-A hair cells were less selective without Nrxn3 (Fig. 4G). Lastly, we also observed that loss of Nrxn3 resulted in fewer active postsynaptic sites, specifically in response to P-to-A directed flow (Fig. S16). Together, these results indicate that, mechanistically, α-Nrxn3 may play a larger role in synapse maturation in hair cells that sense anterior flow.

Another level of innervation specificity in the lateral line is reflected in the wiring pattern each neuron makes within the afferent terminals beneath neuromast hair cells. In the lateral line, hair cells are redundantly innervated by multiple neurons (Haehnel et al., 2012; Nagiel et al., 2008). Connectomic work using serial block-face scanning electron microscopy (SBF-SEM) has shown that each neuromast is primarily innervated by a dominant afferent neuron that forms synapses with nearly all hair cells of the same orientation, accounting for 75% of the synapses (Dow et al., 2018). Additionally, one to three other afferent neurons form the remaining synapses within the neuromast. These anatomical differences suggest there may be unique subtypes of neurons in the posterior-lateral line system (Dow et al., 2018). However, there are no molecular markers for these subtypes, and currently they can only be resolved morphologically using serial electron microscopy. Consequently, we could not determine the number of neurons innervating each neuromast in *nrxn3a; nrxn3b* mutants or the role of Nrxn3 in these afferent neuron subtypes. In the future, it will be important to identify subtype-specific markers or use SBF-SEM to investigate the role Nrxn3 plays in the lateral line with regards to these potential neuronal subtypes.

Although neuronal subtypes are less defined in the lateral line, scRNA-seq studies in the mouse auditory system have demonstrated that there are at least three main subtypes of afferent neurons (Type I spiral ganglion neurons) that innervate auditory IHCs (Shrestha et al., 2018; Sun et al., 2018). Each subtype synapses onto hair cells at roughly distinct spatial locations (across the pillar and modiolar faces of the cell) and loosely corresponds to a distinct functional profile (Liberman et al., 2011; Petitpré et al., 2018; Taberner and Liberman, 2005). Work on NLGN1 and NLGN3 in mice has shown that NLGN1 localizes to postsynapses contacting the modiolar face of the auditory IHCs (Ramirez et al., 2022). In contrast, the pillar face of auditory IHCs is populated by postsynapses containing NLGN3 or NLGN1/3. Whether NRXN3 or NLGN1/3 are required for synapse maturation in a specific neuronal subtype in the mouse auditory system remains to be determined.

### Nrxn3 interactions with core presynaptic components

Despite being classic synaptic adhesion molecules, loss of neurexins does not lead to synapse loss in all contexts. For example, in *α-Nrxn1/2/3* triple KO mice, no loss of glutamatergic synapses was observed in the brainstem (Missler et al., 2003) and calyx of Held synapses were intact (Luo et al., 2020). Instead, both studies found that α-NRXNs are important for calcium channel clustering. These clustering defects impair neurotransmission, despite normal synapse numbers. These data led to the hypothesis that α-Nrxns may act to couple calcium channels to presynaptic machinery.

In zebrafish hair cells, we found that loss of Nrxn3 results in smaller Ca_V_1.3 clusters (pore-forming α subunit), along with disrupted neurotransmission (Figs 5E-H,K and 7). In hippocampal neurons, α-NRXNs can regulate neurotransmission via interactions with α2δ1 auxiliary subunits of calcium channels (Brockhaus et al., 2018). Based on this work, it is possible that the synaptic defects in *nrxn3a; nrxn3b* mutants may stem from disrupted interactions between Nrxn3 and an α2δ subunit of Ca_V_1.3 channels. Consistent with this idea, work in mouse auditory IHCs has shown that α2δ2 subunits are required to properly gate Ca_V_1.3 channels as well as to align these channels with the postsynapse (Fell et al., 2016). In future work, it will be interesting to pursue both the physical and functional link between Nrxn3 and all subunits of Ca_V_1.3 channels.

To further understand Nrxn3 interactions, visualizing its localization is essential. However, neurexin molecules are notoriously difficult to label, either using immunohistochemistry or via tagged proteins. In the future, it will be important to generate endogenously tagged Nrxn3 animal models to visualize whether Nrxn3 is present at ribbon synapses in hair cells. In recent years, adding endogenous tags to proteins in mice and zebrafish has become more streamlined, making this approach more straightforward (Carrington et al., 2022; Morrow et al., 2021).

### Functional consequences of synapse loss in *Nrxn3* mutants

Currently, no studies have linked hearing loss in humans to the *NRXN3* locus or any other *NRXN* loci. In zebrafish, we found that *nrxn3a; nrxn3b* homozygotes were viable as adults and did not develop any overt vestibular defects (circling behavior or difficultly remaining upright) or reduced acoustic startle responses (Fig. 8C). This suggests that in zebrafish, fewer synapses (Fig. 2; Figs S7 and S8) and decreased synaptic function (Fig. 7) are not sufficient to cause dramatic deficits in these behaviors. It is possible that more subtle behavioral deficits exist in *nrxn3a; nrxn3b* zebrafish mutants. For example, *nrxn3a; nrxn3b* mutants may not be able to sustain responses sufficiently to rheotax (use their lateral line to orient against a constant flow).

Similar to our zebrafish work, mouse *Nrxn3* mutants have normal ABR thresholds despite fewer IHC synapses (Fig. 8A,B). This contrasts with work on mouse *Nlgn1/3* mutants, where a comparable loss of IHC synapses results in a subtle but significant increase in ABR thresholds (12 dB higher at all frequencies) (Ramirez et al., 2022). It is possible that these contrasting behavioral phenotypes could be attributed to differences in genetic manipulations. In our study, we used *Atoh1-Cre* to lesion *Nrxn3* postmitotically in hair cells, whereas *Ngln1/3* mutants were germline KOs. Interesting, *Nlgn1/3* mutant mice also displayed enhanced synapse loss and elevated ABR thresholds after noise exposure. Thus, in mice or zebrafish, loss of Nrxn3 may confer susceptibility to noise or excitotoxic insults that damage ribbon synapses. In the future, it will be interesting to explore the role of *Nrxn3* in more complex hair cell-mediated behaviors and noise exposure paradigms.

Our work shows that Nrxn3 is crucial for synapse maturation in mouse and zebrafish hair cells. Most studies, including ours, use static images to study the maturation of ribbon synapses. However, development is dynamic and best studied in living tissue over time. In future work, we will continue to leverage the zebrafish system – which is ideal for live imaging – to determine the exact role that Nrxn3 plays in synapse maturation *in vivo*. Understanding how hair-cell synapses mature is essential to develop strategies to restore synapses lost due to auditory or vestibular synaptopathy.

## MATERIALS AND METHODS

### Zebrafish strains and husbandry

Zebrafish (*Danio rerio*) were grown at 30°C using a 14 h light/10 h dark cycle. Larvae were raised in E3 embryo medium (5 mM NaCl, 0.17 mM KCl, 0.33 mM CaCl_2_ and 0.33 mM MgSO_4_, pH 7.2). Zebrafish work performed at the National Institutes of Health (NIH) was approved by the Animal Use Committee at the NIH under animal study protocol #1362-13. Larvae were examined at either 3 dpf or 5 dpf unless stated otherwise. The following previously established lines were used in this study: *myo6b:memGCaMP6s^idcTg1^* and *en.sill,hsp70l:GCaMP6s^idcTg8^* (Jiang et al., 2017; Zhang et al., 2018). In addition to these lines, two sanger ENU mutants were obtained from the Zebrafish International Resource Center (ZIRC) and used in this study: *nrxn3a^sa11330^* and *nrxn3b^sa36960^*. The *nrxn3a^sa11330^* mutant results in a premature stop codon in the second LNS domain (C to stop at amino acid 455/1697 in the α isoform, ENSDART00000088179.5). This allele was genotyped using standard PCR and sequencing with the following primer sets: FWD: 5′-AATGAACTCTTTAAAAGGAGCA-3′ and REV: 5′-TCCACTTTTGTGTTCTTCTGGC-3′. The *nrxn3b^sa36960^* mutant results in a point mutation leading to a premature stop codon in the first LNS domain (R to stop at amino acid 135/1687 in the α isoform, ENSDART00000127050.3). This allele was genotyped using standard PCR and sequencing with the following primer sets: FWD: 5′-TCACTGGCACTTTGCTACAATC-3′ and REV: 5′-GTTGGAACCTTATTGCCGTAAC-3′. These mutations are in obligatory exons and are predicted to lead to a premature stop codon and disrupt all α-*nrxn3a* or α-*nrxn3b* splice variants (Fig. S2). Each mutant line was outcrossed three times before use. After outcrossing, the *nrxn3a^sa11330^* and *nrxn3b^sa36960^* mutants were crossed to produce double mutants: *nrxn3a^−/−^; nrxn3b^−/^*^−^. For comparisons, *nrxn3a^−/−^*, *nrxn3b^−/−^* and *nrxn3a^−/−^; nrxn3b^−/^*^−^ mutants were either compared to their respective wild-type siblings or to wild-type larvae collected and grown at the same time as double mutants. For behavioral experiments, *nrxn3a^+/−^; nrxn3b^+/−^* double heterozygotes were compared with double mutants obtained from the same clutch of embryos to directly compare siblings.

### Zebrafish *nrxn3* F0 crispants

We created α-*nrxn3a;* α-*nrxn3b* and β-*nrxn3a;* β-*nrxn3b* F0 crispants to verify the synaptic phenotypes in our germline ENU mutants and to test whether β-*nrxn3b* and *β-nrxn3b* impacts synapse maturation. To create F0 crispants, we injected the following guide sets: α-*nrxn3a* gRNA 5′-GACCACGACAGGCTACACGC(AGG)-3′ and α-*nrxn3b* gRNA 5′-GCACAACTTGCGAACCGTGT(TGG)-3′, or β-*nrxn3a* gRNA 5′-AACACCCGGTCCACAACCCT(CGG)-3′ and β-*nrxn3b* gRNA 5′-AGAGGACGACTGTGCTATCA(AGG)-3′ along with Cas9 protein, as previously described (Hoshijima et al., 2019). These gRNAs are specific to the α or β isoforms of *nrxn3a* and *nrxn3b* and target obligatory exons of each isoform (Fig. S2). After injection, we grew *nrxn3a; nrxn3b* F0 crispants until 5 dpf and performed immunohistochemistry (see below) to assess their synaptic phenotypes. After immunohistochemistry, we genotyped all F0 crispants to ensure that the gRNAs cut their targets robustly via fragment analysis of fluorescent PCR products using the following primers: α-*nrxn3a*_FWD_fPCR 5′-TGTAAAACGACGGCCAGT-GACAAGAACGGCCTACTCAAAGTCT-3′, α-*nrxn3a*_REV_fPCR 5′-GTGTCTT-CAACCCATAAAGTTGTTGCTGA-3′, α-*nrxn3b*_FWD_fPCR 5′-TGTAAAACGACGGCCAGT-GCGTGGACTGTGCAGAAACC-3′, α-*nrxn3ba*_REV_fPCR 5′-GTGTCTTGCCATGCTGCAACTGCCTCC-3′, β-*nrxn3a*_FWD_fPCR 5′-TGTAAAACGACGGCCAGTAGCATGGGGTTTTCTGCATC-3′, β -*nrxn3a*_REV_fPCR 5′-GTGTCTTCCCCTATCGCAATTAACAGCAAG-3′, β-*nrxn3b*_FWD_fPCR 5′-TGTAAAACGACGGCCAGTATGCGCCCCCACTTTAAGAC-3′, β-*nrxn3ba*_REV_fPCR 5′-GTGTCTTCGTGGCCACCTCGTAAAGAGG-3′ (Carrington et al., 2015). Only F0 crispants with robust genomic cutting at both *nrxn3a* and *nrxn3b* target sites (more than a 10-fold reduction in wild-type peak fragment) were included in analyses.

### Zebrafish sparse labeling of single afferents in the lateral line

To visualize the innervation pattern of single afferent neurons, we injected a *neurod1:tdTomato* plasmid at 10 ng/µl, along with 10 ng/µl *tol2* mRNA, into zebrafish embryos at the one-cell stage as previously described (Ji et al., 2018). This plasmid consists of a 5 kb minimal promoter, *neurod1*, that has been shown to drive tdTomato expression in lateral-line afferents (Ji et al., 2018). We screened larvae for tdTomato expression at 3 dpf. Positively identified larvae were prepared for immunostaining at 5 dpf, imaged and analyzed as outlined below.

### Mouse strains and husbandry

The *Nrxn3^flox^* strain was cryorecovered at The Jackson Laboratory from stock JR#014157 (B6;129-*Nrxn3^tm3Sud/J^*; MGI:5437468) (Aoto et al., 2015). In this strain, the first common exon for the α and β transcripts (exon 18) is flanked by *loxP* sites. The *Atoh1-Cre* driver used to inactivate *Nrxn3* in postmitotic hair cells is stock JR#011104 (*B6.Cg-Tg(Atoh1-cre)1Bfri/J*; MGI:3775845; The Jackson Laboratory) (Matei et al., 2005). *Atoh1-Cre; Nrxn3^flox/flox^* mutants were compared with control littermates of the following genotypes: *Atoh1-Cre; Nrxn3^flox/+^*(this control genotype is depicted in the figures), *Nrxn3^flox/flox^, Nrxn3^flox/+^*. The following primers were used for genotyping and to demonstrate *Nrxn3* deletion in cochlear tissue (Fig. S12): *Nrxn3*_F-5′-CACACTTACTTCTGTGGATTGC-3′ and *Nrxn3*_R-5′-CGTGGGGTATTTACGGATGAG-3′. Both males and females were included in the study. Animals were maintained under standard housing conditions (14 h light/10 h dark cycle, ambient temperature and normal humidity). All mouse work was reviewed for compliance and approved by the Animal Care and Use Committee of The Jackson Laboratory.

### Zebrafish immunohistochemistry and imaging

Immunohistochemistry was performed on whole larvae at either 3 dpf or 5 dpf. Whole larvae were fixed with paraformaldehyde (PFA 4%; Thermo Fisher Scientific; 28906) in PBS at 4°C for 3.5 h. For Ca_V_1.3 labeling (Ca_V_1.3, Otoferlin, MAGUK or Ca_V_1.3, Paravalbumin, CTBP), all wash, block and antibody solutions were prepared with PBS+0.1% Tween (PBST). For pre- and postsynaptic labeling (MYO7A, CTBP, MAGUK, Calretinin, SOX2, GFP), all wash, block and antibody solutions were prepared with PBS+1% DMSO, 0.5% Triton X-100, 0.1% Tween-20 (PBDTT). After fixation, larvae were washed four times for 5 min in PBST or PBDDT. For Ca_V_1.3 labeling, larvae were permeabilized with acetone before blocking. For this permeabilization, larvae were washed for 5 min with H_2_O in glass vials. The H_2_O was removed and replaced with ice-cold acetone and larvae placed at −20°C for 5 min, followed by a 5 min H_2_O wash. The larvae were then washed four times for 5 min in PBST. For all immunolabels, larvae were blocked overnight at 4°C in blocking solution (2% goat serum, 1% bovine serum albumin, 2% fish skin gelatin in PBST or PBDTT). After block, larvae were incubated in primary antibodies in antibody solution (1% bovine serum albumin in PBST or PBDTT) overnight, nutating at 4°C. The next day, the larvae were washed four times for 5 min in PBST or PBDTT to remove the primary antibodies. Secondary antibodies in antibody solution were added, and larvae were incubated for 2 h at room temperature, with minimal exposure to light. Secondary antibodies were removed by washing with PBST or PBDTT four times for 5 min. Larvae were mounted on glass slides with Prolong Gold (Thermo Fisher Scientific) using No. 1.5 coverslips. Full details of the primary and secondary antibodies used are in Table S6.

Fixed samples were imaged on an upright LSM 780 or 980 laser-scanning confocal microscope with an Airyscan 2 attachment using Zen (Carl Zeiss) and a 63×/1.4 NA Plan Apo oil immersion objective lens. Airyscan *z*-stacks were acquired every 0.15 µm with a 0.043 µm *xy* pixel size for lateral-line and medial-crista hair cells, and every 0.15 µm with a 0.067 µm *xy* pixel size for hair cells in the anterior macula. The Airyscan *z*-stacks were autoprocessed in 2D. Experiments were imaged with the same acquisition settings to maintain consistency between comparisons. For presentation in figures, images were further processed using Fiji.

### Mouse immunohistochemistry and imaging

Temporal bones were isolated, and an insulin syringe was used to gently flush cold PFA (4%; Electron Microscopy Sciences; 15710) through the cleared oval and round windows after poking a small hole at the cochlear apex. Temporal bones were then immersion-fixed in PFA for 1 h at 4°C, washed in PBS, and rotated overnight in EDTA 4% for decalcification. The next day, cochleae were dissected in three approximate thirds (base, mid and apex) before blocking and permeabilization for 1 h at room temperature under agitation (1% bovine serum albumin; 0.5% Triton X-100). The following primary antibodies were used: CTBP2, GluR2, and mouse anti-MYO7A or Oncomodulin. Primary and secondary (A-21131, A-21240, A-21428 or A-21202, A-31572, A-21447) antibodies were incubated overnight at 4°C in PBS. Samples were washed three times in PBS+0.05% Triton X-100 after each antibody incubation and finally postfixed in PFA 4% for at least 1 h at room temperature. Samples were then mounted flat in Mowiol mounting medium (Calbiochem/MilliporeSigma, 4759041) using two layers of office tape as a spacer for the coverglass (18×18 mm #1.5). Mowiol (10% w/v) was prepared in (25% w/v) glycerol and 0.1 M Tris-Cl (pH 8.5). Full details of the primary and secondary antibodies used are in Table S6.

Mounted samples were imaged on an upright LSM 980 laser-scanning confocal microscope using Zen Blue 3.4 (Carl Zeiss) and a 63×1.4 NA oil objective lens. *Z*-stacks containing six to nine IHCs were acquired every 0.250 µm with an 0.085 µm *xy* pixel size in confocal mode. For presentation in figures, images were further processed using Fiji.

### RNA FISH to detect *nrxn3a* and *nrxn3b* mRNA in lateral-line hair cells

To detect mRNA for *nrxn3a* and *nrxn3b* in zebrafish, we followed the Molecular Instrument RNA FISH Zebrafish protocol, Revision Number 10 (https://files.molecularinstruments.com/MI-Protocol-RNAFISH-Zebrafish-Rev10.pdf), with a few minor changes to the preparation of fixed whole-mount larvae. For our dehydration steps, we dehydrated in a methanol series (25%, 50%, 75%, 100%, 100% methanol), with 5 min for each step in the series. To permeabilize, we treated larvae with 10 µg/ml proteinase K for 20 min. RNA FISH probes were designed to target the long α form of zebrafish *nrxn3a* and *nrxn3b* (Molecular Instrument Probe lot #PRP848 and #PRP849). For *nrxn3a* 12/13 probes were in obligatory exons of α-*nrxn3a.* For *nrxn3b* 19/20 probes were in obligatory exons of α-*nrxn3b* (Fig. S2; Table S5). Although these probe sets are unable to distinguish between splice variants, they should robustly recognize all splice variants of α-*nrxn3a* and α-*nrxn3b* (Fig. S2)*.* In addition to *nrxn3a* and *nrxn3b* probes, we also used *otofb* (Fig. S4I) or bacterial *dapB* (Fig. S4B,D) probes as positive or negative controls, respectively [Molecular Instrument Probe lot #PRP850 (*otofb*, 488 nm), #RTHE541 (*dapB*, 546 nm) and #RTH406 (*dapB*, 647 nm)]. After completing the RNA FISH protocol, we mounted the larvae in ProLong Gold Antifade (Thermo Fisher Scientific, P36930) under 1.5 coverglass. Samples were imaged on an upright LSM 980 laser-scanning confocal microscope with an Airyscan 2 attachment using Zen Blue 3.4 (Carl Zeiss) and a 63×/1.4 NA Plan Apo oil immersion objective lens. Airyscan *z*-stacks were acquired every 0.160 µm with a 0.043 µm *xy* pixel size. The Airyscan *z*-stacks were autoprocessed in 2D. For presentation in figures, images were further processed using Fiji. Our positive control probe (*otofb*) showed robust co-label with α-*nrxn3a* and α-*nrxn3b* mRNAs in hair cells, whereas negative control probes (*dapB*) showed no label in hair cells (Fig. S4A-I). For negative controls, the same settings were used to image *nrxn3* and *dapB* control probes in each channel. RNA FISH labels of *nrxn3a* and *nrxn3b* mRNA were further quantified in Fiji. Quantification of *nrxn3a* and *nrxn3b* mRNA in *nrxn3a; nrxn3b* double mutants (see quantification information below) revealed a 30% and 18% reduction in the number of *nrxn3a* and *nrxn3b* mRNA puncta, respectively (Fig. S9D,E). Similar quantification methods revealed >2 puncta per neuromast in our *dapB* controls. A reduction in *nrxn3a* and *nrxn3b* mRNA puncta suggests that these transcripts undergo some nonsense-mediated decay in *nrxn3a; nrxn3b* germline mutants (El-Brolosy et al., 2019).

### Image processing and quantification of cell counts, synapses, and RNA FISH

*Z*-stack image acquisitions from zebrafish and mouse confocal images were processed in Fiji (Schindelin et al., 2012). Researchers were unaware of genotype during analyses. In zebrafish neuromasts, a Phalloidin label was used to manually score hair-bundle orientation (neuromasts L1-L4) relative to the midline of the muscle somites. Hair-cell numbers were counted manually based on MYO7A, Oncomodulin, Parvalbumin or Otoferlin labeling. Supporting-cell counts were scored manually based on SOX2 label. Posterior lateral-line ganglion neurons were counted manually based on *en.sill,hsp70l:GCaMP6s* expression (after a GFP immunolabel).

To identify and quantify puncta (presynaptic, postsynaptic, Ca_V_1.3 cluster or RNA FISH puncta), an automated synapse quantification method using a customized Fiji-based macro, ‘Complete Synapse Counter v5.2’ (https://github.com/KindtLab/CompleteSynpaseCounter5.2) was used. Before automated puncta quantification, each channel was background subtracted using rolling-ball radius background subtraction. Then each *z*-stack was max-intensity projected. A mask was generated by manually outlining the region of interest (ROI) (e.g. hair cells) in the reference channel. This mask was then applied to the *z*-projection of each synaptic component or RNA FISH channel. Each masked image was then analyzed using the automated Fiji macro. In this macro, the images were thresholded using an adaptive thresholding plugin by Qingzong TSENG (https://sites.google.com/site/qingzongtseng/adaptivethreshold) to generate a binary image of the puncta. Individual synaptic or RNA FISH puncta were then segmented using the particle analysis function in Fiji. For particle analysis, the following minimum size thresholds were applied: zebrafish lateral-line images – CTBP 0.025 μm^2^, MAGUK 0.04 μm^2^, Ca_V_1.3 0.025 μm^2^, *nrxn3a*, *nrxn3b* and *dapB* RNA FISH particles 0.03 μm^2^ and 0.01 μm^2^; zebrafish inner ear images – CTBP 0.025 μm^2^, MAGUK 0.025 μm^2^; mouse IHCs – CTBP 0.025 μm^2^, GluR2 0.025 μm^2^. A circularity factor between 0.1 and 0.5 was also applied to particle analysis. A watershed was applied to the particle analysis result to break apart overlapping synaptic components. After the watershed, the particle analysis was rerun with size and circularity thresholds to generate ROIs and measurements of each synaptic or RNA FISH component. The ROIs were applied to the original *z*-projection to get the average intensity and area of each punctum.

To recognize paired synaptic components, images were further processed using ‘Complete Synapse Counter v5.2’. Here, the overlap and proximity of ROIs from different channels (e.g. pre- and postsynaptic puncta) were calculated. ROIs with positive overlap or ROIs within 2 pixels were counted as paired or partner components. The ROIs were applied to the original *z*-projection to get the average intensity and area of each paired or unpaired punctum.

Some image datasets required a preprocessing step before entry into the ‘Complete Synapse Counter v5.2’. This includes zebrafish samples imaged at 3 dpf and our mouse IHC datasets. For the preprocessing step, the volumes were segmented in VVDviewer (https://github.com/JaneliaSciComp/VVDViewer). Staining outside of the hair cell was manually segmented or removed using VVDviewer. After this segmentation, the *z*-stacks were max-intensity projected and processed using the ‘Complete Synapse Counter v5.2’ macro.

### Quantification of lateral-line afferent terminal areas and single afferent selectivity

To quantify the area occupied by the four to six afferent terminals beneath lateral-line neuromasts, we examined *z*-stacks of Calretinin-labeled terminals. Each *z*-stack was opened in FIJI and max-intensity projected. Projected images were autothresholded to isolate the terminals, and the mean area within the threshold was measured.

Larvae with positive tdTomato expressions in individual afferent neurons were stained with CTBP and MAGUK to label pre- and postsynapses, as well as Phalloidin to visualize hair-bundle orientations. After the immunostaining, single afferent terminals were imaged using a Zeiss LSM 980 confocal microscope in Airyscan mode as described above. *Z*-stacks were then loaded in VVDviewer for 3D viewing of afferent terminals and their connections with hair cells. Hair-cell numbers and their orientations were assigned manually based on the weak membrane labeling of MAGUK and Phalloidin label, respectively. To assign synapses and hair cells to terminals, we manually identified all the complete synapses (paired CTPB and MAGUK puncta) that colocalized with each tdTomato terminal. This allowed us to determine the number of hair cells innervated and the number of complete synapses formed per terminal.

To analyze the selectivity of individual terminals, we used a selectivity index. This index was defined as percentage of the number of hair cells with the domination orientation innervated, divided by the total number of hair cells innervated. Here, an index value of 50% indicates that the afferent terminal showed no selectivity when choosing between the two different orientations, whereas 100% indicates perfect selectivity towards a single orientation.

### Calcium imaging of lateral-line hair cells and afferents

For functional imaging, 4-6 dpf larvae were anesthetized in 0.04% Tricaine-S (tricaine methanesulfonate, Western Chemical, TRS1), pinned to a Sylgard-filled perfusion chamber at the head and tail, and paralyzed by injection of 125 µM α-bungarotoxin (Tocris, 2133) into the heart cavity, as previously described (Lukasz and Kindt, 2018). Larvae were then rinsed three times in E3 embryo media to remove the tricaine. Next, larvae were rinsed three times with extracellular imaging solution (in mM: 140 NaCl, 2 KCl, 2 CaCl_2_, 1 MgCl_2_ and 10 HEPES, pH 7.3, OSM 310±10) and allowed to recover. Stimulation was achieved by a fluid jet, which consisted of a pressure clamp (HSPC-1, ALA Scientific) and glass pipette, pulled and broken to an inner diameter 40-50 µm, and filled with extracellular imaging solution. A 500 ms pulse of positive or negative pressure was used to deflect the hair bundles of mechanosensitive hair cells along the anterior-posterior axis of the fish. Hair cells of the two orientations (P to A and A to P) were stimulated separately. Stimuli that deflected kinocilia 5-15 µm were included in the analysis, as these deflections represent saturating stimuli that do not induce damage.

Hair-cell responses to stimuli were imaged using an A1R laser-scanning confocal scan head on an upright Nikon NI-E microscope with a resonant scanner and a 60×/1.0 NA CFI Fluor water immersion objective equipped with a z-piezo. Acquisition was controlled with Nikon Elements Advanced Research v.5.20.02. GCaMP6s fluorescence was excited with a 488 nm solid-state laser passed through a standard 405/488/561/640 BS20/80 dichroic and collected with a 560 nm low-pass dichroic and 525/50 emission filter. Images were acquired using a GaAsP PMT and 4× averaging. Pixel size for presynaptic imaging was 0.28 µm; pixel size for MET imaging was 0.14 µm. Each neuromast (L2 or L3) was stimulated four times (starting with a P-to-A stimulus and alternating between the two directions) with an inter-stimulus interval of ∼2 min. This enabled us to collect presynaptic responses (collected first) and hair-bundle responses to both stimulus directions for each neuromast. Three *z*-slices (1.5 µm step size for presynaptic responses; 0.5 µm step size for hair bundle responses) were collected per timepoint for 110 timepoints at a frame rate of 33 ms for a total of ∼100 ms per *z*-stack and a total acquisition time of ∼11 s. Stimulation began at timepoint 31; timing of the stimulus was triggered by an outgoing voltage signal from Nikon Elements.

Calcium responses in the afferent process were acquired on a Swept-field confocal system built on a Nikon FN1 upright microscope (Bruker) with a 60×/1.0 NA CFI Fluor water-immersion objective. The microscope was equipped with a Rolera EM-C2 EMCCD camera (QImaging), controlled using Prairie view 5.4 (Bruker). GCaMP6 s was excited using a 488 nm solid state laser. We used a dual band-pass 488/561 nm filter set (59904-ET, Chroma). Pixel size for postsynaptic imaging was 0.27 µm. Stimuli were delivered as outlined above for hair-cell responses. Each neuromast (L2, L3 or L4) was stimulated twice with an inter-stimulus interval of ∼2 min. Five *z*-slices (1.0 µm step) were collected per timepoint for 80 timepoints at a frame rate of 20 ms for a total of ∼100 ms per *z*-stack and a total acquisition time of ∼8 s. Stimulation began at timepoint 31; timing of the stimulus was triggered by an outgoing voltage signal from Prairie view.

Acquired images were converted into TIFF series for processing. Researchers were unaware of genotype during analysis. *Z*-stacks were average projected, registered and spatially smoothed using a Gaussian filter (size=3, sigma=2) in custom-written MatLab software as described previously (Zhang et al., 2018; https://github.com/KindtLab/CompleteSynpaseCounter5.2). The first ten timepoints (∼1 s) were removed to reduce the effect of initial photobleaching. Registered average projections were then opened in Fiji for intensity measurements. Using the Time Series Analyzer V3 plugin, circular ROIs (18×18 pixels for presynaptic responses; 8×8 pixels for hair-bundle responses, 12×12 pixels for afferent process) were placed on hair bundles or synaptic sites; average intensity measurements over time were measured for each ROI, as described previously (Lukasz and Kindt, 2018). Neuromasts were excluded in the case of motion artifacts. Hair-bundle responses were excluded if they responded to stimuli of both directions. All other data were included in analyses. Presynaptic responses were defined as >10% ΔF/F0 within the 500 ms stimulus or >20% within 1 s of stimulus onset. Hair-bundle responses were defined as >15% ΔF/F0 within the 500 ms stimulus and >15% in the 500 ms after the stimulus. Postsynaptic responses were defined as >5% ΔF/F0 and a minimum duration of 500 ms. Square wave responses indicate movement artifacts and were excluded. Calcium imaging data were further processed in Prism 10 (Graphpad). The first 20 timepoints were averaged to generate an F0 value, and all responses were calculated as ΔF/F0. Responses presented in figures represent average responses of synaptically active cells within a neuromast. The max ΔF/F0 was compared between wild-type animals and double mutants. To measure baseline GCaMP6s intensities in hair bundles, the presynaptic region, or afferent terminals, mean GCaMP6s intensity was measured during the prestimulus (2 s) time window. For the images acquired in this time window, the GCaMP6 images were autothresholded, and the mean intensity was measured at each time point. Then all the time points during the prestimulus time window were averaged for each neuromast.

### Mouse ABR tests

All tests were performed in a sound-attenuating chamber. Animals between P28 and P32 were anesthetized with a mix of ketamine and xylazine (1 mg and 0.8 mg per 10 g of body weight, respectively). Body temperature was maintained at 37°C using a heating pad (FHC Inc.). Animals were then tested using the RZ6 Multi-I/O Processor System coupled to the RA4PA 4-channel Medusa Amplifier (Tucker-Davis Technologies). Subdermal needles were used as electrodes, with the active electrode inserted at the cranial vertex, the reference electrode under the left ear and the ground electrode at the right thigh. ABRs were recorded after binaural stimulation in an open field by tone bursts at 8, 16, 32 and 40 kHz generated at 21 stimuli/second, and a waveform for each frequency/dB level was produced by averaging the responses from 512 stimuli. ABR thresholds were obtained for each frequency by reducing the sound pressure level (SPL) by 5 dB between 90 and 20 dB. We compared waveforms by simultaneously displaying three or more dB levels on screen at the same time to identify the lowest level at which an ABR waveform could be recognized. Wave I amplitudes were measured by annotating the peak and trough of the first ABR waveform and calculating the difference (nV), and wave I delay was measured at the peak of the first wave (ms). In these experiments, controls consist of a pool of *0* (Cre-negative)*; Nrxn3^flox/+^*, *0; Nrxn3^flox/flox^* and *Atoh1-Cre; Nrxn3^flox/+^* animals. Each control genotype was also compared separately (Fig. S17C-E).

### Zebrafish startle behavior

A Zantiks MWP behavioral system was used to examine acoustic startle responses. Behavioral trials were performed at 5 dpf, on three independent days. For this behavioral analysis, we compared *nrxn3a^+/−^; nrxn3b^+/−^* double heterozygotes with *nrxn3a^−/−^; nrxn3b^−/−^* double mutants for an in-clutch, sibling comparison. *Nrxn3a^+/−^; nrxn3b^+/−^* double heterozygotes showed a slight (12%) yet significant reduction in complete synapses compared with wild-type controls. We also compared *nrxn3a^+/−^; nrxn3b^+/−^* double heterozygotes and *nrxn3a^−/−^; nrxn3b^−/−^* double mutants with wild-type animals born the same day; this analysis revealed no difference in startle response between these genotypes.

The Zantiks system tracked and monitored behavioral responses via a built-in infrared camera at 30 frames per second. A 12-well plate was used to house larvae during behavioral analysis. Each well was filled with E3 and one larva. All fish were acclimated in the plate within the Zantiks chamber in the dark for 15 min before each test. To induce startle, an integrated stepper motor was used to drive a vibration-induced startle response. A vibrational stimulus that triggered a maximal percentage of animals startling in controls without any tracking artifacts (due to the vibration) was used for our strongest stimuli. Each larva was presented with each vibrational stimulus five times with 100 s between trials. For each animal, the proportion of startle responses out of the five trials was plotted. During the tracking and stimulation, a Cisco router connected to the Zantiks system was used to relay the *xy* coordinates of each larva every frame. To qualify as a startle response, a distance above 4 pixels or ∼1.9 mm was required within two frames after stimulus onset. Animals were excluded from our analysis if no tracking data was recorded for the animal.

### Experimental design and statistical analysis

Statistical analyses and data plots were performed using Prism 9 or 10 (Graphpad). All the data were plotted individually. Values of data with error bars on graphs and in text are expressed as mean±s.e.m. unless stated otherwise. A power analysis was performed to estimate the approximate sample sizes needed. All zebrafish experiments were performed on a minimum of four animals, seven neuromasts. Primary posterior lateral-line neuromasts with A-P orientations L1-L4 were used for all experiments except Ca_V_1.3 immunostains, which examined L1, L2 and DV1 neuromasts, or sideview neuromast images in Fig. S6 where O1 neuromasts were imaged. For 5 dpf larvae, each neuromast represents analysis of 12-20 hair cells and 41-68 synapses. For mouse studies, all experiments were performed on at least four mutants and four siblings at P28 or P42 for the synapse analyses and P28-P32 for the ABR analysis. For mouse synapse quantification, at least two ROIs containing 6-9 IHCs were examined from each region of the cochlea (centrally located within the apex, mid or base region) for each animal. Each datapoint in Fig. 6 and Fig. S13 represents the average number of synapses per hair cell for each ROI. For mouse ABR analysis, eight *Nrxn3* mutants and at least five animals from each control genotype were compared. All replicates are biological. Samples were prepared, scored and imaged unaware of genotype whenever possible. Where appropriate, data were confirmed for normality using a D'Agostino-Pearson normality test. For pairwise comparisons, an unpaired two-tailed *t*-test was used if the data passed normality tests. If the data failed normality tests, a Mann–Whitney *U*-test was used. For multiple comparisons, a one-way or two-way ANOVA with Sidak's multiple comparison post-hoc test was used.

## Supplementary Material



10.1242/develop.202723_sup1 Supplementary information
