# Supplementary material for: Presynaptic Nrxn3 is essential for ribbon-synapse maturation in hair cells
Source: Development. 2024 Oct 10;151(19):dev202723. doi: 10.1242/dev.202723 (PMC11488651; doi:10.1242/dev.202723)
Supplement: Supplementary information [file develop-151-202723-s1.pdf]

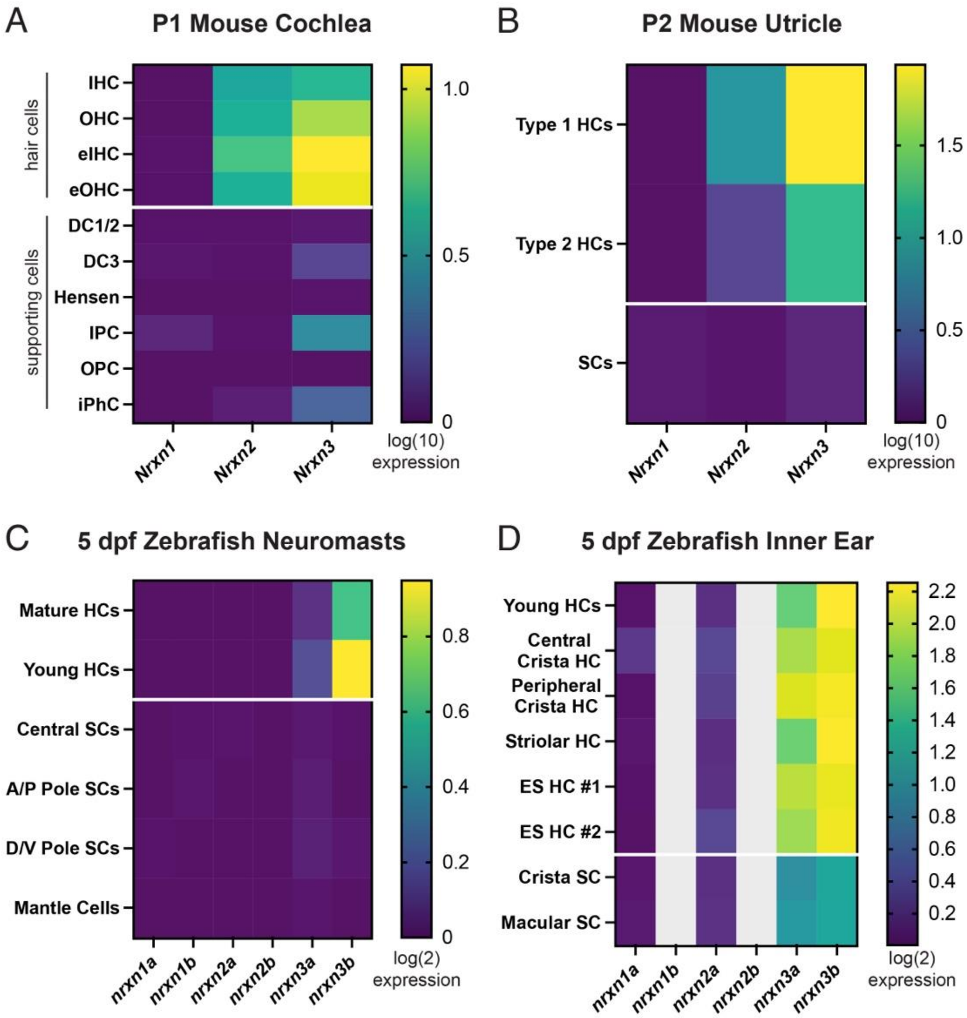

Fig. S1. Expression of neurexins in mouse and zebrafish hair-cell sensory systems

(A-D) Heatmaps showing the cell-type-specific expression of the neurexin family in mouse cochlear epithelium at P1 (A), mouse utricle at P2 (B), zebrafish lateral-line neuromasts at 5 dpf (C), and zebrafish inner ears at 5 dpf (D). Gray in (D) indicates that the gene was not detected in the dataset. HC: hair cell; SC: supporting cell; IHC: inner hair cell; OHC: outer hair cell; eIHC: early inner hair cell; eOHC: early outer hair cell; DC1/2: Deiter cells from rows 1 and 2; DC3: Deiter cells from row 3; Hensen: Hensen cells; IPC: inner pillar cells; OPC: outer pillar cells; iPhC: inner phalangeal cells; A/P: anterior/posterior; D/V: dorsal/ventral; ES: extrastriolar. Data was extracted from existing scRNAseq datasets in gEAR (Fabian et al., 2022; Kolla et al., 2020; Lush et al., 2019; Orvis et al., 2021; Shi et al., 2023). Also see Supplemental Tables 1-4.

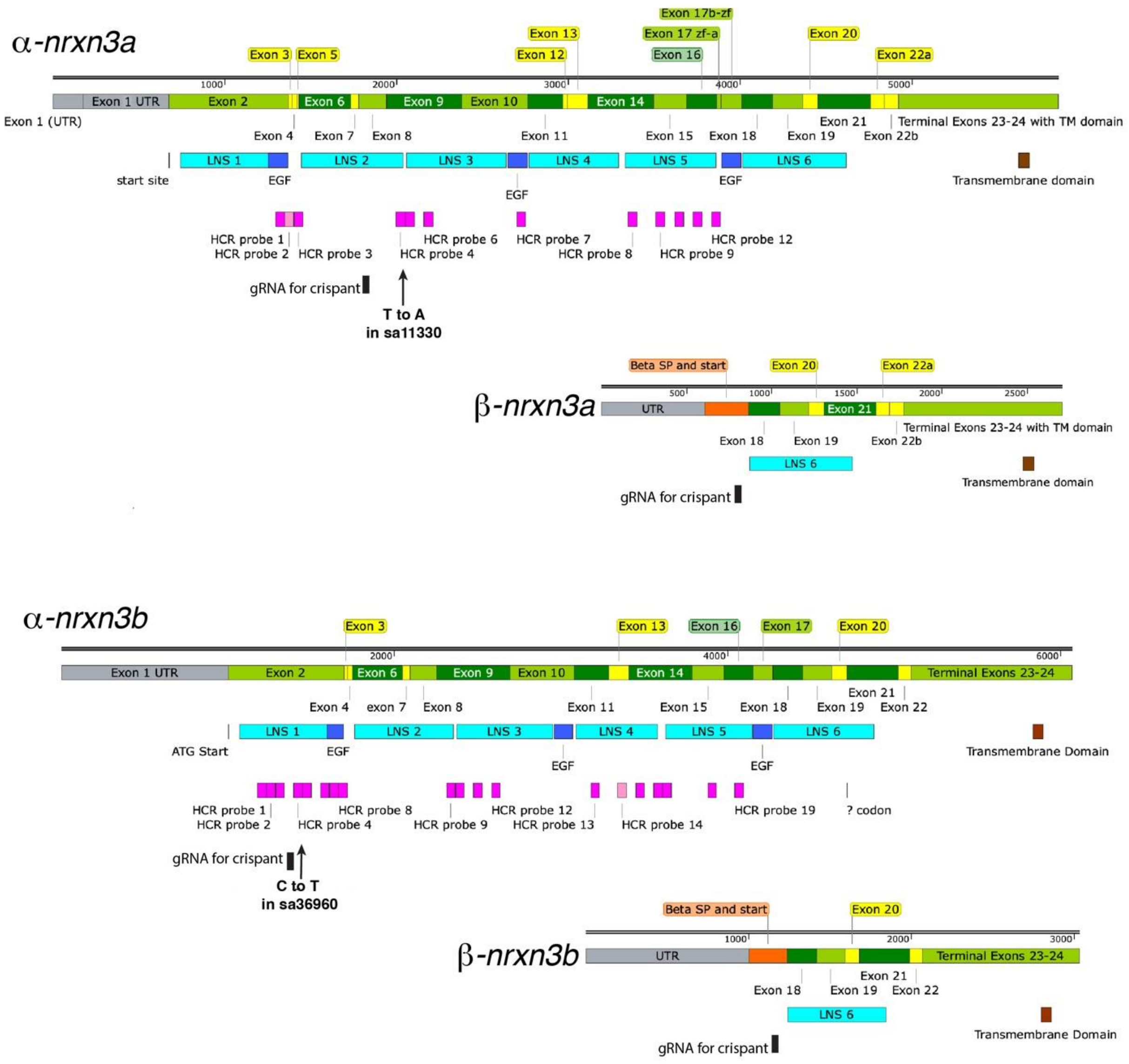

Fig. S2. Predicted zebrafish *nrxn3a* and *nrxn3b* splice variants.

Top and bottom panels show predicted or confirmed exons for zebrafish *nrxn3a* and *nrxn3b.* Green (light and dark) indicates exons that are obligatory (present in all splice variants). Yellow indicates exons that have been shown to be alternatively spliced. RNA FISH probes used to detect α-*nrxn3* mRNAs are indicated in pink. Light pink indicates a probe that detects an alternatively spliced exon. The position of the nonsense inducing germline lesions in α-*nrxn3a* and α-*nrxn3b* (T to A sa11330 and C to T sa36960) are shown with black arrows. Both lesions are present in obligatory exons (exon 9 for α-*nrxn3a* and exon 2 for α-*nrxn3b*). Black boxes

Development • Supplementary information

show the location of the gRNAs used for the F0 crispant analyses. The unique exons for β-*nrxn3a* and β-*nrxn3b* are shown in orange. The *nrxn3a* and *nrxn3b* exons are numbered based on human α-*NRXN3* exons and splice variants. Note that zebrafish α-*nrxn3b* has no predicted exons 5 or 12 and just one variant of exon 22, while zebrafish α-*nrxn3a* has 2 variants of exon 17. For simplicity the numerous splice variants that alter the intracellular C-terminus of both α and β are not shown (exons 22-24). Images were made in Snapgene.

Development • Supplementary information

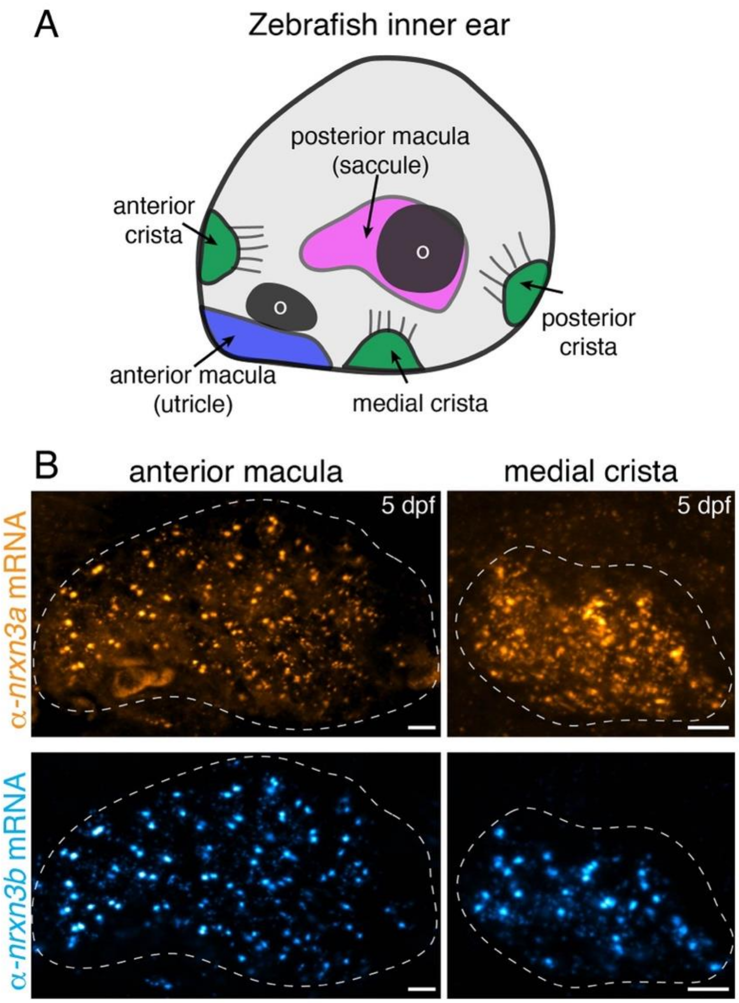

Fig. S3. α-*nrxn3a* and α-*nrxn3b* mRNAs are present in zebrafish inner-ear hair cells.

(**A**) Schematic showing a larval zebrafish inner ear. Within the inner ear, clusters of hair cells are present in 3 cristae and 2 maculae. Each macula is associated with an otolith (o). (**B**) RNA FISH analysis reveals that both α-*nrxn3a* (orange) and α-*nrxn3b* (cyan) mRNAs are present in inner-ear hair cells. The dashed line in B outlines the locations of hair cells within the sensory epithelium obtained using an *otofb* mRNA co-label (not shown). Images are from larvae at 5 dpf. Scale bars = 5 µm in B.

Development • Supplementary information

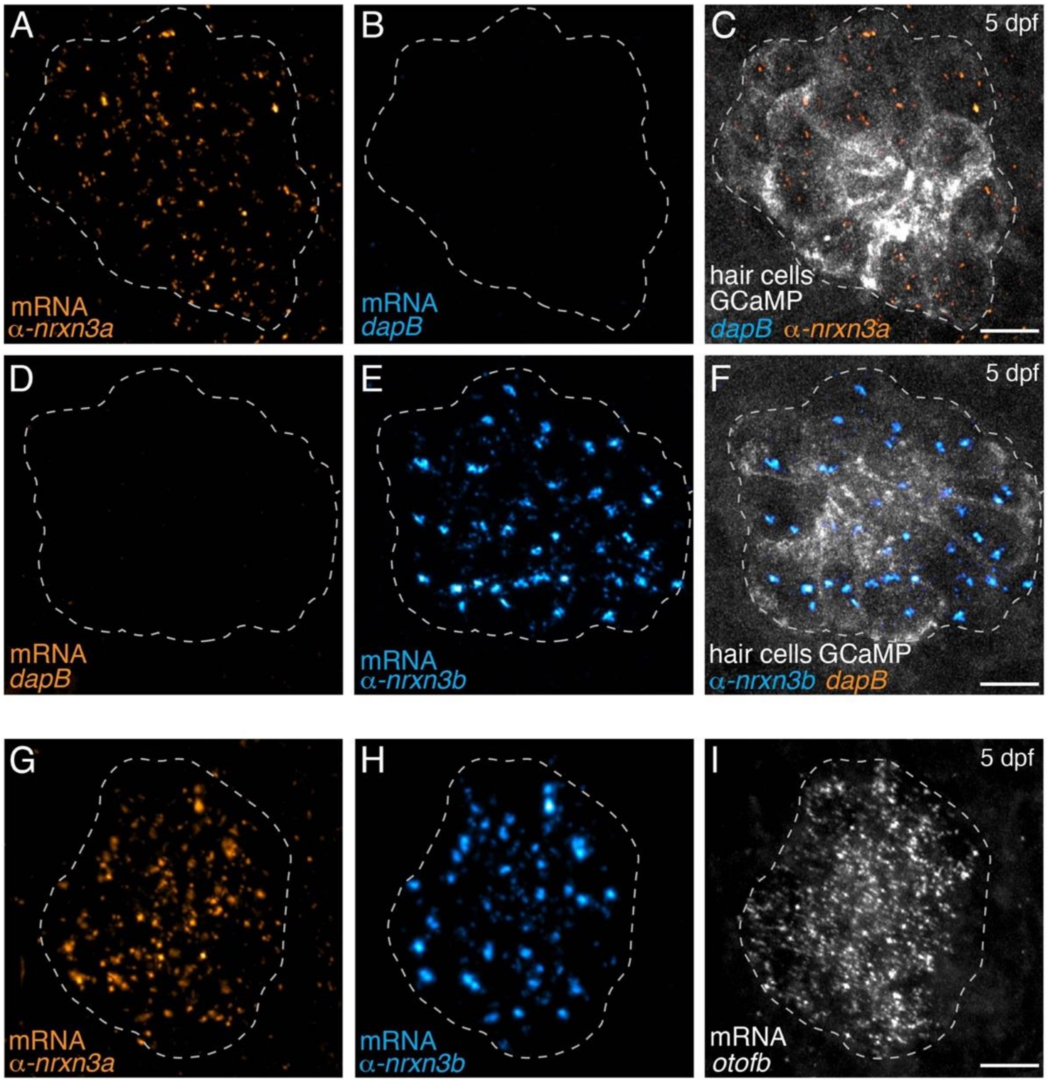

Fig S4. α-*nrxn3a*, α-*nrxn3b* and *otofb* mRNAs, but not control *dapB* mRNAs are present in lateral-line hair cells.

(**A-F**) RNA FISH reveals that both α-*nrxn3a* (A, orange) and α-*nrxn3b* (E, cyan) probes label lateral-line hair cells. The negative control probe *dapB* (B, cyan; D, orange) does not label hair cells. In C and F, hair cells (*myo6b:memGCaMP6s*) are labeled in grayscale. The dashed lines in A-F outline the locations of hair cells obtained using the *myo6b:memGCaMP6s* label. All images for negative controls were acquired and displayed using the same settings for each channel. (**G-I**) RNA FISH reveals that probes for α-*nrxn3a* (G, orange), α-*nrxn3b* (H, cyan) and the positive control probe *otofb* (I, gray) all label lateral-line hair cells. The dashed lines in G-I outline the locations of hair cells obtained using the *otofb* mRNA label. Scale bar = 5 µm in C, F and I.

Development • Supplementary information

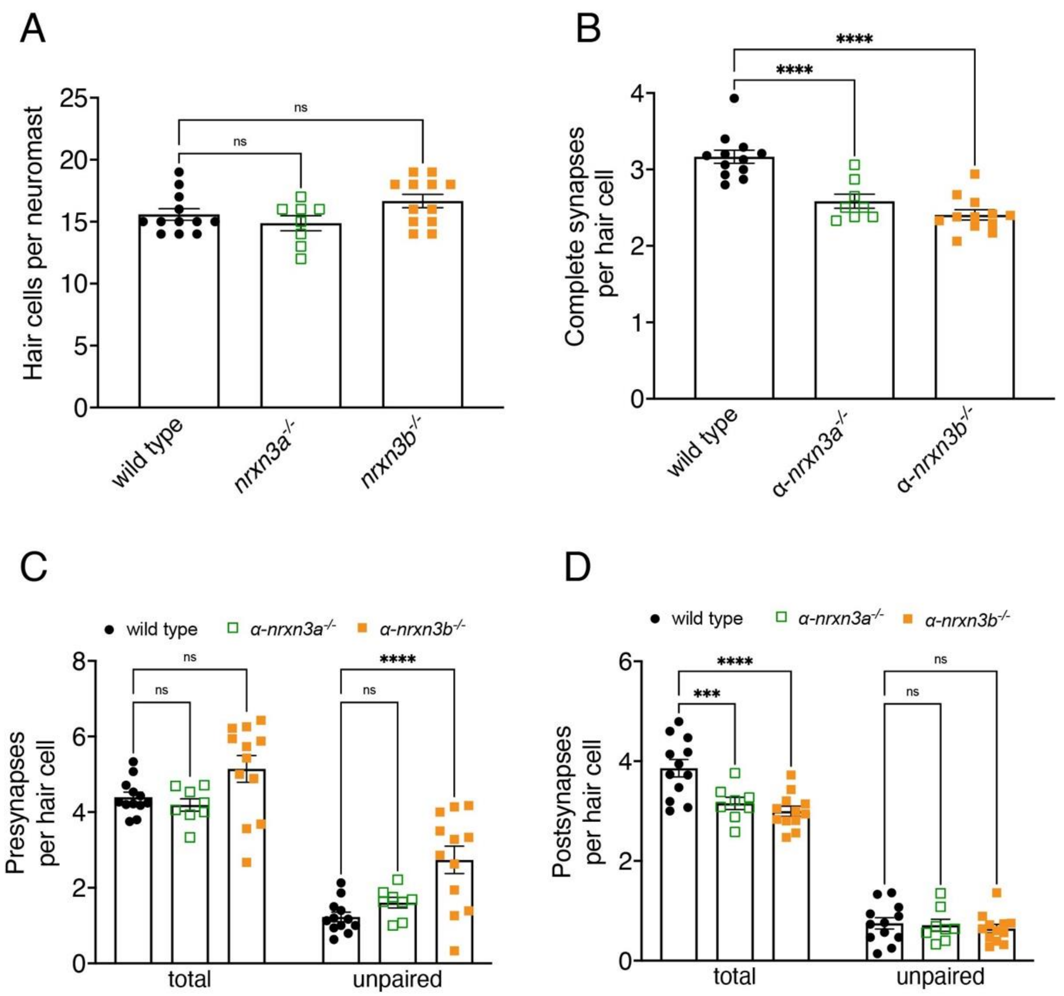

Fig. S5. Minor defects in synapse organization in lateral-line hair cells are observed in α-*nrxn3a* and α-*nrxn3b* single mutants at 5 dpf.

(**A-F**) Quantification reveals that while both α-*nrxn3a* and α-*nrxn3b* single mutants have a similar number of hair cells per neuromast (A), each mutant has significantly fewer complete synapses per hair cell compared to wild-type controls (B). The total number of pre-synapses are the same across all genotypes but there are significantly more unpaired presynapses in *nrxn3b* mutants (C). The total number of postsynapses per hair cell is significantly reduced in both in α-*nrxn3b* and α-*nrxn3a* single mutants compared to wild-type controls. In contrast, the number of unpaired postsynapses per hair cell is the same across all genotypes (D). N = 12 wild-type, 8 α-*nrxn3a* and 12 α-*nrxn3b* mutant neuromasts in A-D at 5 dpf. A one-way ANOVA was used in A-B, while a 2-way ANOVA was used in C-D. ns P > 0.05, *P < 0.05, ***P < 0.001, ****P < 0.0001.

Development • Supplementary information

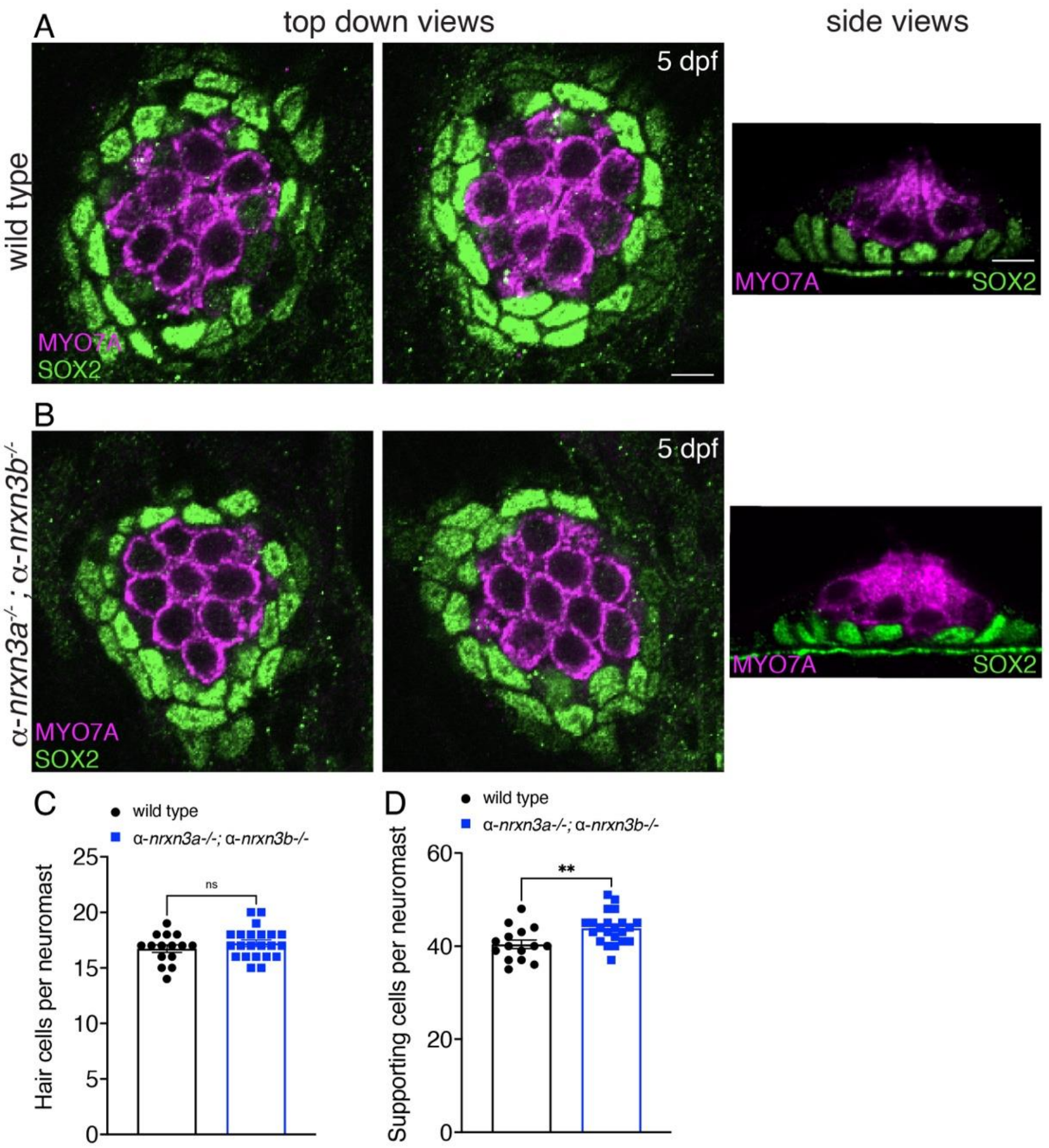

Fig. S6. Neuromast morphology and supporting cell counts are largely normal in α-*nrxn3* mutants.

(**A-B**) Immunohistochemistry was used to examine supporting cells (SOX2) and hair cells (MYO7A) in neuromast organs at 5 dpf in wild-type controls (A) and α-*nrxn3a;* α-*nrxn3b* mutants (B). Both top down and side views of neuromasts are shown for each genotype. Overall, the morphology and positioning of hair cells and supporting cells is normal in both genotypes. (**C-D**) Quantification reveals that while the number of hair cells per neuromast are unchanged (C, wild type: 16.7; α-*nrxn3a;* α-*nrxn3b:* 17.2), the number of supporting cells is slightly higher in α-*nrxn3a;* α-*nrxn3b* mutants (D, wild type: 40.4; α-*nrxn3a;* α-*nrxn3b:* 43.8). N = 22 wild-type and n = 15 α-*nrxn3a;* α-*nrxn3b* mutant neuromasts. An unpaired t-test was used for comparisons. ns P > 0.05, **P < 0.01. Scale bar = 5 µm in A.

Development • Supplementary information

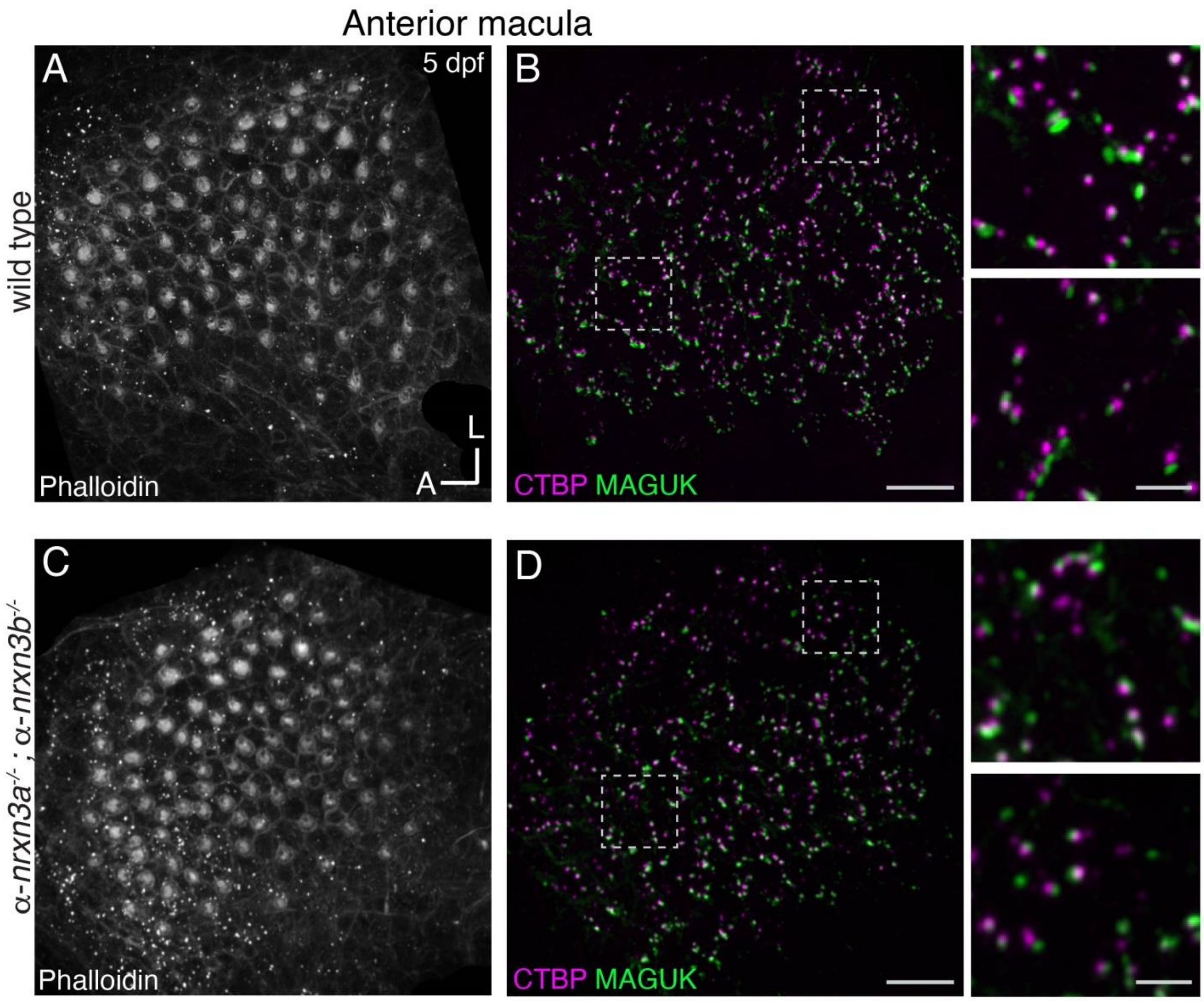

Fig. S7. Loss of α-Nrxn3 results in fewer synapses hair cells in the zebrafish anterior macula. (**A-D**) Confocal images of the anterior macula (utricle) in wild-type controls (A-B, top panels) and α-*nrxn3a;* α-*nrxn3b* mutants (C-D, bottom panels). Phalloidin labels the apical hair bundles (A,C) while pan-CTBP labels the presynapses (magenta), and pan-MAGUK labels postsynapses (green) in B and D. The dashed lines indicate regions used to create the insets on the right side of panels B and D. The higher magnification insets show that there are fewer complete synapses in α-*nrxn3a;* α-*nrxn3b* mutants compared to wild-type controls. Images were taken from larvae at 5 dpf. Scale bars = 10 µm in A-D, 5 µm in the insets.

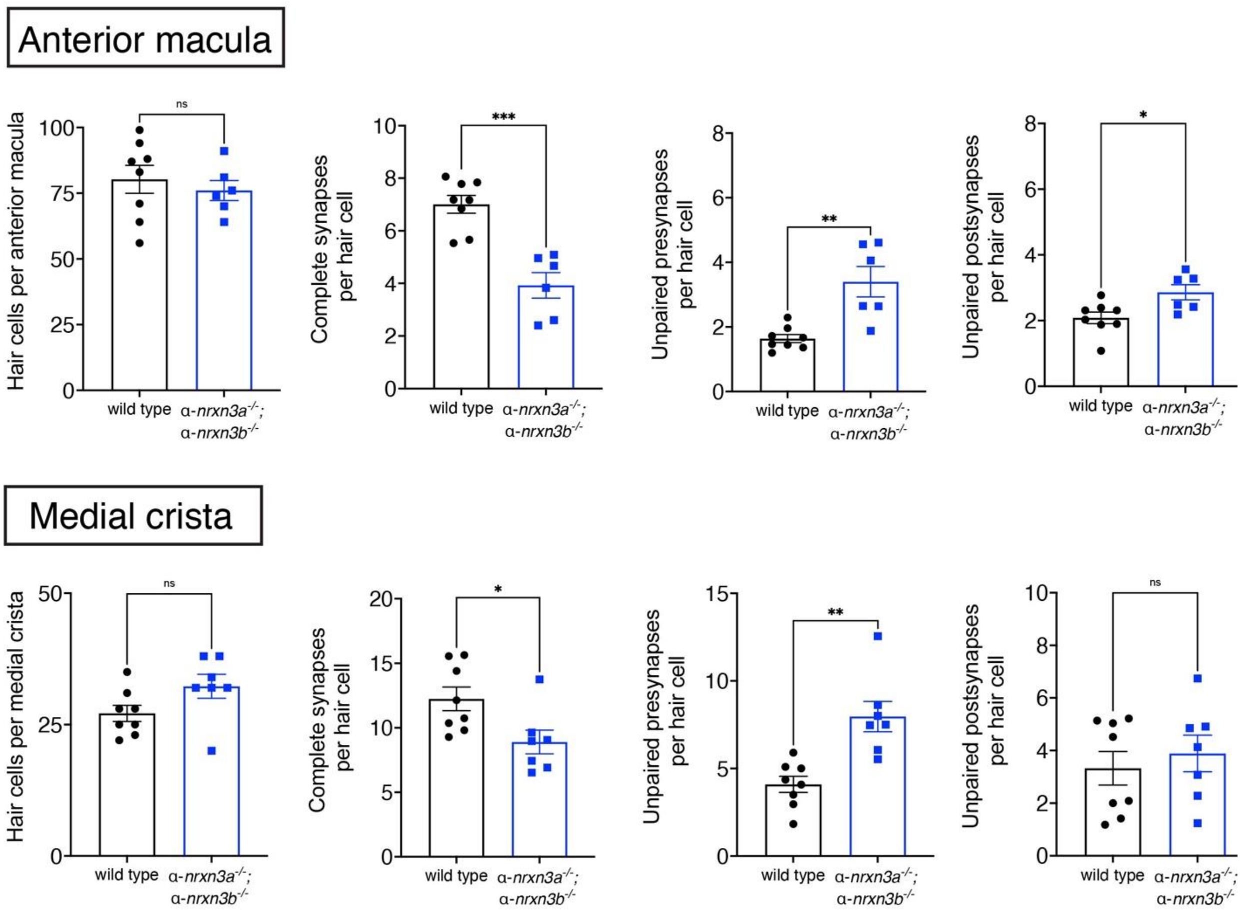

Fig. S8. Quantification of synapse loss in anterior macula and medial crista in α-*nrxn3a;* α-*nrxn3b* mutants.

Quantification reveals that wild-type controls and α-*nrxn3a;* α-*nrxn3b* mutants have a similar number of hair cells per anterior macula and medial crista. There are significantly fewer complete synapses per hair cell in each epithelium in α-*nrxn3a;* α-*nrxn3b* mutants compared to wild-type controls. Along with fewer complete synapses, there are significantly more unpaired presynapses per hair cell in α-*nrxn3a;* α-*nrxn3b* mutants compared to wild-type controls in both the anterior macula and medial crista. There are also more unpaired postsynapses per hair cell in the anterior macula, but not the media crista in α-*nrxn3a;* α-*nrxn3b* mutants compared to wild-type controls. N = 8 wild-type and n = 6 α-*nrxn3a;* α-*nrxn3b* mutant anterior maculae, n = 8 wild-type and n = 7 α-*nrxn3a;* α-*nrxn3b* mutant medial cristae. Quantifications are from larvae at 5 dpf. An unpaired t-test was used for comparisons. ns $P > 0.05$, $^{*}P < 0.05$, $^{**}P < 0.01$, $^{***}P < 0.001$.

Development • Supplementary information

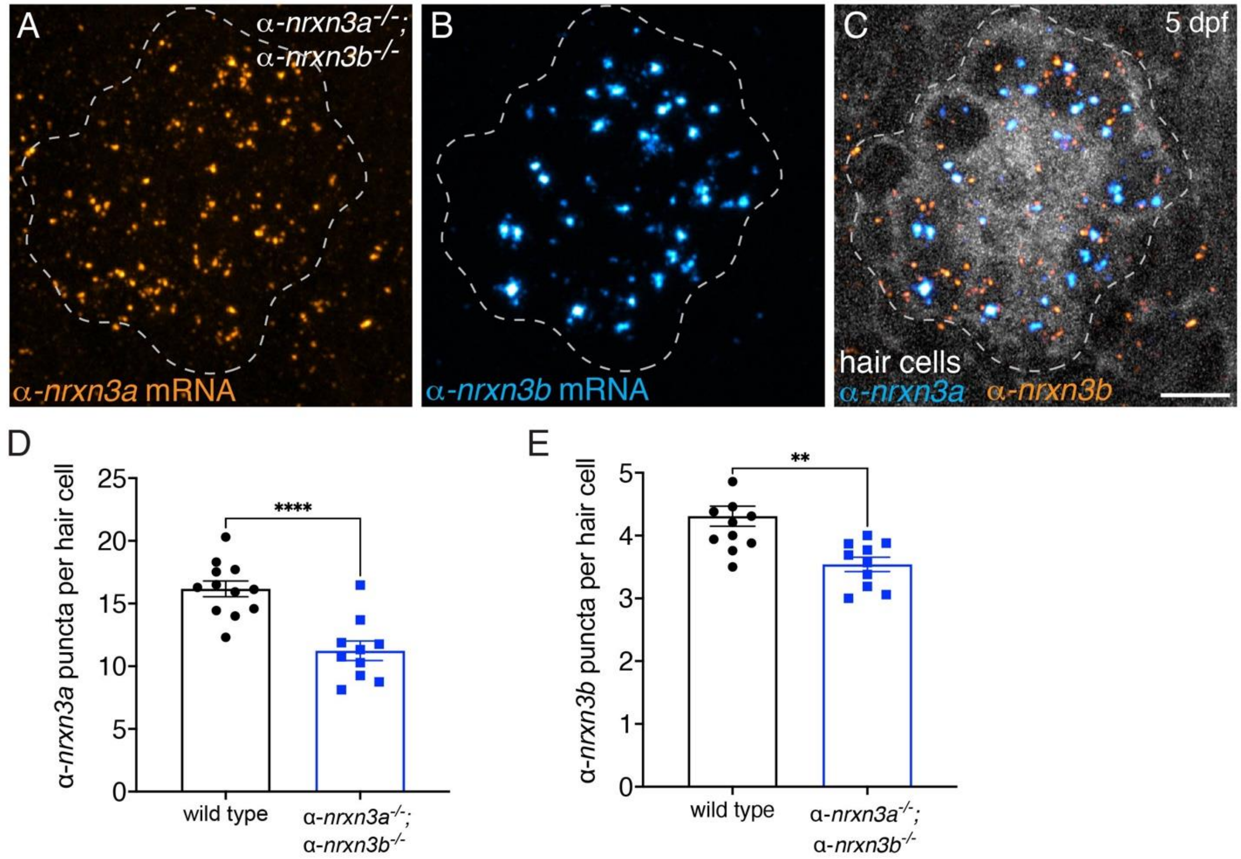

Fig. S9. α-*nrxn3a* and α-*nrxn3b* mRNAs are reduced in lateral-line hair cells in zebrafish α-*nrxn3a;* α-*nrxn3b* mutants.

(**A-C**) RNA FISH reveals that both α-*nrxn3a* (A, orange) and α-*nrxn3b* (B, cyan) mRNAs are present in lateral-line hair cells of α-*nrxn3a;* α-*nrxn3b* ENU germline mutants. In C, hair cells (*myo6b:memGCaMP6s*) are labeled in grayscale. The dashed lines in A-C outline the locations of hair cells obtained using the *myo6b:memGCaMP6s* label. (**D-E**) Quantification reveals that the number of α-*nrxn3a* (D) and α-*nrxn3b* (E) puncta are reduced in α-*nrxn3a;* α-*nrxn3b* mutants compared to wild-type controls. An unpaired t-test was used in D-E, n = 12 wild-type and 10 α-*nrxn3a;* α-*nrxn3b* mutant neuromasts at 5 dpf. $^{**}P < 0.01$, $^{****}P < 0.0001$. Scale bar = 5 µm in C.

Development • Supplementary information

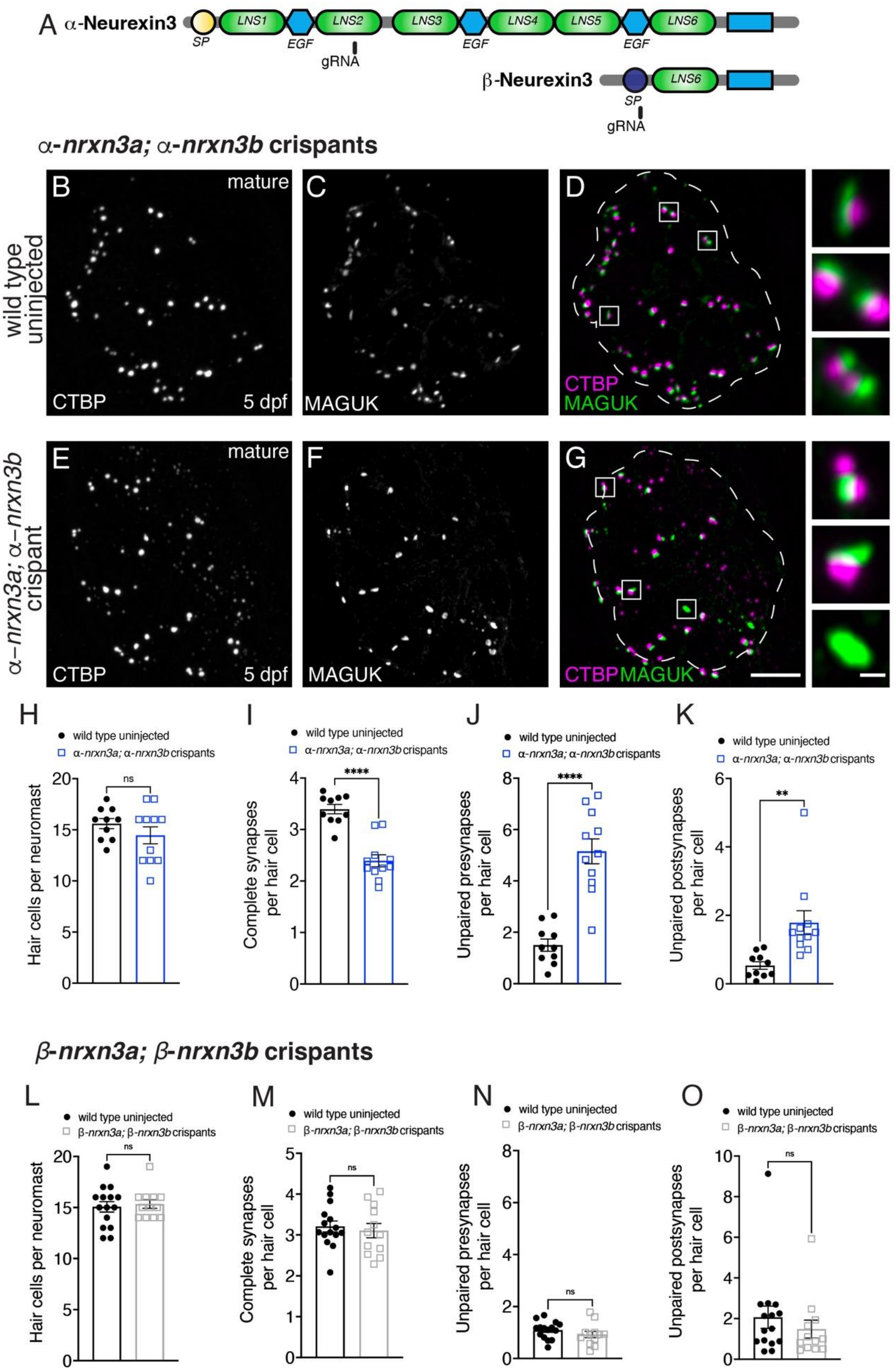

Development • Supplementary information

Fig. S10. F0 crispants demonstrate that α but not β Nrxn3 is required for synapse maturation in zebrafish.

(**A**) Schematic of α and β Nrxn3 isoforms in zebrafish. The location of the gRNAs used to disrupt each isoform of *nrxn3a* and *nrxn3b* is indicated. (**B-G**) Confocal images of mature neuromasts (5 dpf) from wild-type controls (B-D) and α-*nrxn3a;* α-*nrxn3b* F0 crispants (E-G). Pan-CTBP labels the presynapses (B,E) and pan-MAGUK labels the postsynapses (C,F). Merged images are shown in D and G. Dashed lines in D and G outline the hair-cell region in each image (obtained from a MYO7A co-label (not shown)). The insets to the side in D and G show 3 examples of individual synapses. (**H-K**) Quantification reveals that wild-type controls and *α-nrxn3a;* α-*nrxn3b* crispants have a similar number of hair cells per neuromast (H). There are significantly fewer complete synapses per hair cell in *α-nrxn3a;* α-*nrxn3b* crispants compared to wild-type controls (I). Along with fewer complete synapses, there are significantly more unpaired presynapses (J) and postsynapses (K) per hair cell in *α-nrxn3a;* α-*nrxn3b* crispants. N = 10 wild-type and 11 α-*nrxn3a;* α-*nrxn3b* crispant neuromasts at 5 dpf. (**L-O**) Quantification reveals that wild-type controls and β-*nrxn3a;* β-*nrxn3b* crispants have a similar number of hair cells per neuromast (L). The number of complete synapses (M), unpaired presynapses (N), and postsynapses (O) per hair cell is similar in wild-type controls and β-*nrxn3a;* β-*nrxn3b* crispants. N = 15 wild-type and 12 β-*nrxn3a;* β-*nrxn3b* crispant neuromasts at 5 dpf. An unpaired t-test was used in H-K and L-O. ns P > 0.05, ** P < 0.001, ****P < 0.0001. Scale bars = 5 µm in G, 0.5 µm in the inset in G.

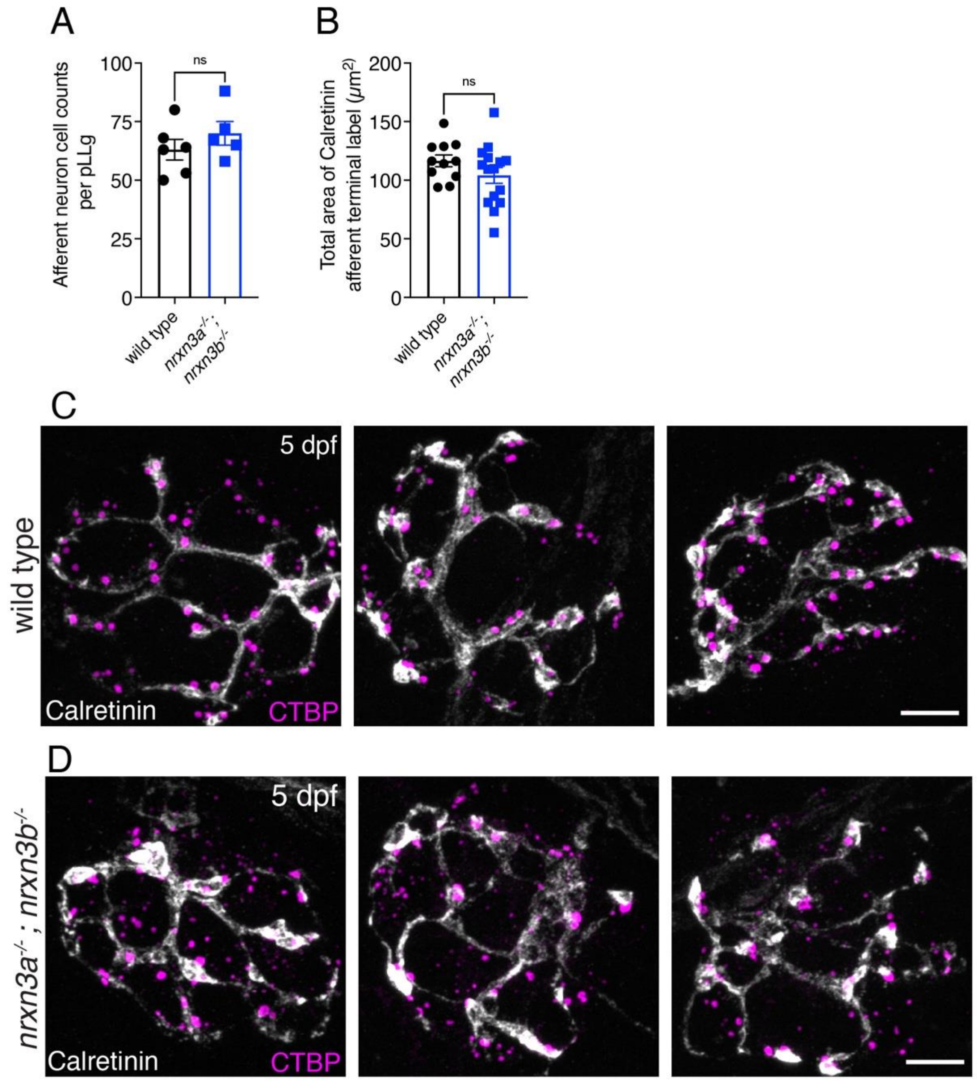

Fig. S11. Loss of Nrxn3 does not impact the number of pLL afferent neurons or the area of afferent terminals beneath lateral-line hair cells.

(**A**) Quantification of *en.sill,hsp70l:GCaMP6s* label reveals that the number pLL afferent neurons is the same in wild-type controls and *nrxn3a; nrxn3b* mutants. N = 6 wild-type and 5 *nrxn3a; nrxn3b* pLL ganglia at 5 dpf. (**B**) Quantification of Calretinin label reveals that the area of the afferent terminals is similar between wild-type controls and *nrxn3a; nrxn3b* mutants. N = 11 wild-type and 15 *nrxn3a; nrxn3b* mutant terminals at 5 dpf. (**C-D**) Immunolabeling of CTBP labels the presynapse while Calretinin labels the afferent terminals beneath lateral-line hair cells in wild type (C) and *nrxn3a; nrxn3b* mutants (D). An unpaired t-test was used in A-B. ns $P > 0.05$.

Development • Supplementary information

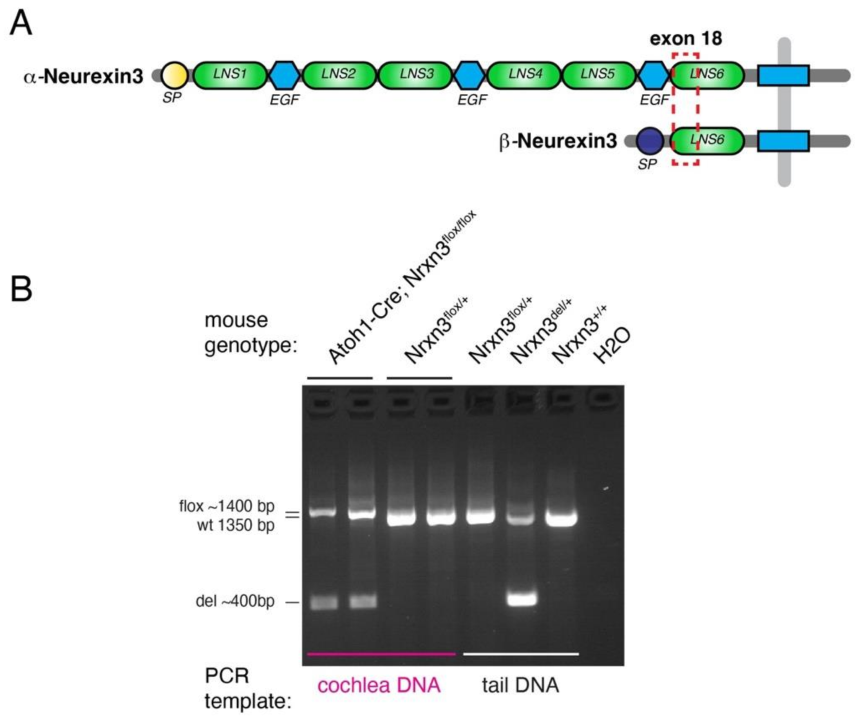

Fig. S12. Deletion of *Nrxn3* exon 18 is detected in the mouse cochlea after *Atoh1-Cre* recombination.

(**A**) Schematic of the mouse α and β *Nrxn3* isoforms. The location of the obligatory exon 18 is indicated. Deletion of exon 18 disrupts both α and β *Nrxn3*. (**B**) Genomic DNA used as template for PCR was either extracted from the P7 cochlear floor including the auditory epithelium (4 first lanes) or from a tail biopsy at weaning age. An animal carrying a constitutive *Nrxn3*^{del} allele was used as positive control. Note the exon 18 delection is detected in conditional *Nrxn3* mutants (*Atoh1-Cre; Nrxn3*$^{flox/\mathrm{flox}}$) but not in control (*Nrxn3*$^{flox/+}$) cochleae.

Development • Supplementary information

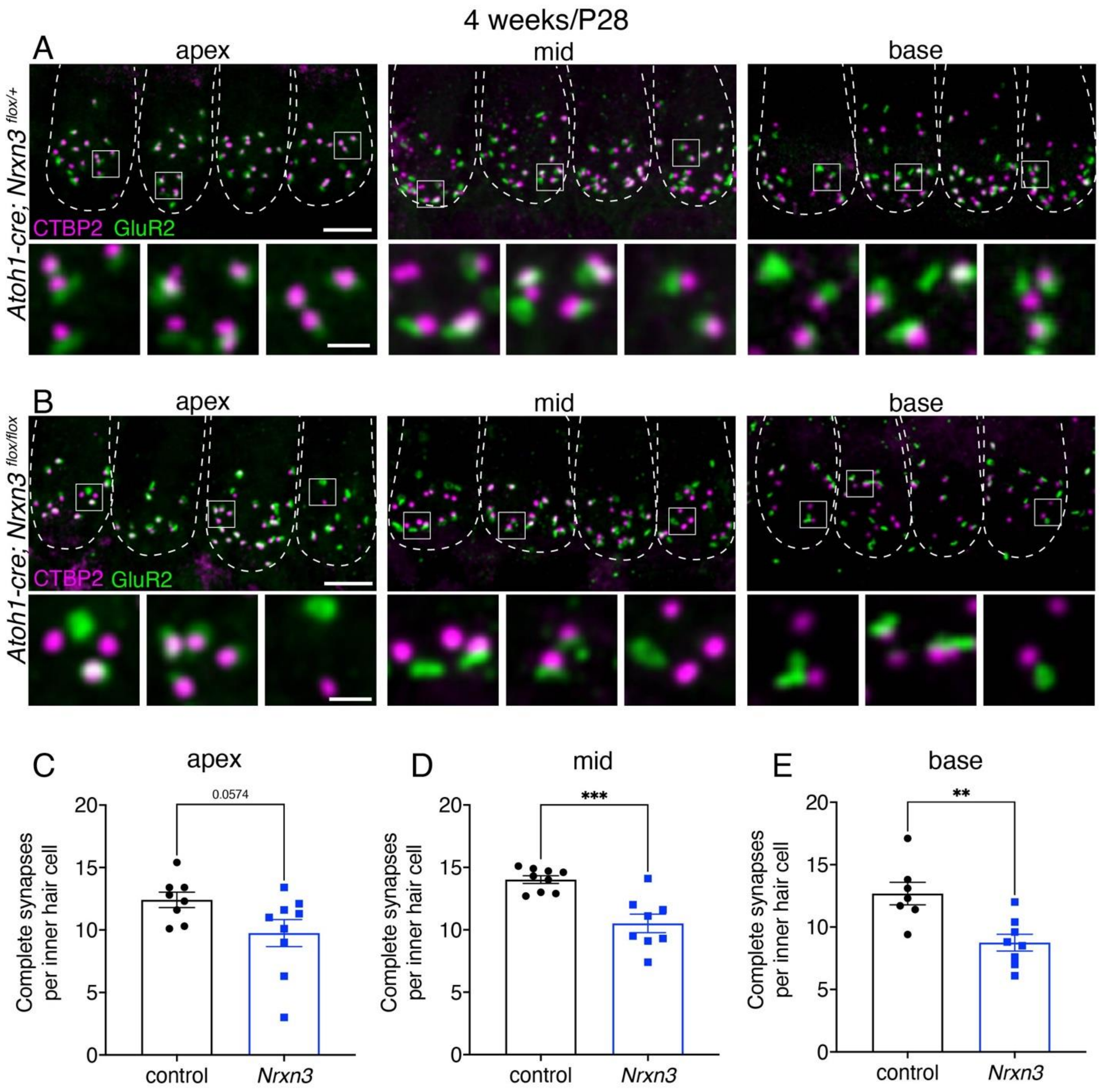

Fig. S13. NRXN3 is required at 4 weeks for proper synapse numbers in mouse auditory inner hair cells.

(**A-B**) Confocal images of 4-week old (P28) mouse auditory IHCs from control (A) and *Nrxn3* mutant animals (*Atoh1-Cre; Nrxn3*$^{flox/flox}$) (B). CTBP2 labels the presynapses, and GluR2 labels the postsynapses. Merged images show 4 IHCs from 3 different regions of the cochlea (apex, middle, basal thirds) for each genotype. Dashed lines indicate the outlines of hair-cell bodies in each image (obtained from a Oncomodulin co-label (not shown)). White boxes in top panels are magnified in insets below to highlight synapses more clearly. (**C-E**) Quantification reveals that

Development • Supplementary information

compared to controls, *Nrxn3* mutants have significantly fewer complete synapses per IHC at the mid (D) and base (E), and a reduced but not significant decrease at the apex (C). These findings were compiled from 4 animals of each genotype and from 2 independent litters and immunostains. Each dot represents the average synapse number from 1 imaging region (6-9 IHCs). Two imaging regions were examined per animal for each tonotopic region. An unpaired t-test was used in C-E. **P < 0.01, ***P < 0.001. Scale bar = 5 μm in A and 1 μm in the insets.

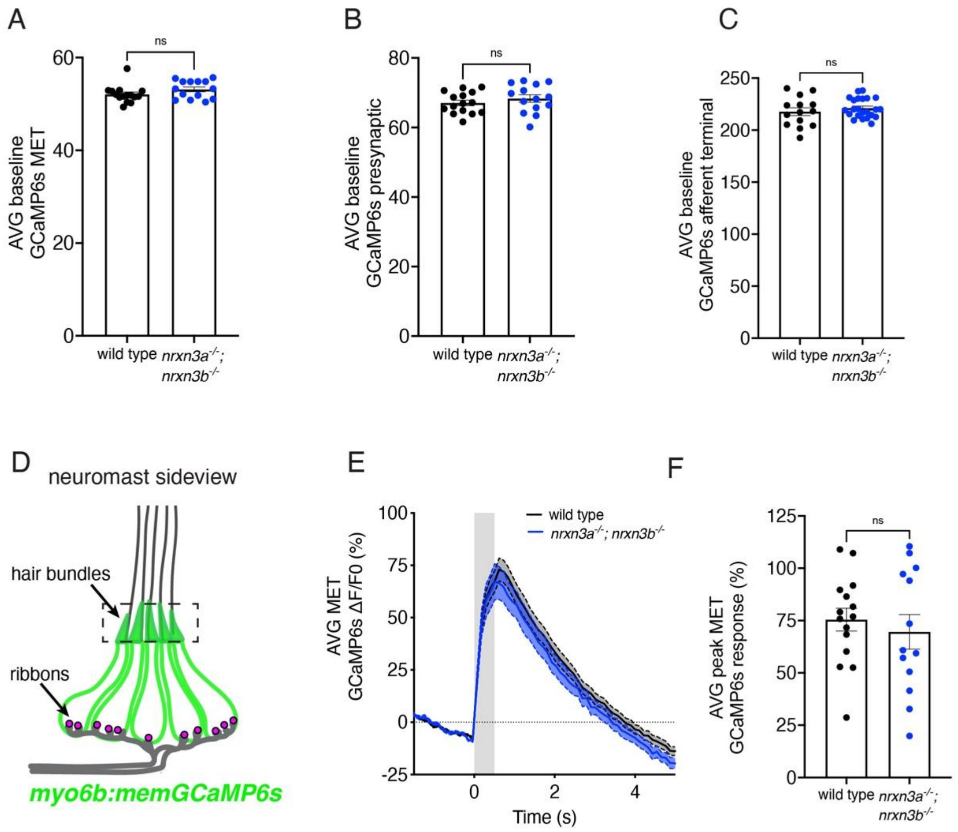

Fig. S14. Loss of Nrxn3 does not impact evoked mechanosensitive responses or baseline GCaMP6s levels in lateral-line hair cells or afferent terminals.

(**A-C**) Quantification of the mean baseline GCaMP6s intensity in hair bundles (A), presynapse (B) and afferent terminals (C) shows no difference between wild-type controls and *nrxn3a; nrxn3b* mutants. In A-B, N = 15 wild-type and 15 *nrxn3a; nrxn3b* mutant neuromasts at 5-6 dpf; in C, N = 14 wild-type and 23 *nrxn3a; nrxn3b* mutant terminals at 4-5 dpf. (**D**) Schematic of a neuromast shown from the side expressing memGCaMP6s in hair cells. The region used to measure GCaMP6s mechanosensitive (MET) responses in hair bundles is indicated with a dashed box. (**E**) ΔF/F0 GCaMP6s traces showing average MET GCaMP6 response during a 500 ms fluid-jet stimulation (grey area) for wild-type controls (black) and *nrxn3a; nrxn3b* mutants (blue). Traces are displayed as mean, dashed lines are SEM. (**F**) Quantification of the maximum ΔF/F0 MET calcium GCaMP6 response reveals no difference between for wild-type controls and *nrxn3a; nrxn3b* mutants. N = 15 wild-type and 13 *nrxn3a; nrxn3b* mutant neuromasts at 5-6 dpf. An unpaired t-test was used in A-C and F. ns P > 0.05.

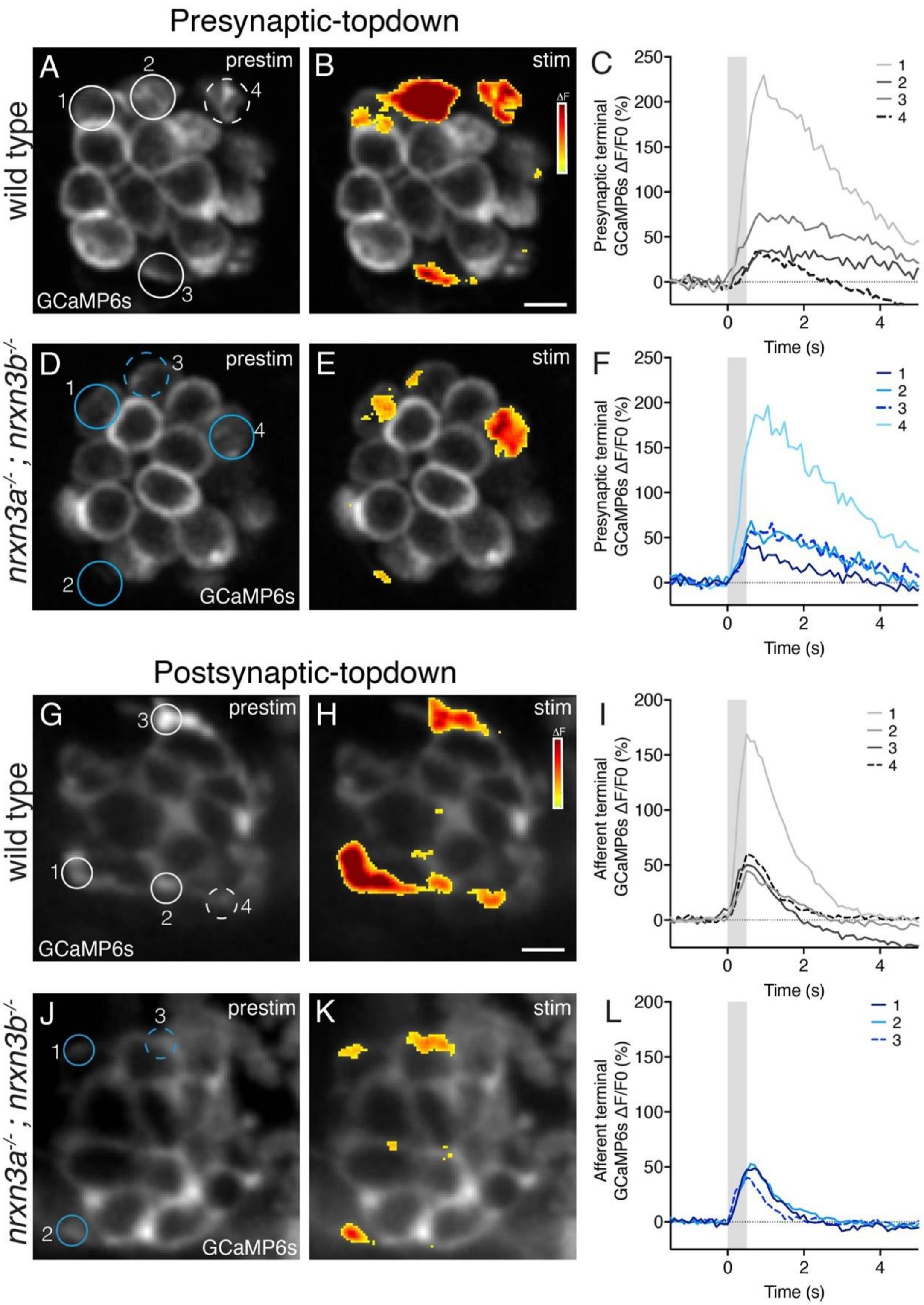

Presynaptic-topdown
A
prestim
1
2
4
3
GCaMP6s
wild type
B
stim
ΔF
C
Presynaptic terminal
GCaMP6s ΔF/F0 (%)
Time (s)
D
prestim
nrxn3a-/- ; nrxn3b-/-
GCaMP6s
E
stim
F
Postsynaptic-topdown
G
prestim
H
stim
I
Afferent terminal
GCaMP6s ΔF/F0 (%)
J
prestim
K
stim
L

Fig. S15. Nrxn3 is required for proper hair-cell synapse function in the lateral line.
(**A-F**) ΔF heatmaps show spatial patterns of presynaptic GCaMP6s increases in hair cells before (A,D) and during (B,E) a 500 ms fluid-jet stimulation in a wild-type (A,B) and a *nrxn3a; nrxn3b* mutant (D,E) neuromast. ROIs indicate synaptically active hair cells and examples of regions used to measure the average response per neuromast (anterior (P to A) responses solid ROI, posterior (A to P) responses dashed ROI). The black area in the center of each cell is the nucleus. Traces in C and F show ΔF/F responses from ROIs in A and D. Gray area indicates timing of stimulus. (**G-L**) ΔF heatmaps show spatial patterns of postsynaptic GCaMP6s increases in hair cells before (G,J) and during (H,K) a 500 ms fluid-jet stimulation in a wild-type (G,H) and *nrxn3a; nrxn3b* mutant (J,K) neuromast. ROIs indicate synaptically active terminals and examples of regions used to measure the average terminal response per neuromast (anterior (P to A) responses solid ROI, posterior (A to P) responses dashed ROI). Traces in I and J show ΔF/F responses from ROIs in G and J. Gray area indicates timing of stimulus. Scale bar in B and H = 5 µm. Wild-type examples in A-B and G-H correspond to the same example in Fig 7 B,C and G,H.

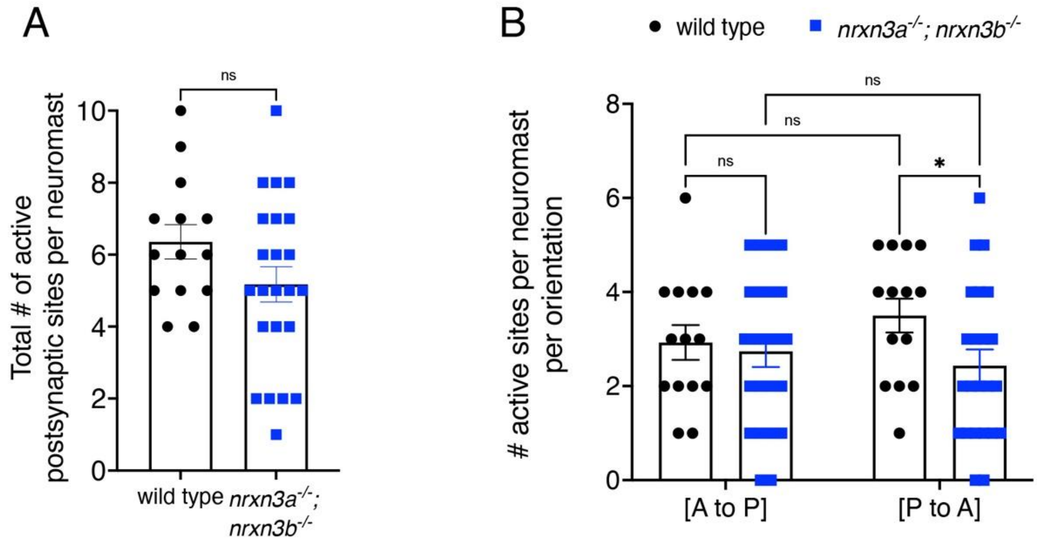

Fig. S16. Loss of Nrxn3 reduces the number of (P to A) responses in lateral-line afferent terminals.

(**A-B**) Quantification of the number of active GCaMP6s postsynaptic sites per neuromast in wild-type controls and *nrxn3a; nrxn3b* mutants. There was no difference in the total number of active sites per neuromast between genotypes (A). But when the active sites were split into those responding to anterior (P to A) or posterior (A to P) flow, there was a significant reduction in the number of (P to A) responses between genotypes (B). N = 14 wild-type and 23 *nrxn3a; nrxn3b* mutant neuromasts at 4-5 dpf. An unpaired t-test was used in A, and a 2-way ANOVA was used in B. ns P > 0.05, * P<0.05.

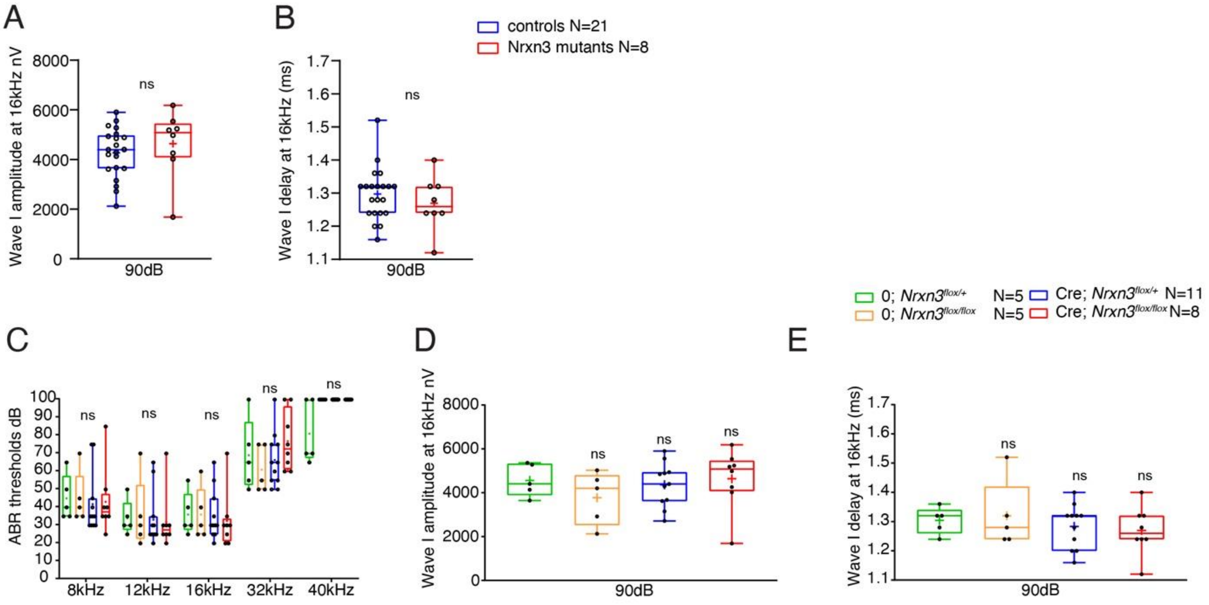

Fig. S17. Wave-I kinetics and ABR thresholds are similar among all mouse control groups and *Nrxn3* mutants.

(**A-B**) No difference was observed between the combined control groups and *Nrxn3* mutants with regards to wave-I amplitude (A) and wave-I delay (B). N = 21 control and 8 *Nrxn3* mutants. (**C-E**) There was no difference in ABR threshold in the 3 control groups (*no Cre; Nrxn3$^{flox/+}$, no Cre; Nrxn3$^{flox/flox}$, Atoh1-Cre; Nrxn3$^{flox/+}$*) and *Nrxn3* mutant animals (*Atoh1-Cre; Nrxn3$^{flox/flox}$*) at P28-P32 (C). No difference was observed between any of the control groups and *Nrxn3* mutants with regards Wave-I amplitude (D) or Wave-I delay (E). N = 5 *no Cre; Nrxn3$^{flox/+}$*, N = 5 *no Cre; Nrxn3$^{flox/+}$*, N = 11 *Atoh1-Cre; Nrxn3$^{flox/+}$* and N = 8 *Atoh1-Cre; Nrxn3$^{flox/flox}$* mutants. Distributions are framed with 25-75% whisker boxes where exterior lines show the minimum and maximum, the middle line represents the median, and + represents the mean. A Mann-Whitney test was used in A,B,D and E. A 2-way ANOVA was used in C.

Development • Supplementary information

Table S1. Expression of *nrxns* in the zebrafish lateral line

All data is presented as log_2, from a scRNAseq dataset from 5 dpf zebrafish, generously provided in gEAR. Citation: (Lush et al., 2019).

	nrxn1a	***nrxn1b***	***nrxn2a***	***nrxn2b***	***nrxn3a***	***nrxn3b***
**Mature HCs**	0	0	0	0	0.0792556	0.6445266
**Young HCs**	0	0	0	0	0.1614415	0.9483396
**Central Cells**	0	0.00424979	0.00789814	0	0.0214946	0.0040762
**A/P Pole SCs**	0	0.01327939	0	0	0.032712	0
**D/V Pole SCs**	0.00705773	0	0	0	0.0395409	0.0180281
**Mantle Cells**	0	0	0	0	0.0113735	0

HC: hair cell; SC: supporting cell; A/P: anterior/posterior; D/V: dorsal/ventral

Table S2. Expression of *nrxns* in the zebrafish inner ear

All data is presented as log_2 from a scRNAseq dataset from 5 dpf zebrafish generously provided in gEAR. Citations: Originally produced by (Fabian et al., 2022) and re-analyzed by (Shi et al., 2023).

	nrxn1a	***nrxn1b***	***nrxn2a***	***nrxn2b***	***nrxn3a***	***nrxn3b***
**Nascent HCs**	0.017244	N/A	0.182318	N/A	1.669498	2.252925
**Central Crista HC**	0.241899	N/A	0.337531	N/A	1.924604	2.137349
**Peripheral Crista HC**	0.010784	N/A	0.281368	N/A	2.106929	2.214861
**Crista SC**	0.035205	N/A	0.181564	N/A	0.951794	1.216517
**Striolar HC**	0.038361	N/A	0.162834	N/A	1.70053	2.24259
**ES HC #1**	0.00997	N/A	0.186522	N/A	1.97859	2.162395
**ES HC #2**	0.004016	N/A	0.333883	N/A	1.879131	2.193123
**Macular SC**	0.059702	N/A	0.202364	N/A	1.047868	1.194818

HC: hair cell; SC: supporting cell; ES: extrastriolar; N/A indicates that the gene was not detected in the dataset

Development • Supplementary information

Table S3. Expression of *Nrxns* in Mouse Cochlea

All data is presented as log_{10}, from a scRNAseq dataset from P1 mouse cochlea, generously provided in gEAR (Orvis et al., 2021). Citation: (Kolla et al., 2020).

	Nrxn1	*Nrxn2*	*Nrxn3*
**IHC**	0	0.5693218	0.6620762
**OHC**	0	0.6251518	0.9122078
**eIHC**	0.006435404	0.7321457	1.072232
**eOHC**	0.003664268	0.6247755	1.035732
**DC1/2**	0.007857317	0.005367244	0.02147482
**DC3**	0.01723814	0.007261817	0.152594
**Hensen**	0	0	0.00951433
**IPC**	0.06022836	0.008335038	0.4407068
**OPC**	0	0	0
**iPhC**	0	0.03805547	0.2686134

IHC: inner hair cell; OHC: outer hair cell; eIHC: early inner hair cell; eOHC: early outer hair cell; DC1/2: Deiter cells from rows 1 and 2; DC3: Deiter cells from row 3; Hensen: Hensen cells; IPC: inner pillar cells; OPC: outer pillar cells; iPhC: inner phalangeal cells

Table S4. Expression of *Nrxns* in Mouse Utricle

All data is presented as log_{10}, from a Hertzano/Ament Lab scRNAseq dataset from P2 mouse utricle, generously provided in gEAR. Unpublished (Orvis et al., 2021).

	Nrxn1	*Nrxn2*	*Nrxn3*
**Type 1 Hair Cells**	0	0.8853568	1.936291
**Type 2 Hair Cells**	0	0.2792121	1.250149
**Supporting Cells**	0.05449306	0.02006748	0.1126775

Development • Supplementary information

Table S5. *Nrxn3* zebrafish RNA FISH probe sequences

**α-*nrxn3a***
PROBE 1 ACGTGCTCTGTTGTAGATGGGGAACCACTTTGTGATTGTTCTAAGACGGAAT
PROBE 2 GTTGGCCGATTCTGCAACGAAGAGGCCAACAACATCCCAGGTTTTGCACACA
PROBE 3 ATGATGGCGGATCAAGCCAAGGGCAAAGCACGAGAAGAGAATGTGGCCACTT
PROBE 4 GACCCCAAGATGAAGATCCAGGGCGATGTGGTGTTCAAGTGTGAAAACGTGG
PROBE 5 ACGCTGGATCCGATTTCGTTCGAGACGCCTGAGGCCTACATCAGCCTGCCTA
PROBE 6 AACGGCCTTATTCTCTTCACCCACGGCAAGCCACAGGAGAGAAAGGACGCCC
PROBE 7 GGCTGGAATCGGTTTATCTGTGACTGCACAGGCACTGGTTACTGGTCCCGCA
PROBE 8 TCAAAGAACAGTTACTTGAGTCTTGCCACCTTGCAGGCTTATACCTCCATGC
PROBE 9 GTGTTTAATCTCGGCAACGGGCCCAATGTAATCAAAGGCAACAGTGAACGAG
PROBE 10 CACACACTCAAAGTCGATGCCAAGGCAGTGAGTCAGGTCGTCAATGGCGCTA
PROBE 11 AACAACCTTCCGAAGCTCGTGGCCTCCAGAGAGGGCTTTAAAGGCTGCCTGG
PROBE 12 AGCGGCCAGATCGAGCGAGGATGCGAAGTTGGTTTCACCAAAGCAGATCTAA
**α-*nrxn3b***
PROBE 1 GTTCTCTACTTTGATGATGGAGGATATTGTGACTTCCTGCTTCTGAGTATTG
PROBE 2 GATGCCAAGCTCAAGTTGCGTTTTAGCGTGGACTGTGCAGAAACCACTATAA
PROBE 3 TCTGACAAGATGGTAAACGACAGTCACTGGCACTTTGCTACAATCAGCAGGC
PROBE 4 CCACAAAGGCAGTTCATGAAAATTGTCAGTGACCTGTATCTTGGAGGTGTCC
PROBE 5 CAAGACATTCGGACATCTGCCCTAACACTACCAGCAGCTAAAGAGATGCCAC
PROBE 6 GGGAGCCAAAAGGTCCGGTTAGAGATGGAAGGTTTTTGTACAGAGAACCCCT
PROBE 7 GAAAATGGAGGCAGTTGCAGCATGGCTGATGGTGAGGCTTACTGTGACTGCT
PROBE 8 AAAACAGGATATACAGGACGGTACTGCAACGAAGCTGTCAACAAAACCCCTG
PROBE 9 GACCCCAAAATGAAGCTACAAGGAGATATTGTGTTTAAATGTGAGAATGTCC
PROBE 10 ACCCTCGACCCCATCTCTTTTGAGACGGCGGAATCCTTCCTCGGCCTGCCGA
PROBE 11 AACGGACTCATCCTCTTCACGCAAGGTAAACCACAGGACAAAAAAGATTCTC
PROBE 12 CTTTATCTTTTGCTGGACATGGGATCAGGGACAATTAAAGTTAAAGCCACGC
PROBE 13 TCCCAACGTGCTTTTGGCTTGTTGATGGCTGCCACGTCACGTGAGTCTGCAG
PROBE 14 TCTGTCAGAGTTACGCGGCGTGGTAAAAACATCAAACTTATGGTGGATGATG
PROBE 15 GAGACTGGAATCCTGACGGAACGGCGATTTGCCTCCACGGCTCCGTCTAACT
PROBE 16 AAGAACGGAGACATCGAATTTTGCGAGCTCAATGCTCGTTTTGGGATGCGCT
PROBE 17 ATCGTGGCTGACCCTGTTACCTTCAAGACTAAAGGAAGCTATCTGGGACTTG
PROBE 18 GTGATTACTCGAGACGCAAGCAACACCCACACACTAAAAGTGGACGCTAAGT
PROBE 19 CGTGAAGGATTTCAAGGCTGCTTAGCTTCCATGGATCTAAATGGCCGTCTGC

Development • Supplementary information

Table S6. Primary and secondary antibodies used.

Primary antibody list

Antigen	Species; subtype	Concentration	Catalog or reference
Pan-MAGUK	Mouse; IgG1	1:500	Millipore MABN7
MYO7A	Rabbit	1:1,000	Proteus 25-6790
MYO7A	Mouse; IgG1	1:400 (mouse)<br>1:1,000 (zebrafish)	Developmental Study Hybridoma Bank 138-1
Otoferlin	Mouse; IgG2a	1:1,000	DSHB HCS-1
Calretinin	Mouse; IgG1	1:1,000	Swant 6B3
Paravalbumin	Mouse; IgG1	1:1,000	Millipore/Sigma MAB1572
SOX2	Rabbit	1:1,000	Abcam ab97959
Oncomodulin	Goat	1:400	ThermoFisher PA547832
$Ca_V1.3$	Rabbit	1:1,000	(Sheets et al., 2011)
GFP	Chicken	1:1,000	ThermoFisher A10262
Pan-CTBP	Mouse; IgG2a	1:1,000	Santa Cruz sc-3878
GluR2	Mouse; IgG2a	1:100	Millipore/Sigma MAB397
CTBP2	Rabbit	1:400	Synaptic Systems 192103

Secondary antibody and co-label list

Antigen	Species; fluorophore	Concentration	Catalog or reference
Mouse IgG2a	Goat; Alexa 488, Alexa 555, Alexa 647	1:1,000	ThermoFisher #A-21131, #A-2113, # A-21241
Mouse IgG1	Goat; Alexa 647, Alexa 555, Alexa 488	1:1,000	ThermoFisher #A-21240, #A-21422, #A21121
Mouse IgG	Donkey; Alexa 488	1:1,000	ThermoFisher # A-21202
Rabbit IgG	Donkey; Alexa 555	1:1000	ThermoFisher #A-31572
Rabbit IgG	Goat; Alexa 488, Alexa	1:1,000	ThermoFisher #A-11008, **#** A-

Development • Supplementary information

	555, Alexa 647		21428, #A21241
Goat	Donkey; Alex 647	1:500	ThermoFisher #A-21447
Chicken	Goat; Alexa 488	1:1,000	ThermoFisher #A-11039
F-actin	Phalloidin Alexa 488<br>Phalloidin Alexa 405	1:1000	ThermoFisher #A-12379<br>ThermoFisher #A-30104

Development • Supplementary information

References

Fabian, P., Tseng, K.-C., Thiruppathy, M., Arata, C., Chen, H.-J., Smeeton, J., Nelson, N. and Crump, J. G. (2022). Lifelong single-cell profiling of cranial neural crest diversification in zebrafish. *Nat Commun* 13, 13.

Kolla, L., Kelly, M. C., Mann, Z. F., Anaya-Rocha, A., Ellis, K., Lemons, A., Palermo, A. T., So, K. S., Mays, J. C., Orvis, J., et al. (2020). Characterization of the development of the mouse cochlear epithelium at the single cell level. *Nat Commun* 11, 2389.

Lush, M. E., Diaz, D. C., Koenecke, N., Baek, S., Boldt, H., St Peter, M. K., Gaitan-Escudero, T., Romero-Carvajal, A., Busch-Nentwich, E. M., Perera, A. G., et al. (2019). scRNA-Seq reveals distinct stem cell populations that drive hair cell regeneration after loss of Fgf and Notch signaling. *Elife* 8, e44431.

Orvis, J., Gottfried, B., Kancherla, J., Adkins, R. S., Song, Y., Dror, A. A., Olley, D., Rose, K., Chrysostomou, E., Kelly, M. C., et al. (2021). gEAR: Gene Expression Analysis Resource portal for community-driven, multi-omic data exploration. *Nat Methods* 18, 843–844.

Shi, T., Beaulieu, M. O., Saunders, L. M., Fabian, P., Trapnell, C., Segil, N., Crump, J. G. and Raible, D. W. (2023). Single-cell transcriptomic profiling of the zebrafish inner ear reveals molecularly distinct hair cell and supporting cell subtypes. *Elife* 12, e82978.
