# Supplementary material for: Presynaptic Nrxn3 is essential for ribbon-synapse maturation in hair cells
Source: Development. 2024 Oct 10;151(19):dev202723. doi: 10.1242/dev.202723 (PMC11488651; doi:10.1242/dev.202723)
Supplement: Supplementary file 1 [file develop-151-202723_review_history.pdf]

# Presynaptic Nrxn3 is essential for ribbon-synapse maturation in hair cells

Alma Jukic, Zhengchang Lei, Elizabeth R Cebul, Katherine Pinter, Yommi Tadesse, Amandine Jarysta, Sandeep David, Natalie Mosqueda, Basile Tarchini and Katie Kindt DOI: 10.1242/dev.202723

**Editor**: Steve Wilson

**Review timeline**

| | |
|---|---|
| Original submission: | 25 January 2024 |
| Editorial decision: | 28 March 2024 |
| First revision received: | 16 July 2024 |
| Accepted: | 28 August 2024 |

**Original submission**

First decision letter

MS ID#: DEVELOP/2024/202723

MS TITLE: Presynaptic Nrxn3 is essential for ribbon-synapse assembly in hair cells

AUTHORS: Alma Jukic, Zhengchang Lei, Elizabeth R Cebul, Katherine Pinter, Natalie Mosqueda, Sandeep David, Basile Tarchini, and Katie S Kindt

Apologies for the delay in making a decision on your manuscript. The referees' comments are appended below, or you can access them online: please go to BenchPress and click on the 'Manuscripts with Decisions' queue in the Author Area.

As you will see, the referees express considerable interest in your work, but have some significant criticisms and suggestions for improvements to your manuscript. Although two of the reviewers support publication in Development, reviewer 1, who has developmental biology expertise, thinks that the study does not have a sufficiently developmental focus for this journal. However, given the overall level of enthusiasm, I would like to give you the opportunity to revise your study, emphasising, where appropriate, the developmental insights from your work. Please note that Development will normally permit only one round of major revision. If it would be helpful, you are welcome to contact us to discuss your revision in greater detail. Please send us a point-by-point response indicating your plans for addressing the referees’ comments, and we will look over this and provide further guidance.

Please attend to all of the reviewers' comments and ensure that you clearly highlight all changes made in the revised manuscript. Please avoid using 'Tracked changes' in Word files as these are lost in PDF conversion. I should be grateful if you would also provide a point-by-point response detailing how you have dealt with the points raised by the reviewers in the 'Response to Reviewers' box. If you do not agree with any of their criticisms or suggestions please explain clearly why this is so.

Reviewer 1

*Advance summary and potential significance to field*

© 2024. Published by The Company of Biologists under the terms of the Creative Commons Attribution License (https://creativecommons.org/licenses/by/4.0/). 

Jukic and colleagues describe roles for the adhesion protein Neurexin3 in hair cell synapse formation. The authors examine expression of nrxn3 genes in the zebrafish lateral line and inner ear hair cells, and examine the distribution of ribbon synapses in nrxn3 mutants, finding a significant reduction in pre and post synaptic structures. Analysis of Nrxn3 mutants in mice show a similar loss of synapses. Imaging of calcium responses in lateral line hair cells demonstrates a significant reduction in both presynaptic and postsynaptic activity in response to water jet stimuli.

*Comments for the author*

The authors present nicely designed experiments with appropriate quantitative analysis and the manuscript is clearly written. While the work presented is carefully done, the study as a whole seems incomplete.
Moreover there is little analysis of developmental mechanisms, making the work, as is, potentially more suitable for a journal focussed on neural function.

Major points:

Neurexins have been promoted as part of the mechanism to promote synapse specificity, yet in these studies the authors have not examined this issue, leaving analysis incomplete. As noted in the discussion section previous studies have identified connectivity rules for the lateral line. Has this specificity been lost in nrxn3 mutants? Have the number of neurons innervating neuromasts been altered? Are post-synaptic neurons still only responsive to water jet in a specific direction?

Neurexin 3 has a number of splice isoforms in addition to the alpha and beta forms mentioned in this manuscript (e.g. Schreiner et al 2014, Treutlein et al 2014). More detail is needed with respect to these isoforms. Are these isoforms present in hair cells? Where do mutations fall with respect to these potential alternate splicing events? What forms are detected by HCR - could that explain the relatively weak nonsense-mediated decay?

The authors state that the reduction in synapses has no behavioral defects. However the assay they performed appears to have weak discriminatory ability and appears underpowered. Could the assay detect a 50% reduction in activity? I'm not sure that this is a critical point in the study and think claims of robustness or redundancy related to the behavior will require additional analysis with greater sensitivity.

Prior studies indicate that the Nrxn3 interacting proteins Nlgn1 and Nlgn3 are differentially expressed along the mediolateral axis of mouse IHCs and result in synapse loss and reduced hearing. The authors present no analysis of the distribution of remaining synapses in Nrxn3 mouse mutants or analysis of hearing (ABR, wave ! amplitude).

Expression analysis is also incomplete. Are nrxn1 or 2 expressed in the system? Are nlgn1 and 3 similarly conserved in zebrafish? Is Nrxn3 expressed in mouse hair cells?

Minor points:
Figure numbering perhaps could be rearranged as they are presented out of order, e.g S3 before S2, Fig 6B after the rest of the figure.

Antibodies for otoferlin are referenced in the methods but not in the text.

How are organ outlines determined (Fig S1, S2)? Why are there some nrxn3+ puncta outside of the indicated area (S2)? Is there a developmental difference between nrxn3a and nrxn3b (3a expressed in younger peripheral hair cells)?

Insets (e.g. Fig 3 C,F; Fig 4E-h) are not always easy to identify in the main figure - these should be labeled or presented in a systematic way (e.g. from left to right, top to bottom)

<u>Reviewer 2</u>

*Advance summary and potential significance to field*

© 2024. Published by The Company of Biologists under the terms of the Creative Commons Attribution License (https://creativecommons.org/licenses/by/4.0/).

In this manuscript, Jukic and Lei et al. provide a beautifully written and illustrated report on the function of Nrxn3 in hair cell ribbon synapse formation an important process that is poorly understood.

*Comments for the author*

In this manuscript, Jukic and Lei et al. provide a beautifully written and illustrated report on the function of Nrxn3 in hair cell ribbon synapse formation an important process that is poorly understood. Genetics and detailed imaging and histology are used to analyze Nrxn3a and 3b double mutants (some data for single mutants is also included). The authors characterize different features of lateral line afferent pre- and post-synapses at different stages, and show some functional deficits (at the level of calcium transients, but not behavior). Especially revealing are the changes in the distribution and size Cav1.3 channels. The authors include a small amount of data from cochleae from a Nrxn3 mouse mutant to reveal possible Nrxn3 functional conservation. Overall, the manuscript and data analyses are excellent. My concerns are related to points of validation in their reagents and animal models or are otherwise minor.

1. In the Introduction, line 87, the authors note how the research “will inform future research aimed to rebuild synapses and restore hearing after auditory synaptopathy.” While this may be true, the authors should also note how their research also makes important contributions to knowledge of vestibular afferent synapse formation, an area in dire need of knowledge.
2. For the RNA FISH studies in figure 1, the probes look specific and the expression data in the gEAR indicates that Nrxns are expressed in hair cells. But the authors do not show the probes are specific. In some ways the data in Fig. S2 suggest specificity if you assume nonsense mediated decay is happening.
Regardless, the authors should show some kind of control for these probes to ensure no non-specific binding.
3. The authors do not show confirmation of protein loss in their Nrxn mutants (probably due to lack of good antibodies), nor did the authors attempt any sort of experiments using morpholinos or CRISPR (as examples) to try and phenocopy the effects. So, at this point the characterization of their mutants feels somewhat incomplete. This limitation should be noted in the Discussion.
4. This is somewhat minor, but it would be helpful to see if there are general defects in neuromast hair and supporting cells in the Nrxn double mutants.
I appreciate the hair cell counts in figure 2G, but just one image showing normal hair and supporting cell position and contacts with each other (even just in the supplement) would show that other aspects of neuromast development are unaffected.
5. The data indicate that, in their zebrafish double mutants, total postsynapse numbers are normal at early stages, but reduced at later stages. This could mean that afferent fibers/neurons are dying, which would impact the interpretation of their GCaMP data. It would be helpful if the authors could show whether the loss of Nrxn3a and b led to any reduction in the postsynapse arbor using an afferent marker (or possibly the baseline fluorescence from the postsynapse GCaMP line, e.g. fig. 6 G, H, if that’s bright enough?).
6. The mouse data overall is nice, but incomplete. In order to say that synapse “assembly” is altered in the Nrxn3 CKO line, the authors would need to look at a much earlier stage, like P12-P15, when synapse formation is complete. If the authors are unable to do this, they should temper their language and just say the mutants have fewer synapses. As it is, they cannot distinguish between loss of synapse development vs loss of synapse maintenance in the Nrxn3 CKO. They are probably right, but the staging just isn’t quite right to be sure.
7. The authors need to validate the Nrxn3 CKO mouse line by showing hair cell-specific loss of Nrxn3 with Atoh1-Cre.
8. For the fig. 6 on page 9, please note the GCaMP drivers in the main text.
IMO it will make it more informative and interesting to read.
9. Page 13, line 362. “Each subtype synapses onto…” Based on the recent paper from Uli Muller’s lab (Siebald et al), the authors may want to consider using “loosely” in place of “likely”....and definitely cite that paper.
10. The last sentence on page 14 uses “straightforward” twice. Consider revising.

<u>Reviewer 3</u>

© 2024. Published by The Company of Biologists under the terms of the Creative Commons Attribution License (https://creativecommons.org/licenses/by/4.0/).

*Advance summary and potential significance to field*

The auditory sensory cell communicates to the afferent auditory neurons using an electron dense body, known as ribbon body. The molecular composition of the ribbon still remains elusive. Here, Jukic and coll identified the neurexin as key player to assemble functional ribbon synapses. Thus, this study brings a substantial contribution in the understanding of the acoustic cues coding.

*Comments for the author*

In this study, Jukic and coll examined the expression and function of the neurexin 3 (Nrxn3) in the sensory hair cells from zebrafish. Using RNA-FISH, they demonstrated the expression of alpha-nrxn3a and alpha-nrxn3b in hair cells from the lateral line neuromast. Then, they found out a disruption in the synapse assembly in the nrxn mutants using confocal microscopy. Of note, they extended their finding to the mouse auditory sensory cells. Finally, they employed calcium indicator GCamp6 dynamics in hair cells and afferent neurons to probe mechanotransduction and synaptic activity. They showed preserved mechanotranduction but reduced pre- and post-synaptic responses in the nrxn3a; nrxn3b mutant zebrafish, consistent with the loss of paired synapses. However, the authors stressed-out that the depressed synaptic responses did not translate into an obvious behavioral deficit. Nevertheless, the finding made by Jukic et al., is pretty clear: the neurexins are required for the synapse assembly and function. The authors put a lot of effort in documenting the expression of the nrxn isoforms in hair cells, to count the consequence of the nrxn deletion on the number of pre and post synaptic elements, providing gorgeous pictures from zebrafish lateral line and inner ear up to cochlear hair cells. Finally, the calcium imaging makes this study pretty much solid.

I was wondering how the average of the synaptic have been made in mouse. For instance, in the legend 70 IHCs were used for control from 4 animals. What represents each dot in the figure 5C to E? What if the authors give the same weight to each sample (each mouse or cochlea would give a mean and then do a grand average)? In addition, it seems that the number of synapse does not differ between apical and middle cochlear region, which is a bit surprising (see Meyer et al., 2009, fig 1B: the number of synapse changes along the tonotopic axis). I am not convinced that the authors really examined different areas along the tonotopic axis. Could the author indicate what are the frequency-corresponding regions they are looking at? In addition, did the authors check the CRE expression in their construct? Finally, may the author provide high mag of the synapse as they did for the zebrafish?

Why the GCaMP6 linearly drops in fig S8?
In fig S9 D, there are 4 ROI but 6 traces in F. Maybe the authors could assign a corresponding number or letter between the ROI in Fig S9 A, D, G, J and the calcium increases in Fig S9 C, F, I and L, respectively. Do the authors know why there is such a large difference in the calcium responses in both genotype in Fig S9 C and F in hair cell (one ROI shows a huge increase in calcium). A naive question, why the kinetics of the calcium retrieval seem to be faster in afferent neuron in comparison to the hair cell? In the Fig 6 and FigS9, the hair cells in the prestim are easily discerned by the plasma membrane. Is the black area at the center of the hair cells the nucleus?
I did not catch why the authors do not merge the fig S9 to fig 6 (individual ROI traces may be added to the mean, unless it becomes overloaded panels). In FigS9, scale bar is missing in Fig S9 A-E as well as in the legend (I guess it is 5 µm).

---

**First revision**

Author response to reviewers' comments

We would like to thank all the reviewers for their valuable feedback on our manuscript. Based on your comments, we have made a significant revision to our manuscript. This includes 1 additional main figure, as well as 6 additional supplementary figures. Through these additions we have been

© 2024. Published by The Company of Biologists under the terms of the Creative Commons Attribution License (https://creativecommons.org/licenses/by/4.0/). 

able to address the majority of your concerns and have greatly strengthened our findings. Please find our point-by-point response to all comments below in blue.

Reviewer 1 Advance Summary and Potential Significance to Field:
Jukic and colleagues describe roles for the adhesion protein Neurexin3 in hair cell synapse formation. The authors examine expression of nrxn3 genes in the zebrafish lateral line and inner ear hair cells, and examine the distribution of ribbon synapses in nrxn3 mutants, finding a significant reduction in pre and post synaptic structures. Analysis of Nrxn3 mutants in mice show a similar loss of synapses. Imaging of calcium responses in lateral line hair cells demonstrates a significant reduction in both presynaptic and postsynaptic activity in response to water jet stimuli.

Reviewer 1 Comments for the Author:
The authors present nicely designed experiments with appropriate quantitative analysis and the manuscript is clearly written. While the work presented is carefully done, the study as a whole seems incomplete. Moreover there is little analysis of developmental mechanisms, making the work, as is, potentially more suitable for a journal focused on neural function.

Broadly, the work from our group seeks to link development and function in the realm of sensory neuroscience. It is true-we have submitted this paper to Development. Therefore, in our revision, we have made a tremendous effort to highlight the developmental aspects of our study. For example, we have added a developmental emphasis to our abstract, and Intro/Discussion summaries.

In addition, we have further explored the developmental mechanisms of Nrxn3, by examining the role of Nrxn3 in synapse specificity more thoroughly (see below and the newly added Fig 4).

Major points:

Neurexins have been promoted as part of the mechanism to promote synapse specificity, yet in these studies the authors have not examined this issue, leaving analysis incomplete.

Analysis of synapse specificity:
Previously our pilot studies on synapse specificity did not reveal an obvious role for Nrxn3 in hair-cell synapse specificity. But in our revision, we decided to revisit this question by looking more closely at whether Nrxn3 plays a role in the most well-defined example of synapse specificity in the lateral line-selective innervation based on the direction of flow sensed by these hair cells-anterior (P to A) or posterior (A to P) directed flow. In our initial submission we showed that loss of Nrxn3 leads to disrupted synapses in both (P to A) and (A to P) hair cells. Upon further investigation we present 3 new lines of evidence to suggest that loss of Nrxn3 has a greater impact on (P to A) synapses. This data is shown in 2 new Figures, Fig 4 and Fig S16.

1. We found that while both (P to A) and (A to P) hair cells show a dramatic reduction in complete synapses per hair cell in *nrxn3* mutants, (P to A) cells have even fewer synapses compared to (A to P) cells (Fig 4C).
2. We used microinjection approaches to label individual afferent neurons and the connections they make with lateral-line hair cells. Using this approach, we found that in *nrxn3* mutants afferent terminals innervating (P to A) cells are less selective compared to terminals that innervate (A to P) cells (Fig 4G).
3. We further analyzed our calcium imaging results and looked more closely at the number of active postsynaptic sites based on the direction of the stimulus-anterior (P to A) or posterior (A to P) directed flow. We found that in *nrxn3* mutants there were fewer (P to A) active sites compared to (A to P) active sites (Fig S16B).

Each of these changes is subtle, yet significant, and together these results indicate that Nrxn3 may play a larger role in synapse maturation in hair cells that sense anterior (P to A) flow compared to hair cells that sense posterior (A to P) flow.

As noted in the discussion section, previous studies have identified connectivity rules for the lateral line. Has this specificity been lost in nrxn3 mutants? Have the number of neurons innervating neuromasts been altered? Are post-synaptic neurons still only responsive to water jet in a specific direction?

© 2024. Published by The Company of Biologists under the terms of the Creative Commons Attribution License (https://creativecommons.org/licenses/by/4.0/).

Number of neurons innervating neuromasts:
During the revision process we attempted to develop a light microscopy-based approach to determine how many neurons innervated each neuromast. For this experiment we used transgenic zebrafish expressing a photoconvertible Kikume in lateral-line afferents. We converted Kikume in the terminals and looked the next day to see how many afferent cell bodies were labeled. Using this approach, we were only able to detect 2-3 afferent cell bodies per converted terminal (neuromast), not the 4-6 that has been shown using SBF-SEM. The neurons we detect are likely major neurons that innervate many hair cells and occupy the majority of the terminal. Based on this work we concluded that the SBF-SEM is needed to determine the innervation pattern and number of afferents connected to each neuromast. We have stated this limitation (not knowing how many afferents connect to each neuromast without SBF-SEM) in our discussion.

Instead, we looked closer at the afferent neurons in the posterior lateral line more closely in 3 ways.

1. We counted the number of afferent neurons in the posterior lateral line ganglia. We found a similar number of cell bodies in wild type and *nrxn3* mutants (Fig S11A). This provides mechanistic evidence that neurons are not dying after synapses fail to form.
2. We quantified the area occupied by the 4-6 neurons forming connections with lateral line neuromasts. We found that the area of occupied by the terminals was similar in wild type and nrxn3 mutants (Fig S11B-D).
3. We performed microinjection to label single afferent and examined how selectively each afferent terminal connected to lateral line hair cells based on orientation (P to A) vs (A to P) (Fig 4D-G). See more info on these results above.

Responsiveness of postsynaptic neurons based on direction of the stimulus:
Yes! we examined this more closely. See our response above.

Neurexin 3 has a number of splice isoforms in addition to the alpha and beta forms mentioned in this manuscript (e.g. Schreiner et al 2014, Treutlein et al 2014). More detail is needed with respect to these isoforms. Are these isoforms present in hair cells? Where do mutations fall with respect to these potential alternate splicing events? What forms are detected by HCR - could that explain the relatively weak nonsense-mediated decay?

Splice isoform expression and lesioning:
This is a great point. We have clarified information regarding *nrxn3* splice variants in both the text and via a supplemental figure (Fig S2). In the text (results and methods) we now state that our germline *nrxn3* zebrafish mutants lesion an obligatory exon that is present in all known splice variants of alpha-Nrxn3. We also state that the majority of our HCR probes (12/13 for *nrxn3a* and 18/19 for *nrxn3b*) recognize exons present in all slice variants of alpha-*nrxn3*. Based on the location of these probes, primarily in obligatory exons, probe location is not sufficient to explain the low level of nonsense mediated decay in our germline mutants.

In Fig S2, based on human and zebrafish publications on *Nrxn3* splicing, we highlight obligatory (green) and alternatively spliced (yellow) exons of *nrxn3a* and *nrxn3b*. In this figure, the exact location of the HCR probes are indicated (pink), as well as locations of the germline mutations. Probe sequences are also listed in Supplemental Table 1. We feel that this additional text, figure and table provide the critical and relevant information on *nrxn3* splice variants.

One concern we were unable to address in our revision was whether any of the alternatively splice exons are part of the transcripts we detect in hair cells. Based on our discussion with Molecular instruments although several of the alternatively spliced exons are very small, it could be possible to detect RNA FISH signals using exon-specific probe. Unfortunately, is it beyond the scope of this paper to examine all possible *nrxn3* splice variants. Perhaps RNA FISH, along with long-read scRNAseq could be used to study *nrxn3* splice variants more closely in future work.

Although we were unable to determine what splice variants were present in hair cells, we did perform one additional experiment to test whether *beta-nrxn3* plays a role in synapse maturation in hair cells. For this work we created F0 *beta-nrxn3* crispant zebrafish (first generation crispr-cas9 mutants). We found that these mutants had no synaptic phenotype. This provides strong evidence

© 2024. Published by The Company of Biologists under the terms of the Creative Commons Attribution License (https://creativecommons.org/licenses/by/4.0/).

that *alpha-nrxn3* and not *beta-nrxn3* plays a role in synapse maturation in zebrafish hair cells. This data is in Fig S10L-O.

The authors state that the reduction in synapses has no behavioral defects. However the assay they performed appears to have weak discriminatory ability and appears underpowered. Could the assay detect a 50% reduction in activity? I'm not sure that this is a critical point in the study and think claims of robustness or redundancy related to the behavior will require additional analysis with greater sensitivity.

Weak discriminatory ability of the acoustic startle assay:
In a previous study we used the same behavioral assay/apparatus. We characterized a single mutant with 60% reduction in startle response and a double mutant with a complete loss in startle response (Fig 5A; https://elifesciences.org/reviewed-preprints/89719#s2).This suggests that this assay be used to pick up behavioral responses that are reduced, as well as absent.

Power of zebrafish acoustic startle assay:
In our previously published study (Fig 5A; https://elifesciences.org/reviewed-preprints/89719#s2), 20 larvae were sufficient to detect a 60% reduction. But, to add power to our analysis, we nearly doubled the N in our dataset (18 to 29 control and 21 to 40 *nrxn3a; nrxn3b* animals). After adding additional animals, we verified there was no difference in startle response (P values for each stim, high p = 0.238, med p = 1.0, low p = 1.0) (also see Fig 8C).

Claims of robustness of redundancy:
We have removed statements referring to robustness or redundancy related to this behavior. In addition, in the discussion, we highlight that additional, more sensitive behavioral analyses are needed to fully characterize *nrxn3* mutants.

Prior studies indicate that the Nrxn3 interacting proteins Nlgn1 and Nlgn3 are differentially expressed along the mediolateral axis of mouse IHCs and result in synapse loss and reduced hearing. The authors present no analysis of the distribution of remaining synapses in Nrxn3 mouse mutants or analysis of hearing (ABR, wave 1 amplitude).

Distribution of IHC synapse loss in mouse *Nrxn3* mutants:
Although we were able to execute high quality immunostains of ribbon synapses in mouse IHCs, the mediolateral quantification of synapses proved to be beyond our level of expertise. Even in the Ramirez et al Nlgn1/3 study did not assess synapse loss along this axis (only Nlgn1/3 protein localization). We have stated in our discussion the future that mediolateral quantification should be performed, but it will have to be done by one of the few labs capable of this analysis.

Mouse ABR measurements:
In our revision we have added mouse ABR measurements, including analysis of wave-I amplitude and latency in our mouse *Nrxn3* mutants. Our ABR results show surprisingly normal ABR thresholds and no changes to the wave-I amplitude or latency in *Nrxn3* mutants compared to controls. This data is now in our results and in Fig 8A-B and Fig S17.

Expression analysis is also incomplete. Are nrxn1 or 2 expressed in the system? Are nlgn1 and 3 similarly conserved in zebrafish? Is Nrxn3 expressed in mouse hair cells?

*Nrxn1-3* and *Nlgn1-3* mRNA expression analysis:
Considerable additional work would be needed to assess the expression in situ of all *nrxns* and *nlgns* in mouse and zebrafish. Zebrafish have 6 *Nrxns* (*nrxn1a, nrxn1b, nxn2a, nrxn2b, nrxn3a, nrxn3b*) and 5 *Nlgns* (*nlgn1, nlgn2a, nlgn2b, nlgn3a, nlgn3b*). But we agree that some additional, comprehensive information on *Nrxn* mRNA expression would be helpful to the reader. Therefore, we put together a comprehensive figure (Fig S1) based on previously published scRNAseq expression of all *Nrxns* detected in the zebrafish inner ear and lateral line, as well as *Nrxns* detected in the mouse auditory and vestibular epithelium. We feel that the addition of Fig S1 is a nice reference guide to readers and those interested in *Nrxn* expression in zebrafish and mouse hair-cell epithelia. Fig S1 shows that *Nrxn3* is expressed in auditory and vestibular hair cells in mice. In addition, it shows that *Nrxn2* is also expressed in mouse hair cells-we now discuss this caveat in our discussion.

© 2024. Published by The Company of Biologists under the terms of the Creative Commons Attribution License (https://creativecommons.org/licenses/by/4.0/).

We did not include a similar Figure on *Nlgns* in the lateral line or inner ear afferent neurons 1) because the scRNAseq datasets are not as robust and 2) the data is convoluted by the fact that *Nlgns* could be used in the periphery for contact with hair cells, or more centrally to form connections present there.

Do Ngln1/3 play a conserved role at hair cell synapses in zebrafish?
We have used a crispant analysis to create F0 *nlgn1;nlgn3a;nlgn3b* triple crispants. We were not able to see convincing synapse phenotypes in these fish. We are currently growing up these fish and will use them in future studies. Thus, at this point we are uncertain if which Nlgns work (if any) pair with Nrxn3 at ribbon synapses in zebrafish hair cells.

Minor points:
Figure numbering perhaps could be rearranged as they are presented out of order, e.g S3 before S2, Fig 6B after the rest of the figure.

We made changes to ensure that the figures and panels are presented in order.

Antibodies for otoferlin are referenced in the methods but not in the text.

We used an Otoferlin antibody to co-label hair cells along with our Cav1.3 and CTBP label in Fig 5. The label is not shown in the images, but we now state in the legend that an Otoferlin co-label was used to visualize hair cells (not shown).

How are organ outlines determined (Fig S1, S2)? Why are there some nrxn3+ puncta outside of the indicated area (S2)? Is there a developmental difference between nrxn3a and nrxn3b (3a expressed in younger peripheral hair cells)?

Method to create organ outlines:
All organ and cell outlines are determined by a co-label that is either shown or not. For the RNA FISH experiments a transgenic line expressing GCaMP6s in hair cells, *myo6b:memGCaMP6s* (Fig 1, Fig S4, Fig S9) or RNA FISH co-label with *otofb* mRNA (hair cell marker (Fig S3) was used to create the outlines. In the immunostaining figures MYO7A, Otoferlin, Parvalbumin or Oncomodulin label (not shown, (Figs 2,3,5,6,S7,S10,S13)) were used to create the outlines. Each figure legend now states what label (and whether it is shown or not) was used to draw the outlines.

*nrxn3a* puncta outside of hair cell domain in neuromasts:
There are indeed *nrxn3a* puncta outside of the hair-cell domain in lateral-line organs. These puncta are likely in the supporting cells or glia that surround hair cells. In our results we now point out this expression in the results, as well as with arrows in Fig 1F.

*nrxn3a* vs *nrxn3b* mRNA expression based on cell stage:
We tried to take a closer look at the number of *nrxn3a* and *nrxn3b* mRNA based on hair-cell location, but our images were not good enough to clearly define and segment peripheral and central hair cells. In addition, the *nrxn3a* puncta are smaller and more numerous compared to the *nrxn3b* puncta making this type of comparison difficult. Further a developmental analysis is challenging as *nrxn3a* puncta are also likely in supporting cells; a subpopulation of supporting cells can act as precursor for hair cells. The expression of *nrxn3a* in supporting cells makes it difficult to conclude whether Nrxn3 plays a role early in development (supporting cell to hair-cell transition) or whether it plays a role specifically in supporting cells themselves.

Insets (e.g. Fig 3 C,F; Fig 4E-h) are not always easy to identify in the main figure - these should be labeled or presented in a systematic way (e.g. from left to right, top to bottom)

Thank you for this suggestion. All the insets are now displayed in the same order, from left to right.

***** Reviewer 2 Advance Summary and Potential Significance to Field:

© 2024. Published by The Company of Biologists under the terms of the Creative Commons Attribution License (https://creativecommons.org/licenses/by/4.0/).

In this manuscript, Jukic and Lei et al. provide a beautifully written and illustrated report on the function of Nrxn3 in hair cell ribbon synapse formation, an important process that is poorly understood.

Reviewer 2 Comments for the Author:

In this manuscript, Jukic and Lei et al. provide a beautifully written and illustrated report on the function of Nrxn3 in hair cell ribbon synapse formation, an important process that is poorly understood. Genetics and detailed imaging and histology are used to analyze Nrxn3a and 3b double mutants (some data for single mutants is also included). The authors characterize different features of lateral line afferent pre- and post-synapses at different stages, and show some functional deficits (at the level of calcium transients, but not behavior). Especially revealing are the changes in the distribution and size Cav1.3 channels. The authors include a small amount of data from cochleae from a Nrxn3 mouse mutant to reveal possible Nrxn3 functional conservation. Overall, the manuscript and data analyses are excellent. My concerns are related to points of validation in their reagents and animal models or are otherwise minor.

1.In the Introduction, line 87, the authors note how the research “will inform future research aimed to rebuild synapses and restore hearing after auditory synaptopathy.” While this may be true, the authors should also note how their research also makes important contributions to knowledge of vestibular afferent synapse formation, an area in dire need of knowledge.

Great point! In the abstract, introduction and discussion, we have expanded these future research statements to include the vestibular system.

2. For the RNA FISH studies in figure 1, the probes look specific and the expression data in the gEAR indicates that Nrxns are expressed in hair cells. But, the authors do not show the probes are specific. In some ways the data in Fig. S2 suggest specificity if you assume nonsense mediated decay is happening. Regardless, the authors should show some kind of control for these probes to ensure no non-specific binding.

Probe specificity:
Currently the standard procedure for RNA FISH controls is to include a *dapB* (bacterial probe) plus amplifiers. We have repeated our *nrxn3* RNA FISH experiments with *dapB* negative control probes and *otofb* positive control probes. We then acquired new images using the same settings for each channel. Using this approach, we observe 0-2 puncta with our *dapB* probes (Fig S4).

Nonsense mediated decay:
It is true that we do see less RNA FISH puncta in our *nrxn3* mutants, indicating some nonsense-mediated decay of the *nrxn3* mRNAs. Lower RNA FISH signal in the mutants is also good evidence that our probes are specific.

Additional *Nrxn* expression verification:
In addition to RNA FISH we have also created a new Figure, Fig S1 that summarizes previously published scRNAseq data on *Nrxn* expression in zebrafish and mouse hair-cell epithelia.

3.The authors do not show confirmation of protein loss in their Nrxn mutants (probably due to lack of good antibodies), nor did the authors attempt any sort of experiments using morpholinos or CRISPR (as examples) to try and phenocopy the effects. So, at this point the characterization of their mutants feels somewhat incomplete. This limitation should be noted in the Discussion.

Verification of Nrxn3 protein loss in zebrafish mutant:
We had not had any luck localizing Nrxn3 to the presynapse in zebrafish hair cells via immunohistochemistry. We have tried 4 antibodies against Nrxn3 using immunohistochemistry: Aviva (OAEB00576, ARP76858_P050), Sigma (HPA002727) and Synaptic Systems (175 303). Consistent with other all studies on Nrxns (with KO controls), Nrxn immunostaining does not work. We also used Aviva (ARP76858_P050), Sigma (HPA002727), Synaptic Systems (175 303), Protein Tech (21849) and Life Tech (PA5-115673) Nrxn3 antibodies on western blot. We were not able to see bands of the correct size in any of these antibodies when loading zebrafish tissue.
Only recent work using endogenously tagged Nrxns has revealed their localization:

© 2024. Published by The Company of Biologists under the terms of the Creative Commons Attribution License (https://creativecommons.org/licenses/by/4.0/).

https://www.nature.com/articles/s41467-023-40419-2
https://www.ncbi.nlm.nih.gov/pmc/articles/PMC6683742/pdf/JCB_201812076.pdf
In our results we recognize that we were unable to verify loss of gene product via immunohistochemistry. We suggest in our discussion that created endogenously tagged Nrxn3 in zebrafish is the way to go for localization.

Zebrafish *nrxn3* mutant phenotype verification:
For our revision we phenocopied our germline *nrxn3a; nrxn3b* mutants using a crispr-Cas9 “crispant” approach. For this analysis, we injected guides targeting alpha-*nrxn3a* and alpha-*nrxn3b* along with Cas9 protein and assessed synapses via immunostaining in injected (F0) larvae. In our *nrxn3a; nrxn3b* crispants we observed a 40% reduction in complete synapses. This verification provides additional, strong evidence that genetic lesioning of the *nrxn3a* and *nrxn3b* loci in zebrafish result in ribbon synapse loss in hair cells. This crispant result is stated in our results and is presented in a new figure, Fig S10A-K.

4.This is somewhat minor, but it would be helpful to see if there are general defects in neuromast hair and supporting cells in the Nrxn double mutants. I appreciate the hair cell counts in figure 2G, but just one image showing normal hair and supporting cell position and contacts with each other (even just in the supplement) would show that other aspects of neuromast development are unaffected.

Better morphology assessment:
To better assess the overall morphology and the supporting cells in *nrxn3a; nrxn3b* mutants, we performed an additional immunostain (SOX2 and MYO7A) to label supporting cell and hair cells. Images from this immunolabel is shown in Fig S6. We show both top down and side views of neuromast with hair cells and supporting cells labeled. These images provide good evidence that the overall morphology of these sensory organs in *nrxn3a; nrxn3b* mutants are largely normal. We quantified supporting cell numbers and did find slightly more supporting cells in *nrxn3a; nrxn3b* mutants compared to controls (44 vs 40 per neuromast) Fig S6D. This is reported in the manuscript.

5.The data indicate that, in their zebrafish double mutants, total postsynapse numbers are normal at early stages, but reduced at later stages. This could mean that afferent fibers/neurons are dying, which would impact the interpretation of their GCaMP data. It would be helpful if the authors could show whether the loss of Nrxn3a and b led to any reduction in the postsynapse arbor using an afferent marker (or possibly the baseline fluorescence from the postsynapse GCaMP line, e.g. fig. 6 G, H, if that’s bright enough?).

Quantification of afferent arbors and neuron cell counts:
We quantified the area of the afferent terminals using immunohistochemistry (Calretinin). Despite have fewer postsynapses, we did not observe a difference in arbor area between controls and *nrxn3* mutants Fig S11B-D.

We also counted the number of posterior lateral line neurons (using the *en.sill,hsp70l:GCaMP6s* transgenic line) and found that the number of neurons is not different between controls and *nrxn3* mutants Fig S11A. Similar arbor area and neurons numbers indicate that afferent neurons are not dying in the mutants.

Baseline GCaMP6 intensity in afferent terminals:
We quantified the baseline GCaMP6s intensity in hair bundles, at presynapses in the afferent terminals. We found no different in our baseline measurements between controls and *nrxn3* mutants. This data is shown in Fig S14A-C.

6.The mouse data overall is nice, but incomplete. In order to say that synapse “assembly” is altered in the Nrxn3 CKO line, the authors would need to look at a much earlier stage, like P12-P15, when synapse formation is complete. If the authors are unable to do this, they should temper their language and just say the mutants have fewer synapses. As it is, they cannot distinguish between loss of synapse development vs loss of synapse maintenance in the Nrxn3 CKO. They are probably right, but the staging just isn’t quite right to be sure.

© 2024. Published by The Company of Biologists under the terms of the Creative Commons Attribution License (https://creativecommons.org/licenses/by/4.0/).

IHC assembly vs maintenance timeline:
For our mouse assessment, it is true that many additional stages are needed to verify whether Nrxn3 is required for assembly/formation or maintenance of ribbon synapses in mouse IHCs. Therefore, we have tempered our language, and changed the title and summary statement to say maturation or organization instead of formation/assembly. Although we have kept "assembly" when referencing our developmental zebrafish results, when referring to the mouse IHCs we now state maturation or organization.

7.The authors need to validate the Nrxn3 CKO mouse line by showing hair cell-specific loss of Nrxn3 with Atoh1-Cre.

Verification of *Atoh1-Cre*:
The *Atoh1-Cre* line is a well-established, and widely used Cre line to manipulate hair cells in the auditory field. One line of evidence that the Cre line is working is that there is a IHC phenotype-ribbon synapse loss.
To further verify that the Atoh1-Cre line is working to edit the *Nrxn3* locus in the cochlea, we performed PCR on control and *Nrxn3* mutant mice. We observed removal of exon 18 in cochleae of *Atoh1-Cre; Nrxn3*$^{flox/flox}$ but not *Nrxn3*$^{flox/+}$ control mice. This provides strong evidence that the *Atoh1-Cre* line is working in the cochlea.

8.For the fig. 6 on page 9, please note the GCaMP drivers in the main text. IMO it will make it more informative and interesting to read.

In the main text, and in the figures we now include the drivers used our GCaMP6s lines (myo6b promoter and sill1 enhancer).

9.Page 13, line 362. "Each subtype synapses onto…" Based on the recent paper from Uli Muller's lab (Siebald et al), the authors may want to consider using "loosely" in place of "likelyc"....and definitely cite that paper.

We have changed likely to loosely. Good suggestion.

10.The last sentence on page 14 uses "straightforward" twice. Consider revising.

We are removed this repetition.

***** Reviewer 3 Advance Summary and Potential Significance to Field:
The auditory sensory cell communicates to the afferent auditory neurons using an electron dense body, known as ribbon body. The molecular composition of the ribbon still remains elusive. Here, Jukic and coll identified the neurexin as key player to assemble functional ribbon synapses. Thus, this study brings a substantial contribution in the understanding of the acoustic cues coding.

Reviewer 3 Comments for the Author:
In this study, Jukic and coll examined the expression and function of the neurexin 3 (Nrxn3) in the sensory hair cells from zebrafish. Using RNA-FISH, they demonstrated the expression of alpha-nrxn3a and alpha-nrxn3b in hair cells from the lateral line neuromast. Then, they found out a disruption in the synapse assembly in the nrxn mutants using confocal microscopy. Of note, they extended their finding to the mouse auditory sensory cells. Finally, they employed calcium indicator GCamp6 dynamics in hair cells and afferent neurons to probe mechanotransduction and synaptic activity. They showed preserved mechanotranduction but reduced pre- and post-synaptic responses in the nrxn3a; nrxn3b mutant zebrafish, consistent with the loss of paired synapses. However, the authors stressed-out that the depressed synaptic responses did not translate into an obvious behavioral deficit. Nevertheless, the finding made by Jukic et al., is pretty clear: the neurexins are required for the synapse assembly and function. The authors put a lot of effort in documenting the expression of the nrxn isoforms in hair cells, to count the consequence of the nrxn deletion on the number of pre and post synaptic elements, providing gorgeous pictures from zebrafish lateral line and inner ear up to cochlear hair cells. Finally, the calcium imaging makes this study pretty much solid.

© 2024. Published by The Company of Biologists under the terms of the Creative Commons Attribution License (https://creativecommons.org/licenses/by/4.0/).

I was wondering how the average of the synaptic have been made in mouse. For instance, in the legend 70 IHCs were used for control from 4 animals. What represents each dot in the figure 5C to E? What if the authors give the same weight to each sample (each mouse or cochlea would give a mean and then do a grand average)? In addition, it seems that the number of synapse does not differ between apical and middle cochlear region, which is a bit surprising (see Meyer et al., 2009, fig 1B: the number of synapse changes along the tonotopic axis). I am not convinced that the authors really examined different areas along the tonotopic axis. Could the author indicate what are the frequency-corresponding regions they are looking at? In addition, did the authors check the CRE expression in their construct? Finally, may the author provide high mag of the synapse as they did for the zebrafish?

N clarification and presentation of mouse IHC data:
The statement our of N for the mouse IHC quantification was not clear. In the legends we now state the following:
These findings were compiled from 4 animals from each genotype and from 2 independent liters and immunostains. Each dot represents the average synapse number from 1 imaging region (6-9 IHCs). Two imaging regions were examined per animal for each tonotopic region.

Tonotopic quantification of mouse IHC synapses:
Our mouse cochlear samples were dissected into three pieces-apex, mid and base at Jackson Laboratories. They were then stained and sent to the NIH for imaging. When imaging we always attempted to image centrally within each piece to ensure a unique tonotopic portion of the cochlea was imaged. Unfortunately, due to the way the samples were prepared, we were unable to map the tonotopic locations more accurately.

In addition to the Meyers 2009 work, we compared our study to the more recent work on the role of Nlgn1/3 in mouse IHC synapse maturation (Ramirez et al 2022). In this mouse IHC dataset, synapse numbers are only slightly different between frequency regions.
Synapse counts per IHC at P60 from apex to base: 8 kHz (15) 12 kHz (16) 16 kHz (17) 20 kHz (17) 24 kHz (16) 28 kHz (16).

Similar to our own synapse counts taken at P28 and P42, the work by Ramirez et al indicates that there are not dramatic differences in synapse counts across frequency region. Overall, we feel that our data shows that the phenotype we observe in Nrxn3 mouse mutants is robust and consistent across the cochlea.

Verification of *Atoh1-Cre*:
See response above to reviewer 2 and Fig S12.

High mag images of mouse IHC synapses:
Great suggestion. We have now included high magnification images of mouse IHC synapses in our main figure and supplement.

Why the GCaMP6 linearly drops in fig S8?

The initial linear drop in the MET responses is due to photobleaching of the GCaMP6s signal. This level of photobleaching does not interfere with the ability to detect differences in MET responses. We prefer to plot our raw data rather than photobleach-corrected traces.

In fig S9 D, there are 4 ROI but 6 traces in F. Maybe the authors could assign a corresponding number or letter between the ROI in Fig S9 A, D, G, J and the calcium increases in Fig S9 C, F, I and L, respectively.

Thank you for pointing out this inconsistency. We have corrected the number of ROIs and traces in our supplement (now Fig S15). Each GCaMP6s trace is now linked to a labeled ROI. In addition, [A to P] vs [P to A] responses are indicated.

Do the authors know why there is such a large difference in the calcium responses in both genotype in Fig S9 C and F in hair cell (one ROI shows a huge increase in calcium). A naive question, why the kinetics of the calcium retrieval seem to be faster in afferent neuron in comparison to the hair cell?

© 2024. Published by The Company of Biologists under the terms of the Creative Commons Attribution License (https://creativecommons.org/licenses/by/4.0/).

In the Fig 6 and FigS9, the hair cells in the prestim are easily discerned by the plasma membrane. Is the black area at the center of the hair cells the nucleus?

Source of heterogeneity of the evoked calcium signals:
We are only beginning to understand why there is so much heterogeneity in evoked calcium responses. This is something that we see consistently across genotypes, indicators and imaging systems. It is likely a combination of cell stage, synapse numbers and composition of channels at each synapse.

Kinetics of afferent vs hair cell GCaMP response:
The kinetics of the GCaMP6s responses in the afferent terminals are indeed faster than in the hair cell! This is something we observe consistently across imaging systems.

Hair cell nucleus:
Because our hair-cell GCaMP6 indicator is membrane localized, the nucleus does appear quite prominently. We now indicate in the figure legend that the center/black area of the memGCaMP6s-labeled hair cells is the nucleus.

I did not catch why the authors do not merge the fig S9 to fig 6 (individual ROI traces may be added to the mean, unless it becomes overloaded panels). In FigS9, scale bar is missing in Fig S9 A-E as well as in the legend (I guess it is 5 μm).

The Figure was indeed overly large without splitting a portion of it into the supplement.
We have added scale bar to the supplemental figure and legend (now Fig S14).

---

Second decision letter

MS ID#: DEVELOP/2024/202723

MS TITLE: Presynaptic Nrxn3 is essential for ribbon-synapse maturation in hair cells

AUTHORS: Alma Jukic, Zhengchang Lei, Elizabeth R Cebul, Katherine Pinter, Yommi Tadesse, Amandine Jarysta, Sandeep David, Natalie Mosqueda, Basile Tarchini, and Katie S Kindt
ARTICLE TYPE: Research Article

I am happy to tell you that the reviewers are happy with your revisions and your manuscript has been accepted for publication in Development, pending our standard publication integrity checks.

Reviewer 1

*Advance summary and potential significance to field*

The authors provide a comprehensive description of the roles for nrxn3 in the zebrafish lateral line system demonstrating a role for this molecule in synapse maturation. Overall the work is well done, clearly described and of significant advance.

*Comments for the author*

The authors have made considerable effort to address the points of my previous review, and offer reasonable justification for any additional points not addressed.

Reviewer 2

*Advance summary and potential significance to field*

© 2024. Published by The Company of Biologists under the terms of the Creative Commons Attribution License (https://creativecommons.org/licenses/by/4.0/). 

Our understanding of the mechanisms that control the formation, maturation, and maintenance of synapses between sensory hair cells and their afferent neural connections to the brain is quite limited. This report combines genetic and imaging approaches in zebrafish and mouse models to details the function of Nrxn3 in this process.

*Comments for the author*

I'm quite satisfied with the revisions the authors have made and appreciate their strong attention to detail. From my standpoint, no additional revisions are needed.

© 2024. Published by The Company of Biologists under the terms of the Creative Commons Attribution License (https://creativecommons.org/licenses/by/4.0/).
